# Design and Microwave Synthesis of New (5*Z*) 5-Arylidene-2-thioxo-1,3-thiazolinidin-4-one and (5*Z*) 2-Amino-5-arylidene-1,3-thiazol-4(5*H*)-one as New Inhibitors of Protein Kinase DYRK1A

**DOI:** 10.3390/ph14111086

**Published:** 2021-10-27

**Authors:** Khadidja Bourahla, Solène Guihéneuf, Emmanuelle Limanton, Ludovic Paquin, Rémy Le Guével, Thierry Charlier, Mustapha Rahmouni, Emilie Durieu, Olivier Lozach, François Carreaux, Laurent Meijer, Jean-Pierre Bazureau

**Affiliations:** 1 Institut des Sciences Chimiques de Rennes ISCR UMR CNRS 6226, Université de Rennes 1, Bât. 10A, Campus de Beaulieu, CS 74205, 263 Avenue du Général Leclerc, CEDEX, 35042 Rennes, France; bkhadije@yahoo.fr (K.B.); solene.guiheneuf@univ-rennes1.fr (S.G.); emmanuelle.limanton@univ-rennes1.fr (E.L.); ludovic.paquin@univ-rennes1.fr (L.P.); francois.carreaux@univ-rennes1.fr (F.C.); 2 S2Wave Platform, ScanMAT UMS 2001 CNRS, Université de Rennes 1, Bât. 10A, Campus de Beaulieu, CS 74205, 263 Avenue du Général Leclerc, CEDEX, 35042 Rennes, France; 3 ImPACcell Platform, SFR Biosit, Université de Rennes 1, Bât. 8, Campus Villejean, 2 Avenue du Prof. Léon Bernard, CS 34317, CEDEX, 35043 Rennes, France; remy.leguevel@univ-rennes1.fr (R.L.G.); thierry.charlier@univ-rennes1.fr (T.C.); 4 Institut de Recherche en Santé, Environnement et Travail, IRSET Inserm U1085, Université de Rennes 1, 9 Avenue du Prof. Léon Bernard, 35000 Rennes, France; 5 Laboratoire de Synthèse et de Catalyse, Université Ibn Khaldoun, Tiaret 14000, Algeria; m.rahmouni@mesrs.dz; 6 Perha Pharmaceuticals & ManRos Therapeutics, “From Sea to Pharmacy”, Hôtel de Recherche, 29680 Roscoff, France; emilie.durieu@univ-nantes.fr (E.D.); meijer@perha-pharma.com (L.M.); 7 Protein Phosphorylation & Human Disease Group, Station Biologique, 29680 Roscoff, France; olivier.lozach@univ-brest.fr

**Keywords:** microwave, Knoevenagel condensation, sulphur/nitrogen displacement, 1,3-thiazolidin-4-one, inhibition, protein kinase, DYRK1A, cell lines

## Abstract

Here, we report on the synthesis of libraries of new 5-arylidene-2-thioxo-1,3-thiazolidin-4-ones 3 (twenty-two compounds) and new 2-amino-5-arylidene-1,3-thiazol-4(5*H*)-ones 5 (twenty-four compounds) with stereo controlled *Z*-geometry under microwave irradiation. The 46 designed final compounds were tested in order to determine their activity against four representative protein kinases (DYR1A, CK1, CDK5/p25, and GSK3α/β). Among these 1,3-thiazolidin-4-ones, the molecules (5*Z*) 5-(4-hydroxybenzylidene)-2-thioxo-1,3-thiazolidin-4-one 3e (IC_50_ 0.028 μM) and (5*Z*)-5-benzo[1,3]dioxol-5-ylmethylene-2-(pyridin-2-yl)amino-1,3-thiazol-4(5*H*)-one 5s (IC_50_ 0.033 μM) were identified as lead compounds and as new nanomolar DYRK1A inhibitors. Some of these compounds in the two libraries have been also evaluated for their in vitro inhibition of cell proliferation (Huh7 D12, Caco2, MDA-MB 231, HCT 116, PC3, and NCI-H2 tumor cell lines). These results will enable us to use the 1,3-thiazolidin-4-one core as pharmacophores to develop potent treatment for neurological or oncological disorders in which DYRK1A is fully involved.

## 1. Introduction

**Figure 1:**
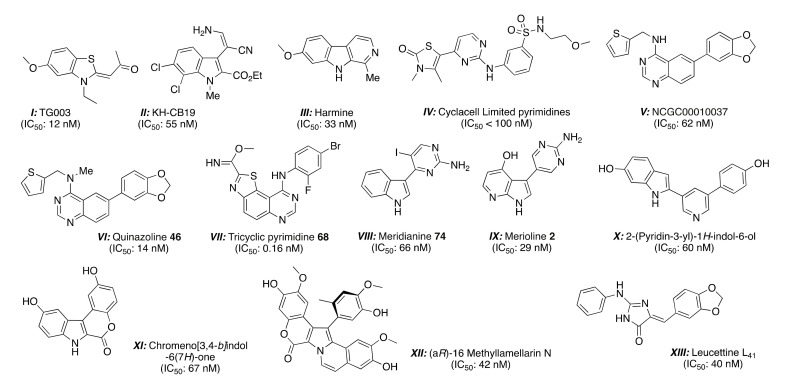
Reported small molecules as DYRK1A kinase inhibitors with their IC_50_ values in nM.

Protein kinases represent an important class of enzymes that play an important role in the regulation of various cellular processes. These enzymes catalyze protein-phosphorylation on serine, threonine, and tyrosine residues, which are frequently deregulated in human diseases. Only a small fraction of the 538 human kinases have been investigated as potential therapeutic targets [1]. Consequently, the search of protein kinase inhibitors represents an interesting target for the pharmaceutical industry for new therapeutic agents. Over the past decade, drug discovery has consisted of the empirical approach of random screening of large synthetic molecule libraries (or derivative of natural products) using high-throughput cell-based cytotoxicity assays to screening against clinical validated molecular targets. This approach has been successful in the area of oncology with small-molecule inhibitors of kinases, particularly in cancer progression and metastasis [2]. Among the 538 human kinases, DYRKs (dual-specificity tyrosine phosphorylation regulated kinases, 5 members) are a family of eukaryotic kinases that belong to a larger CMGC family of proline/arginine directed serine/threonine kinases. In this DYRK family, there are five mammalian subtypes (1A, 1B, 2, 3, and 4). The Dyrk1A gene is located within the human chromosome 21 Down Syndrome Critical Region (DSCR) [3]. According to recent literature, DYRK1A appears to be involved in various diseases, including Alzheimer’s disease (AD), Down syndrome (DS) [4], and cancer [5,6,7], and many efforts have evolved over the last five years toward the identification of potent and selective DYRK1A inhibitors [8,9]. Few potent and selective Dyrks inhibitors have been described (Figure 1). As example, TG003 (***I***) has been demonstrated as a potent inhibitor of DYRK1A (12 nM), as well as DYRK1B (130 nM) [10,11]. In 2011, KH-CB19 (***II***) was identified as a specific and interesting inhibitor of DYRK1A (33 nM) [12]. Harmine (***III***), a plant-derived β-carboline alkaloid, is an ATP-competitive DYRK1A (55 nM) and DYRK1B (166 nM) [13]. A series of thiazolone-substituted aminopyrimidines (***IV***) has been patented as DYRK1A inhibitors with potencies < 100 nM [14]. A structure activity relationship (SAR) study around the quinazoline core was carried out at the NIH, and their efforts culminated in the identification of compound (***V***) as a potent inhibitor of DYRK1A (62 nM) [15]. Revisiting the quinazoline chemistry, the same authors have also identified a new derivative (***VI***) (compound **46**: 14 nM) [16]. Recently, a series of tricyclic pyrimidines (***VII***) was evaluated for their ability to inhibit DYRK1A (0.16 nM) and DYRK1B [17]. Meridianines are marine alkaloids isolated from the South Atlantic tunicate *Aplidium meridanium*, and the synthesis of new analogues [18] substituted at the C-4 to C-7 position either by iodide atom or by nitro or amino groups induced the identification of meridianine **74** (***VIII***) as an interesting inhibitor of DYRK1A (compound **74**: 66 nM). The chemical structure resemblance between the marine alkaloids meridianines and variolin B encouraged the synthesis of a hybrid structure named meriolins [19], and compound (***IX***) provided good inhibition against DYRK1A (meriolin **2**: 29 nM). The V-shaped 2-(pyridin-3-yl)-1*H*-indol-6-ol (***X***) was discovered to inhibit DYRK1A (60 nM) [20].

Efforts for the synthetic preparation of new chromeno[3,4-*b*]indoles as Lamellarin D isosteres [21] have been developed, and compound (***XI***: 67 nM) emerged among the Lamellarin D derivatives. In 2013, the first successful synthesis and separation of the atropoisomeric (a*R*)- and (a*S*)-16-methyl lamellarins N and their different kinase inhibitory action against DYRK1A were reported [22]. The isomer (a*R*) (***XII***) showed potent but non-selective inhibition of DYRK1A (42 nM). The 2-amino imidazolone core is present in several marine sponges [23], such as polyandrocarpamines [24], dispacamides [25,26], aplysinopsine [27,28], clathridine [29], hymenialdisine [30,31,32,33,34,35,36], and Leucettamine B. This last alkaloid was isolated in 1993 from the marine calcareous sponge *Leucetta microraphis* Hæckel of the Argulpelu Reef in Palau [37], and no significant biological activity has been reported. From a biological screening, we discovered that the Leucettamine B analogues [38], named leucettines, exerted a selective inhibition toward protein kinases. A detailed structure-activity relationship (SAR) led to the identification of leucettine L_41_ (***XIII***) as good inhibitor of DYRKs and cdc2-like kinases (CLKs), which are involved in Alzheimer’s disease/Down syndrome [39]. We also demonstrated that leucettine L41 inhibits DYRK1A and CLKs in a cellular context and displayed neuropropective properties [40].

The 2-amino 5-arylidene-5*H*-thiazol-4-ones and their 5-arylidene rhodanine precursors are a class of five-membered heterocyclic rings (FMHRs) considered as “privileged scaffolds” in the medicinal chemistry community [41], and considerable work has been published over decades about their chemistry and biology. Some of them exhibited anti-inflammatory [42], anti-tumor effects [43], or have been identified as selective inhibitors of the dual specificity phosphatase (DSP) family [44].

Continuing in the effort to identify new DYRK1A inhibitors, we decided to explore the synthesis of new 5-arylidene-5*H*-thiazol-4-ones and new 5-arylidene-2-thioxo-1,3-thiazolidin-4-ones. We also studied their effects on some protein kinases (DYRK1A, CK1, CDK5-p25, and GSK-3α/β) and on six representative tumor cell lines. Herein, we present the building of this thiazolone/thiazolidinone library, mostly carried out under microwave irradiation, and the biological activities of these compounds.

## 2. Results and Discussion

### 2.1. Chemistry

**Figure 2:**
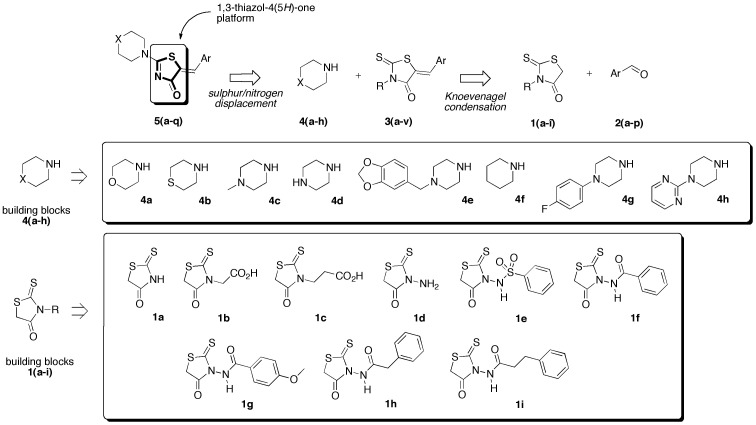
Retrosynthetic strategy toward new (5*Z*) 2-amino-5-arylidene-1,3-thiazol-4(5*H*)-ones **5a**–**q** based on the use of secondary cyclic amines building blocks **4a**–**h** in sulfur/nitrogen displacement reaction and the use of various 2-thioxo-1,3-thiazolidin-4-ones **1a**–**i** as starting platforms in Knoevenagel condensation.

**Figure 3:**
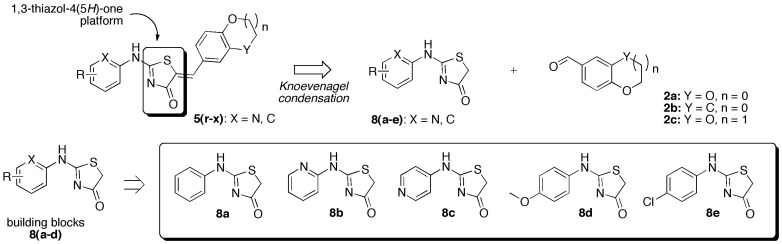
Retrosynthetic strategy toward new (5*Z*) 2-*N*-heteroarylamino-5-arylidene-1,3-thiazol-4(5*H*)-ones **5r**–**x** based on the use of aromatic aldehydes **2a**–**c** and various 2-*N*-heteroarylamino-1,3-thiazolidin-4-one **8a**–**e** as other starting platforms in Knoevenagel condensations.

Retrosynthetic strategy toward new (5*Z*) 2-amino-5-arylidene-1,3-thiazol-4(5*H*)-ones **5a**–**q** is based on the use of secondary cyclic amines building blocks **4a**–**h** in sulfur/nitrogen displacement reaction and the use of various 2-thioxo-1,3-thiazolidin-4-ones **1a**–**i** as starting platforms in Knoevenagel condensation (Figure 2 and Figure 3).

#### 2.1.1. Synthesis of 5-Arylidene-2-thioxo-1,3-thiazolidin-4-one Library

**Scheme 1:**
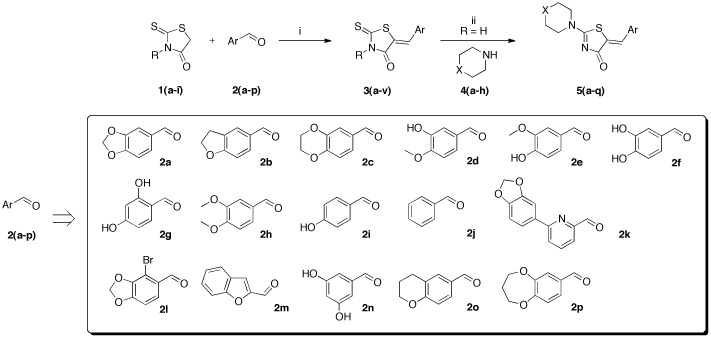
General synthetic approach for the (5*Z*) 5-arylidene-2-thioxo-1,3-thiazolidin-4-one derivatives **3**(**a**–**v**) and for the (5*Z*) 2-amino-5-arylidene-1,3-thiazol-4(5*H*)-one **5a**–**q**. Reagents and reaction conditions: (i) *n*-PrNH_2_ 2 eq, 80 °C, 30 min for compounds **3a**–**l** in open vessel using Synthewave^®^ 402 reactor for MWI; Et_3_N 0.1 eq or piperidine 0.1 eq or AcONa 1 eq, AcOH 0.1–6.6 eq, 85–150 °C, 20–210 min for compounds **3m**–**v** in closed vessel using Explorer 24^®^ CEM or Monowave^®^ 300 Anton-Paar reactors for MWI. (ii) **4a**–**h** 1.2 eq, MWI in Synthewave^®^ 402 microwave reactor, 80 °C, 20–60 min.

In order to synthesize a library of 5-arylidene-2-thioxo-1,3-thiazolidin-4-ones **3** (Scheme 1) for a systematic structure-activity relationship (SAR) study, we have examined the reactivity of 2-thioxo-1,3-thiazolidin-4-ones **1a**–**i** with a series of various aromatic aldehydes **2a**–**p**. We selected commercial aldehydes substituted by phenol function at various positions (**2f**, **2g**, **2i**, and **2n**) and/or methoxy groups (**2d**,**e**, **2h**) and cyclic ether function (**2a**–**c**, **2l**, **2o**, **2p**) to evaluate how the protic character and steric nature of the substituents on the phenyl ring will influence the biological activity of compounds **3**. We also examined the bio-isosteric replacement of the benzene ring of **3** with pyridine (**2k**) or benzofuran (**2m**) moieties. Compounds **3** are designed to evaluate whether the NH function in position *N*-1′ or a functional group with similar H-bond interactions will retain the activity.

For the 2-thioxo-1,3-thiazolidin-4-one platforms **1**, rhodanine **1a**, 2-(4-oxo-2-thioxo-thiazolidin-3-yl)acetic acid **1b** and 3-amino rhodanine **1d** are commercially available. Access to the 3-(4-oxo-2-thioxo-thiazolidin-3-yl)propanoic acid **1c** could be accomplished by a recent patented protocol [45]. For introduction of the arylsulfonamide functionality on the rhodanine moiety in compound **1e**, we used the Power’s method [46]. Starting from commercial 3-amino-rhodanine **1d**, compounds **1f**–**i** were synthesized easily, according to a recent published procedure developed in our laboratory [47].

With the aim to develop a more efficient synthetic process for the synthesis of highly functionalized 5-arylidene rhodanines **3a**–**v** from the building blocks **1a**–**i** and **2a**–**p**, microwave irradiation was employed to shorten the reaction times in comparison to conventional heating. For the reported methods, the Knoevenagel condensations have been realized in the presence of catalyst with various solvents: sodium acetate in glacial acetic acid [48] or in ethanol [46], piperidinium benzoate in toluene [49], tetrabutylammonium bromide in water [50], and piperidine in ethanol [51], using conventional heating in an oil bath. These older protocols suffer from one or more limitations, such as requiring harsh reaction conditions, low moderate yields, cumbersome experimental process, and long reaction time. Considering the latter aspect, we reasoned that microwave dielectric heating assisted chemistry might be an interesting approach for the synthesis of 5-arylidene rhodanine derivatives as potential biological molecules.

For this study, we investigated the use of three microwave reactors: the Synthewave^®^ 402 and the Explorer^®^ 24 apparatus with continuous irradiation power from 0 to 300 W and the Monowave^®^ 300 with continuous power from 0 to 800 W. These three microwave irradiation reactors comprise a mono-mode microwave cavity that operates at a frequency of 2.45 GHz; the reaction temperature in the microwave cavity was controlled with a calibrated infrared sensor, and the microwave irradiation parameters (power and temperature) were monitored by a software package. For optimization of the experimental reaction conditions (selection of the appropriate reactor, reaction temperature and irradiation power), we have not realized a comparative study between these three reactors because we focused only our work on the microwave apparatus, which lead us to better results.

As can be seen in Table 1, the experiments revealed that the optimal reaction conditions for the 5-arylidene-2-thioxo-1,3-thiazolidin-4-ones **3a**–**l** were obtained in an open vessel after 60 min at 80 °C (with a power of 90 W) in the Synthewave^®^ 402 apparatus with a stoichiometry of 1/1–4 of aryl aldehyde **2** and the volatile propylamine in order to be able to develop one-pot tandem reactions (aldimine synthesis/Knoevenagel condensation) [52]. According to this microwave method, the compounds **3a**–**l** were synthesized in yields ranging from 62 to 89%. For the other compounds, **3m**, **3n**, and **3p**–**v,** we screened a range of another reaction condition parameters in a closed vessel (a tube sealed with a snap cap) with the appropriate microwave reactors. With the conditions shown in Table 1, AcONa (0.1 or 1–1.2 eq) and 0.1 eq of piperidine in glacial acetic acid (0.1–6.6 eq) were identified to execute the Knoevenagel reactions in good yields (62–98% excepted for **3p**). Unfortunately, we are not able to use this protocol for the preparation of compound **2o**. It was obtained in moderate yield (**2o**: 26%) using classical heating in an oil bath. The structures of the desired 5-arylidene-2-thioxo-1,3-thiazolidin-4-ones **3a**–**v** were substantiated by ^1^H, ^13^C NMR (Supplementary Materials), and HRMS analyses, and only the thermodynamically more stable *Z*-isomers were obtained [50].

#### 2.1.2. Synthesis of (5*Z*) 2-Amino-5-arylidene-1,3-thiazolidin-4-one Library

With the compounds **3a**–**v** in hand, we investigated the preparation of novel 2-amino-5-arylidene-1,3-thiazolidin-4-ones **5**, according to Scheme 1. Transformation of 5-arylidene-rhodanine derivatives **3** into their 2-amino-5-arylidene-1,3-thiazolidin-4-ones **5** involved a sulfur/nitrogen displacement in the presence of a primary amine [53] or a secondary amine [54]. Commonly, the synthesis of 2-amino-1,3-thiazol-4-one involved activation of the C=S bond of the starting 5-arylidene rhodanine, and reaction with an halogenoalcane (i.e., EtI or MeI) potentially gave the desired *S*-alkyl-5-arylidene-1,3-thiazol-4(5*H*)-one, together with the *N*-alkyl-5-arylidene rhodanine derivative, as a side product [55]. In this context, we preferred to directly investigate the sulfur/nitrogen displacement in a faster and more efficient way by using microwave dielectric heating for the synthesis of targeted compounds **5** from the intermediate **3** and a variety of polar cyclic secondary amines **4**. In order to obtain interesting information for the structure-activity relationship (SAR) study on these compounds **5** as inhibitors of the protein kinase DYRK-1A, compounds **5** were designed to evaluate how the electrostatic and steric nature of the substituents on the phenyl ring and/or the presence of various amino moieties in position C-2 will influence the biological activity. For this study, we have employed eight polar cyclic secondary amines **4a**–**h** as elements of the second point of molecular diversity for the construction of compounds **5**. After a preliminary screening of the microwave irradiation conditions (open or closed vessel mode, temperature, power of irradiation, time), the experiments revealed the optimal ratio of **3**/**4** under microwave irradiation was obtained with 1.5 eq of amino reagent **5** using the “open vessel mode”. For the other parameters (temperature: 80–100 °C, power: 80–150 W, time: 20–60 min), the results for each product **5** are presented in Table 2. To verify the versatility and efficiency of this open vessel microwave protocol, different starting 5-arylidene rhodanine derivatives **3** and commercially available cyclic secondary amines **4a**–**h** were used as building blocks to generate a collection of seventeen new 2-amino-5-arylidene-1,3-thiazolidin-4-ones **5a**–**q**: in all cases, the products were easily isolated in good to high yields (72–96%) after a simple precipitation from EtOH. The structure identification of all these new compounds **5** were based on the ^1^H and ^13^C assignments and was performed extensive 1D and 2D NMR spectroscopy. For the exocyclic double bond (CH=C) of all the 2-amino-5-arylidene-1,3-thiazolidin-4-ones **5**, ^1^H NMR spectra show only one signal for the methylene proton (CH=) in the range of 7.56–7.61 ppm at lower field values than those expected or previously reported in the literature for the *E*-isomers which, strongly indicates that the compounds **5a**–**q** have the *Z*-configuration because of the high degree of thermodynamic stability of this isomer [56].

**Scheme 2:**
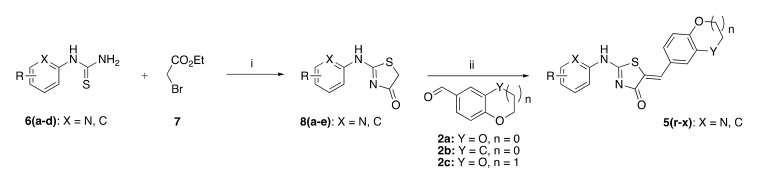
General synthetic approach for the (5*Z*) 2-*N*-heteroarylamino-5-arylidene-1,3-thiazol-4(5*H*)-one derivatives **5r**–**x** from the starting 2-*N*-heteroarylamino-1,3-thiazolidin-4-one **8a**–**e** and aromatic aldehydes **3**(**a**–**c**). Reagents and reaction conditions: (i) **7** 1.3 eq, EtOH, 55 °C, 3.5–22 h; (ii) piperidine 0.1 eq or AcONa 0.1 eq, AcOH 0.1–6.6 eq, 120–150 °C, MWI in closed vessel (Explorer 24^®^ CEM μWave reactor), 20–40 min.

To extend the molecular diversity on the C-2 position of the rhodanine platform, we have also examined introduction of aromatic primary amine (i.e., aniline, C-substituted aniline, amino pyridine, etc.) using the microwave dielectric heating protocol for sulfur/nitrogen displacement from 5-arylidene-2-thioxo-1,3-thiazolidin-4-one **3** and appropriate aromatic primary amine. After screening of the microwave irradiation conditions based on the reaction temperature (90–160 °C), the power for irradiation (90–150 W), the reaction time (30–60 min), the use of polar or non-polar solvent (EtOH, hexane), the use of open or closed vessel mode, and the ratio of the reagents (ratio **3**/aromatic amine: 1–3.5), it was not possible to obtain the desired 2-*N*-arylamino-5-arylidene-1,3-thiazolidin-4-one **5** after analysis of the crude reaction mixture by ^1^H NMR. Accordingly, we decided to drop this synthetic strategy for access to 2-*N*-arylamino-5-arylidene-1,3-thiazolidin-4-one and to focus now on the use of 2-*N*-heteroarylamino-1,3-thiazolidin-4-ones **8a**–**e** in Knoevenagel condensation (Scheme 2). The designed compounds **8a**–**e** were easily prepared from *N*-aryl thiourea **6a**–**d** and ethyl bromoacetate according to a protocol developed in our laboratory [55]. As shown in Table 3, the “closed vessel” microwave irradiation procedure involved the use of piperidine (0.1 eq) or AcONa (0.1–1 eq) in glacial acetic acid for the synthesis of 2-*N*-heteroarylamino-5-arylidene-1,3-thiazolidin-4-one **5r**–**x** from the building blocks **8a**–**e** and aromatic aldehydes **2a**–**c** after 20–40 min at 120 or 150 °C with a power of 100–200 W. This protocol is also very simple to execute because the desired products **5r**–**x** were isolated after crystallization from ethanol in moderate to good yields (34–90%). The expected *Z*-geometry of these compounds **5** was confirmed by the chemical shift of the exocyclic methylene proton (CH=) obtained in ^1^H NMR [56].

### 2.2. Biological Activities

The twenty-two (5*Z*) 5-arylidene-2-thioxo-1,3-thiazolidin-4-ones **3a**–**v** and the twenty-four (5*Z*) 2-amino-5-arylidene-1,3-thiazolidin-4(5*H*)-ones **5a**–**x** were evaluated against four different and representative in vitro kinase assays DYRK1A (dual specificity tyrosine phosphorylation regulated kinase), CK1 δ/e (caseine kinase 1), CDK5/p25 (cyclin dependant kinase) [57], and GSK3 α/β (glycogen synthase kinase 3) to evaluate their inhibition potency. These kinases were purified and assayed in the presence of 15 μM ATP and appropriate substrates (GS-1 peptide for GSK3 α/β, CKS peptide for CK1 δ/ε, histone H1 for CDK5/p25) as previously described [39]. All the compounds were initially tested at a 10 μM concentration. Those displaying less than 50% inhibition were considered inactive. Compounds showing more than 50% inhibition at 10 μM were next evaluated over a wide range of concentrations (usually 0.01, 5 and 10 μM), and the IC_50_ values were calculated from the dose-responses curves (Sigma–plot). (*R*)-Roscovitine and harmine (a natural β-carboline alkaloid known to be a potent inhibitor of DYRK1A [12]) were also used as positive control, and its IC_50_ values were compared with those obtained for the compounds **3** and **5**. Results are summarized, respectively, in Table 4 for the (5*Z*) 5-arylidene-2-thioxo-1,3-thiazolidin-4-one **3a**–**v**, and Table 5 for the (5*Z*) 2-amino-5-arylidene-1,3-thiazol-4(5*H*)-one **5a**–**x**.

Results given in Table 4 showed that, among these products **3**, the molecules **3j**–**l** and **3n**–**t** were completely inactive on the four kinases, DYRK1A, CK1, CDK5/p25, and GSK3, for which IC_50_ values > 10 μM were observed. Some interesting results were obtained for products **3h**,**i**, **3m**, and **3u**,**v** with micromolar IC_50_ values for DYRK1A. Among these compounds, the presence of the aminoacetic moiety on *N*-3 position of **3u** leads to enhancement of the inhibitory kinase activity for DYRK1A (**3u**: IC_50_ 0.16 μM). The most promising results were obtained with products **3a**, **3c**, and **3e**–**g** in this series, which exhibited selective, and sub-micromolar inhibitory activity toward DYRK1A (0.090 μM > IC_50_ > 0.028 μM). The molecule **3c** bearing a hydroxyl group at the C-4 position of the exocyclic phenyl ring was strongly considered as the most interesting compound with an IC_50_ value of 0.028 μM. In compound **3j**, the presence of a supplementary hydroxyl group in the C-3 position of the phenyl ring completely decreases inhibition of kinase activity (**3j**: IC_50_ > 10 μM), which confirms that the presence of only one hydroxyl group on the phenyl ring of **3c** seems to be crucial for an optimal inhibition activity. In product **3e**, a small methoxy group was partially tolerated, but the DYRK1A inhibition activity decreases slightly (**3c**: IC_50_ 0.064 μM). The introduction of more bulky groups at the 5-ylidene position as 1,3-benzodioxol-5-yl in **3a** (IC_50_ 0.070 μM) or 2,3-dihydro-1,4-benzodioxin-5-yl in **3f** (IC_50_ 0.056 μM) or 2,3-dihydro-benzofuran-5-yl in **3g** (IC_50_ 0.090 μM) maintained partially the kinase inhibition activity.

Concerning the library of the twenty-four (5*Z*) 2-amino-5-arylidene-1,3-thiazolidin-4(5*H*)-one **5a**–**x** derivatives, the results are given in Table 5. The main part of their activity was focused on DYRK1A (and GSK3 α/β for some of them) for fourteen products in this second series (**5a**, **5e**–**g**, **5k**–**m**, **5p**–**r**, and **5u**–**x**), which exhibited inhibitory activity in the micromolar and sub-micromolar ranges (2.3 μM > IC_50_ > 0.12 μM). For the three compounds **5i**, **5n**, and **5o**, we observed low nano-molar inhibition activity of DYRK1A (IC_50_ values for **5i**: 0.053 μM, **5n**: 0.070 μM, and **5o**: 0.090 μM) with moderate selectivity because they are also able to inhibit GSK3 α/β. It is interesting to note that these three compounds **5i**, **5n**, and **5o** are, respectively, substituted with bulky groups at the 5-ylidene position (1,3-benzodioxol-5-yl or 2,3-dihydro-1,4-benzodioxin-5-yl) and piperidin-1-yl or thiomorpholin-1-yl moieties in the C-2 position of the 1,3-thiazolidin-4(5*H*)-one platform. Finally, introducing a pyridin-2-yl group in *N*-2 position of the molecule **5s** resulted in selective and sub-micromolar affinity for DYRK1A (**5s**: IC_50_ 0.033 μM). Replacement of the pyridin-2-yl group in *N*-2 position by a pyridin-4-yl at the same position in compound **5t** resulted in a complete loss of kinase inhibitory activity on DYRK1A (**5t**: IC_50_ > 10 μM). Again, the replacement of the pyridin-2-yl or pyridin-4-yl moiety in *N*-2 position by a phenyl group, as example in molecule **5r**, maintained a moderate inhibition activity at a micromolar range (**5r**: IC_50_ 0.61 μM).

Comparison of all the results obtained in the two series showed the difficulties to define any role for the various substituents on the exocyclic phenyl ring (arylidene moiety in C-5 position), as well on the amino substituents in *N*-2 position, in order to obtain sub-micromolar affinity for DYRK1A with good selectivity. However, it is interesting to note that the presence of hydroxyl group in *para*–position of the phenyl ring of 5-arylidene-2-thioxo-1,3-thiazolidin-4-one derivative **3c** is very important to access of a sub-micromolar inhibitory activity for DYRK1A. However, the presence of the bulky 1,3-benzodioxol-5-yl group on the 5-arylidene moiety is not a crippling factor when it is associated with a thiomorpholin-1-yl substituent in *N*-2 position, with is the case for the compound **5n** (IC_50_ 0.070 μM).

To evaluate the potency of selected compounds **3** and **5** for their in vitro inhibition of cell proliferation, we used a panel of 6 representative tumoral cell lines, namely Huh7 (differential hepatocellular carcinoma), Caco2 (differentiating colorectal adenocarcinoma), HCT (actively proliferating colorectal carcinoma), PC3 (prostate carcinoma), NCI-H2 (lung carcinoma), MDA-MB 231 (breast carcinoma), and diploid skin fibroblasts, as normal cell lines for control. Results of the in vitro antiproliferative data activity are reported in Table 6. The most active compound is clearly the (5*Z*)-5-(2,3-dihydro-benzofuran-5-ylmethylene)-2-thioxo-1,3-thiazolidin-4-one **3g**, which exhibits antitumor activities in the Caco2 and HCT 116 cell lines with IC_50_ values lower than 10 μM (Caco2: IC_50_ 8 μM and HCT 116: IC_50_ 6 μM). Compound **3g** also presents a selective and sub-micromolar inhibitory activity on DYRK1A (IC_50_ 0.090 μM). It is interesting to note that **3c** is also moderately active in the HCT 116 cell line (IC_50_ 12 μM) but showed a more efficient inhibitory activity for DYRK1A (IC_50_ 0.064 μM). Compounds **3c** and **3g** also did not exhibit the growth of normal fibroblasts (IC_50_ > 25 μM). In the selected (5*Z*) 2amino-5-arylidene-1,3-thiazol-4(5*H*)-one derivatives **5**, none of these compounds presented a significant activity against the six representative tumoral cell lines.

## 3. Material and Methods

### 3.1. Chemistry–General Remarks

Melting points were determined on a Kofler melting point apparatus and were uncorrected. ^1^H NMR spectra were recorded on BRUKER AC 300 P (300 MHz) spectrometer, ^13^C NMR spectra on BRUKER AC 300 P (75 MHz) spectrometer. Chemical shifts are expressed in parts per million downfield from tetramethylsilane as an internal standard. Data are given in the following order: *δ* value, multiplicity (s, singlet; d, doublet; t, triplet; q, quartet; m, multiplet; br, broad), and number of protons, and coupling constants *J* is given in Hertz. The mass spectra (HRMS) were taken, respectively, on a MS/MS ZABSpec Tof Micromass (EBE TOF geometry) at an ionizing potential of 8 eV and on a VARIAN MAT 311 at an ionizing potential of 70 eV in the “Centre Régional de Mesures Physiques de l’Ouest” (CRMPO, Rennes, France). Reactions under microwave irradiations were realized in the Synthewave^®^ 402 apparatus (Merck Eurolab, Div. Prolabo, France) or in the Explorer^®^ 24 CEM microwave reactor (CEM France, Saclay, France), as well as in the Anton Paar Monowave 300^®^ microwave reactor (Anton Paar France, Les Ulis, France). Microwave irradiation reactions were realized in open cylindrical quartz reactor (Ø = 1.8 cm) with the Synthewave^®^ 402 apparatus or in borosilicate glass vials of 10 mL equipped with snap caps (at the end of the irradiation, cooling reaction was realized by compressed air) with the Explorer^®^ 24 or Monowave^®^ 300 reactors. The microwave instrument consists of a continuous focused microwave power output from 0 to 300W for the Synthewave^®^ 402 or the Explorer^®^ 24 CEM apparatus and from 0 to 800W for the Anton Paar Monowave 300^®^ apparatus. All the experiments in each microwave reactor were performed using the stirring option. The target temperature was reached with a ramp of 2–5 min, and the chosen microwave power stays constant to hold the mixture at this temperature. The reaction temperature is monitored using calibrated infrared sensor and the reaction time included the ramp period. The microwave irradiation parameters (power and temperature) were monitored by the ChemDriver software package for the Explorer^®^ 24 CEM apparatus and by the Monowave software package for the Anton Paar Monowave 300^®^ reactor. Solvents were evaporated with a BUCHI rotary evaporator. All reagents and solvents were purchased from Acros (Geel, Belgium), Sigma-Aldrich Chimie (Saint-Quentin-Fallavier, France), and Fluka France and were used without further purification.

#### 3.1.1. Standard Procedure for the Preparation of 5-Arylidene-2-thioxo-1,3-thiazolidin-4-one (**3a**–**l**) in the Synthewave^®^ 402 Microwave Reactor

In a cylindrical quartz reactor (Ø = 1.8 cm), successively commercial rhodanine **1a** (1 eq), aromatic aldehyde **2** (1 eq), and *n*-propylamine (2 eq) were placed. The reactor was then introduced into the Synthewave^®^ 402 Prolabo microwave cavity (power: 300 W). The stirred mixture was irradiated at 80 °C (after a ramp of 2 min. from 20 to 80 °C) for 60 min (power level: 30%, 90 W). After microwave dielectric heating, the crude reaction mixture was allowed to cooling down at room temperature, and ethanol (20 mL) was added in the cylindrical quartz reactor. The resulting insoluble product **3** was filtered, recrystallized in EtOH, and dried under high vacuum (10^−2^ Torr) for 1 h.

*(5Z)-5-(1,3-Benzodioxol-5-ylmethylene)-2-thioxo-1,3-thiazolidin-4-one* (**3a**). According to the standard procedure, **3a** was synthesized from commercial rhodanine **1a** (958 mg, 7.2 mmol), piperonaldehyde **2a** (1.081 g, 7.2 mmol), and *n*-propylamine (1.17 mL, 839 mg, 14.2 mmol) in 79% yield as light-yellow powder; mp = 246–250 °C. ^1^H NMR (DMSO-*d_6_*) δ: 6.13 (s, 2H, OCH_2_O); 7.11 (d, 3H, *J =* 8.7 Hz, H-2, H-5, H-6, Ar); 7.54 (s, 1H, =CH); 13.74 (br s, 1H, NH). ^13^C NMR (DMSO-*d_6_*) δ: 102.1 (OCH_2_O); 109.2 (C-2′); 109.4 (C-5′); 122.8 (C=, C-5); 126.6 (C-6′); 127.1 (CH=); 131.8 (C-7′); 148.2 (C-4′); 149.6 (C-3′); 169.3 (C=O, C-4); 195.3 (C=S, C-2). HRMS, *m*/*z:* 264.9864 found (calculated for C_11_H_7_NO_3_S_2_, M^+.^ requires 264.9867).

*(5Z)-5-(3-Hydroxy-4-methoxybenzylidene)-2-thioxo-1,3-thiazolidin-4-one* (**3b**). According to the standard procedure, **3b** was synthesized from commercial rhodanine **1a** (958 mg, 7.2 mmol), 3-hydroxy-4-methoxybenzaldehyde **2d** (1.095 g, 7.2 mmol), and *n*-propylamine (1.17 mL, 839 mg, 14.2 mmol) in 81% yield as light-yellow powder; mp = 222–224 °C. ^1^H NMR (DMSO-*d_6_*) δ: 3.82 (s, 3H, OCH_3_); 6.94 (d, 1H, *J =* 6 Hz, H-6, Ar); 7.04 (d, 1H, *J =* 7.9 Hz, H-5, Ar); 7.11 (s, 1H, H-2, Ar); 7.53 (s, 1H, =CH); 10.06 (br s, 1H, OH); 13.65 (br s, 1H, NH). ^13^C NMR (DMSO-*d_6_*) δ: 56.0 (OCH_3_); 114.7 (C-2′); 116.7 (C-5′); 121.5 (C=, C-5); 124.8 (C-6′); 125.5 (C-1′); 133.1 (=CH); 148.5 (C-4′); 150.4 (C-3′); 169.8 (C=O, C-4); 195.8 (C=S, C-2). HRMS, *m*/*z:* 267.0023 found (calculated for C_11_H_9_NO_3_S_2_, M^+.^ requires 267.0024).

*(5Z)-5-(4-Hydroxy-3-methoxybenzylidene)-2-thioxo-1,3-thiazolidin-4-one* (**3c**). According to the standard procedure, **3c** was synthesized from commercial rhodanine **1a** (665 mg, 5 mmol), 4-hydroxy-3-methoxybenzaldehyde **2e** (1.52 g, 10 mmol), and *n*-propylamine (1.64 mL, 1.18 g, 20 mmol) in 80% yield as light-yellow powder; mp = 220–222 °C. ^1^H NMR (DMSO-*d_6_*) δ: 3.82 (s, 3H, OCH_3_); 7.00 (d, 1H, *J =* 8.9 Hz, H-6, Ar); 7.07 (d, 1H, *J =* 8.6 Hz, H-5, Ar); 7.13 (s, H-2, Ar); 7.48 (s, 1H, =CH); 9.57 (br s, 1H, OH); 13.62 (br s, 1H, NH). ^13^C NMR (DMSO-*d_6_*) δ: 56.1 (OCH_3_); 112.9 (C-2′); 116.5 (C-5′); 122.4 (C=, C-5); 124.8 (C-6′); 126.1 (C-1′); 132.7 (=CH); 147.5 (C-4′); 150.9 (C-3′); 169.9 (C=O, C-4); 196.0 (C=S, C-2). HRMS, *m*/*z:* 267.0022 found (calculated for C_11_H_9_NO_3_S_2_, M^+.^ requires 267.0024).

*(5Z)-5-(3,4-Dimethoxybenzylidene)-2-thioxo-1,3-thiazolidin-4-one* (**3d**). According to the standard procedure, **3d** was synthesized from commercial rhodanine **1a** (958 mg, 7.2 mmol), 3,4-dimethoxybenzaldehyde **2h** (1.19 g, 7.2 mmol), and *n*-propylamine (1.18 mL, 851 mg, 14.4 mmol) in 83% yield as light-yellow powder; mp = 232–234 °C. ^1^H NMR (DMSO-*d_6_*) δ: 3.74 (s, 3H, OCH_3_); 3.81 (s, 3H, OCH_3_); 7.14 (s, 1H, H-2, Ar); 7.32 (d, 1H, *J* = 8.6 Hz, H-5, Ar); 7.44 (d, 1H, *J* = 6.3 Hz, H-6, Ar); 7.59 (s, 1H, =CH); 13.72 (br s, 1H, NH). ^13^C NMR (DMSO-*d_6_*) δ: 57.0 (OCH_3_); 57.1 (OCH_3_); 112.4 (C-2′); 112.7 (C-5′); 123.6 (C=, C-5); 124.8 (C-6′); 127.5 (C-1′); 128.7 (=CH); 150.4 (C-4′); 150.7 (C-3′); 169.5 (C=O, C-4); 195.8 (C=S, C-2). HRMS, *m*/*z:* 281.0167 found (calculated for C_12_H_11_NO_3_S_2_, M^+.^ requires 281.0184).

*(5Z)-5-(4-Hydroxybenzylidene)-2-thioxo-1,3-thiazolidin-4-one* (**3e**). According to the standard procedure, **3e** was synthesized from commercial rhodanine **1a** (1.33 g, 10 mmol), 4-hydroxybenzaldehyde **2i** (1.22 g, 10 mmol), and *n*-propylamine (1.64 mL, 1.18 g, 20 mmol) in 80% yield as light-yellow powder; mp = 240–242 °C. ^1^H NMR (DMSO-*d_6_*) δ: 6.91 (d, 2H, *J* = 8.6 Hz, H-3, Ar); 7.47 (d, 2H, *J =* 8.6 Hz, H-2, Ar); 7.58 (S, 1H, =CH); 10.48 (br s, 1H, OH); 13.68 (br s, 1H, NH). ^13^C NMR (DMSO-*d_6_*) δ: 116.7 (C-3′); 123.9 (C-2′); 123.8 (C=, C-5); 129.1 (C-1′); 130.7 (=CH); 159.9 (C-4′); 169.7 (C=O, C-4); 195.7 (C=S, C-2). HRMS, *m*/*z:* 236.9918 found (calculated for C_10_H_7_NO_2_S_2_, M^+.^ requires 236.99182).

*(5Z)-5-(2,3-Dihydro-1,4-benzodioxin-6-ylmethylene)-2-thioxo-1,3-thiazolidin-4-one* (**3f**). According to the standard procedure, **3f** was synthesized from commercial rhodanine **1a** (958 mg, 7.2 mmol), 2,3-dihydro-1,4-benzodioxin-6-carboxaldehyde **2c** (1.18 g, 7.2 mmol), and *n*-propylamine (1.18 mL, 851 mg, 14.4 mmol) in 89% yield as light-yellow powder; mp = 250–252 °C. ^1^H NMR (DMSO-*d_6_*) δ: 4.29 (t, 2H, OCH_2_CH_2_O); 4.30 (t, 2H, OCH_2_CH_2_O); 7.02 (d, 1H, *J* = 8.1 Hz, H-5, Ar); 7.08 (s, 1H, H-2, Ar); 7.10 (d, 1H, *J* = 8.2 Hz; H-6, Ar); 7.53 (s, 1H, =CH); 13.76 (br s, 1H, NH). ^13^C NMR (DMSO-*d_6_*) δ: 63.8 (OCH_2_CH_2_O); 64.4 (OCH_2_CH_2_O); 118.6 (C-5′); 119.7 (C-2′); 123.5 (=CH); 124.7 (C-6′); 126.7 (C-1′); 132.1 (C=, C-5); 144.2 (C-4′); 146.5 (C-3′); 170.0 (C=O, C-4); 195.9 (C=S, C-2). HRMS, *m*/*z:* 279.0016 found (calculated for C_12_H_9_NO_3_S_2_, M^+.^ requires 279.0024).

*(5Z)-5-(2,3-Dihydro-benzofuran-5-ylmethylene)-2-thioxo-1,3-thiazolidin-4-one* (**3g**). According to the standard procedure, **3g** was synthesized from commercial rhodanine **1a** (1.357 g, 10.2 mmol), 2,3-dihydro-1-benzofuran-5-carbaldehyde **2b** (1.067 mg, 7.2 mmol), and *n*-propylamine (1.67 mL, 1.205 g, 20.4 mmol) in 79% yield as light-yellow powder; mp = 250–252 °C. ^1^H NMR (DMSO-*d_6_*) δ: 4.02 (t, 2H, *J* = 6.8 Hz; CH_2_CH_2_O); 6.02 (t, 2H, *J* = 6.9 Hz; CH_2_CH_2_O); 6.84 (d, 1H, *J* = 8 Hz, H-3, Ar); 7.12 (dd, 1H, *J* = 8, 1.5 Hz, H-2, Ar); 7.37 (d, 1H, *J* = 1.4 Hz, H-6, Ar); 8.16 (s, 1H, =CH); 13.66 (br s, 1H, NH). ^13^C NMR (DMSO-*d_6_*) δ: 29.2 (CH_2_CH_2_O); 71.7 (CH_2_CH_2_O); 107.0 (C-5′); 108.4 (C-2′); 123.5 (=CH); 124.7 (C-6′); 126.7 (C-1′); 132.1 (C=, C-5); 144.2 (C-4′); 148.6 (C-3′); 170.0 (C=O, C-4); 195.9 (C=S, C-2). HRMS, *m*/*z:* 263.0063 found (calculated for C_12_H_9_NO_2_S_2_, M^+.^ requires 263.0075).

*(5Z)-5-{[6-(1,3-Benzodioxol-5-yl)pyridin-2-yl]methylene}-2-thioxo-1,3-thiazolidin-4-one* (**3h**). According to the standard procedure, **3h** was synthesized from commercial rhodanine **1a** (18 mg, 0.134 mmol), 6-(1,3-benzodioxol-5-yl)-2-pyridinecarbaldehyde **2k** (30 mg, 0.134 mmol), and *n*-propylamine (22 mL, 15.8 mg, 0.268 mmol) in 79% yield as light-yellow powder; mp = 246–250 °C. ^1^H NMR (DMSO-*d_6_*) δ: 6.05 (s, 2H, OCH_2_O); 6.94 (d, 1H, *J* = 8 Hz, H-3′); 7.55 (dd, 1H, *J* = 8, 1.5 Hz, H-2′); 7.59 (d, 1H, *J* = 8 Hz, H-3); 7.66 (dd, 1H, *J* = 8, 1.5 Hz, H-5); 7.77 (t, 1H, *J* = 8 Hz, H-4); 7.93 (dd, 1H, *J* = 1.4 Hz, H-6′); 8.46 (s, 1H, =CH); 12.46 (br s, 1H, NH). ^13^C NMR (DMSO-*d_6_*) δ: 101.8 (OCH_2_O); 107.0 (C-5″); 107.5 (C-6′); 108.4 (C-2″); 114.0 (C-4′); 116.0 (C=, C-5); 118.0 (C-5″); 124.5 (C-6″); 13.,6 (C-1″); 131.9 (=CH); 132.1 (C-3″); 132.4 (C-2′); 148.6 (C-3″); 150.0 (C-4″); 169.9 (C=O, C-4); 192.4 (C=S, C-2). HRMS, *m*/*z:* 342.0131 found (calculated for C_16_H_10_N_2_O_3_S_2_, M^+.^ requires 342.0133).

*(5Z)-5-[(7-Bromo-1,3-benzodioxol-5-yl)methylene]-2-thioxo-1,3-thiazolidin-4-one* (**3i**). According to the standard procedure, **3i** was synthesized from commercial rhodanine **1a** (1.31 g, 9.8 mmol), 7-bromo-1,3-benzodioxole-5-carbaldehyde **2l** (2.24 g, 9.8 mmol), and *n*-propylamine (1.6 mL, 1.15 g, 19.6 mmol) in 70% yield as light-yellow powder*;* mp > 260 °C. ^1^H NMR (DMSO-*d_6_*) δ: 6.18 (s, 2H, OCH_2_O); 6.37 (s, 1H, H-6); 7.39 (s, 1H, H-2); 7.88 (s, 1H, =CH); 12.10 (br s, 1H, NH). ^13^C NMR (DMSO-*d_6_*) δ: 100.7 (C-Br); 102.7 (OCH_2_O); 109.4 (C-2′); 110.7 (=CH); 127.7 (C-6′); 128.1 (C-1′); 128.7 (C=, C-5); 146.8 (C-4′); 148.7 (C-3′); 166.4 (C=O, C-4); 192.6 (C=S, C-2). HRMS, *m*/*z:* 342.8923 found (calculated for C_11_H_16_^79^BrNO_3_S_2_, M^+.^ requires 342.8972).

*(5Z)*-*5-(3,4-Dihydroxybenzylidene)*-*2-thioxo-1,3-thiazolidin-4-one* (**3j**). According to the standard procedure, **3j** was synthesized from commercial rhodanine **1a** (1.385 g, 10.4 mmol), 3,4-dihydroxybenzaldehyde **2f** (1.44 g, 10.4 mmol), and *n*-propylamine (1.7 mL, 1.23 g, 20.8 mmol) in 79% yield as light-yellow powder; mp = 210–212 °C. ^1^H NMR (DMSO-*d_6_*) δ: 6.82 (s, 1H, H-2); 6.89 (d, 1H, *J* = 8.6 Hz, H-6); 7.05 (d, 1H, *J* = 8.2 Hz, H-5); 7.47 (s, 1H, =CH); 9.54 (br s, 1H, OH); 9.96 (br s, 1H, OH); 13.66 (br s, 1H, NH). ^13^C NMR (DMSO-*d_6_*) δ: 115.5 (C-2′); 117.5 (C-5′); 123.2 (C=, C-5); 128.51 (C-6′); 129.8 (C-1′); 132.7 (=CH); 148.7 (C-4′); 149.4 (C-3′); 169.6 (C=O, C-4); 195.8 (C=S, C-2). HRMS, *m*/*z:* 252.9862 found (calculated for C_10_H_7_NO_3_S_2_, M^+.^ requires 252.9867).

*(5Z)*-*5-(3,5-Dihydroxybenzylidene)*-*2-thioxo-1,3-thiazolidin-4-one* (**3k**). According to the standard procedure, **3k** was synthesized from commercial rhodanine **1a** (1.385 g, 10.4 mmol), 3,5-dihydroxybenzaldehyde **2n** (1.44 g, 10.4 mmol), and *n*-propylamine (1.7 mL, 1.23 g, 20.8 mmol) in 79% yield as light-yellow powder; mp = 230–232 °C. ^1^H NMR (DMSO-*d_6_*) δ: 6.62 (s, 1H, H-3); 6.65 (d, 1H, *J* = 8.7 Hz, H-5); 7.46 (d, 1H, *J* = 5.8 Hz, H-6); 7.94 (s, 1H, =CH); 10.23 (br s, 1H, OH); 10.54 (br s, 1H, OH); 13.72 (br s, 1H, NH). ^13^C NMR (DMSO-*d_6_*) δ: 104.9 (C-3′); 110.1 (C-5′); 114.1 (C-6′); 122.4 (C=, C-5); 130.5 (C-1′); 130.8 (=CH); 159.8 (C-2′); 161.1 (C-4′); 169.9 (C=O, C-4); 196.1 (C=S, C-2). HRMS, *m*/*z:* 252.9859 found (calculated for C_10_H_7_NO_3_S_2_, M^+.^ requires 252.9867).

*(5Z)*-*5-(1-Benzofuran-2-ylmethylene)*-*2-thioxo-1,3-thiazolidin-4-one* (**3l**). According to the standard procedure, **3l** was synthesized from commercial rhodanine **1a** (1.33 g, 10 mmol), 1-benzofuran-2-carbaldehyde **2m** (1.52 g, 10.4 mmol), and *n*-propylamine (1.64 mL, 1.18 g, 20 mmol) in 62% yield as light-yellow powder; mp = 246–250 °C. ^1^H NMR (DMSO-*d_6_*) δ: 6.98 (s, 1H, C-3); 7.11–7.28 (m, 2H, H-5, H-6); 7.47 (d, 1H, *J* = 7.7 Hz, H-7); 7.83 (d, 1H, *J* = 7.5 Hz, H-4); 8.51 (s, 1H, H-2); 8.98 (s, 1H, =CH); 12.10 (br s, 1H, NH). ^13^C NMR (DMSO-*d_6_*) δ: 107.2 (=CH); 108.8 (C-3′); 112.6 (C-7′); 118.6 (C-4′); 121.2 (C-5′); 122.1 (C=, C-5); 123.1 (C-6′); 127.6 (C-3a′); 129.7 (C-2′); 136.4 (C-7a′); 164.3 (C=O, C-4); 194.1 (C=S, C-2). HRMS, *m*/*z:* 260.9842 found (calculated for C_12_H_7_NO_2_S_2_, M^+.^ requires 260.9918).

*7-Bromo-1,3-benzodioxole-5-carbaldehyde* (**2l**). In a 25 mL round-bottomed flask provided with a magnetic stirrer and condenser, a mixture of commercial 3-bromo-4,5-dihydroxybenzaldehyde (217 mg, 1 mmol), potassium fluoride (529 mg, 9.1 mmol, 9.1 eq), and dibromomethane (70 mL, 173 mg, 1 mmol) in 3 mL of dimethylformamide was stirred vigorously under a stream of nitrogen for 4 h at 140 °C in an oil bath. After cooling down to room temperature, 3 mL of deionized water was added in one portion to the crude reaction mixture. The resulting solution was submitted to extraction with Et_2_O (3 × 5 mL), and, after decantation, the organic layer was dried over MgSO_4_. After filtration, the solvent of the filtrate was eliminated in a rotary evaporator under reduced pressure; then, the crude residue was dried under high vacuum (10^−2^ Torr) at 25 °C for 2 h. The desired 7-bromo-1,3-benzodioxole-5-carbaldehyde **2l** was obtained as a light-yellow powder in 51% yield (117 mg) and was sufficiently pure to be used further without purification; mp = 123–125 °C. ^1^H NMR (DMSO-*d_6_*) δ: 6.17 (s, 2H, OCH_2_O); 7.28 (d, 1H, *J* = 1.4 Hz, H-2, Ar); 7.55 (d, 1H, *J* = 1.3 Hz, H-6); 9.78 (s, 1H, CHO). ^13^C NMR (DMSO-*d_6_*) δ: 100.9 (C-Br); 102.6 (OCH_2_O); 106.2 (C-2); 131.0 (C-6); 132.8 (C-1); 148.9 (C-4); 151.2 (C-3); 189.1 (C=O). HRMS, *m*/*z:* 227.9426 found (calculated for C_8_H_5_O_3_^79^Br, M^+.^ requires 227.9422).

#### 3.1.2. Standard Procedure for the Preparation of 5-Arylidene-2-thioxo-1,3-thiazolidin-4-one (**3m**,**n**) in the Monowave^®^ 300 Microwave Reactor

In a 10 mL glass tube were placed successively commercial rhodanine **1a** (1 eq), aromatic aldehyde **2o**,**p** (1.1–1.2 eq), sodium acetate (26.4 mg, 0.31 mmol, 0.1 eq), and glacial acetic acid (1.2 eq). The glass tube was sealed with a snap cap and placed in the Monowave 300^®^ Anton-Paar microwave cavity (power = 850 W). The mixture was irradiated at 140 °C for 20–30 min under vigorous magnetic stirring. After microwave dielectric heating, the crude reaction mixture was allowed to cool down at room temperature. To this crude mixture, 4 mL of deionized water was added, and the resulting suspension was submitted to ultrasound in a Branson 1510 apparatus at 25 °C for 30 min. Then, the desired compound **3** was collected by filtration, and the crude solid was washed with 5 mL of deionized water and dried under high vacuum (10^−2^ Torr) at 25 °C for 2 h.

*(5Z)*-*5-(Chroman-6-yl)methylene-2-thioxo-1,3-thiazolidin-4-one* (**3m**). According to the standard procedure, **3m** was synthesized after a reaction time of 20 min from commercial rhodanine **1a** (200 mg, 1.5 mmol), 6-chromanecarbaldehyde **2o** (292 mg, 1.8 mmol), sodium acetate (147 mg, 1.8 mmol), and glacial acetic acid (0.6 mL, 108 mg, 1.8 mmol) in 93% yield as light-yellow powder; mp > 260 °C. ^1^H NMR (DMSO-*d_6_*) δ: 1.92 (m, 2H, CH_2_); 2.77 (t, 2H, *J =* 6.3 Hz, CH_2_); 4.19 (m, 2H, CH_2_); 6.83 (d, 1H, *J =* 8.4 Hz, Ar); 7.27 (m, 2H, Ar); 7.47 (s, 1H, CH=); 12.06 (br s, 1H, NH). ^13^C NMR (DMSO-*d_6_*) δ: 21.3 (CH_2_); 24.1 (CH_2_); 66.6 (CH_2_); 117.5 (=CH); 121.9 (Ar); 123.6 (C=, C-5); 124.9 (Ar); 130.2 (Ar); 131.9 (Ar); 132.8 (Ar); 157.0 (Ar); 169.7 (C=O, C-4); 195.6 (C=S, C-2). HRMS, *m*/*z:* 300.0129 found (calculated for C_15_H_11_NO_2_NaS_2_, [M+Na]^+.^ requires 300.0129).

*(5Z)*-*5-(3,4-Dihydro-2H-1,5-benzodioxepine-7-yl)methylene-2-thioxo-1,3-thiazolidin-4-one* (**3n**). According to the standard procedure, **3n** was synthesized after a reaction time of 20 min from commercial rhodanine **1a** (200 mg, 1.5 mmol), 3,4-dihydro-2*H*-1,5-benzodioxepine-7-carbaldehyde **2p** (321 mg, 1.8 mmol), sodium acetate (147 mg, 1.8 mmol), and glacial acetic acid (0.6 mL, 108 mg, 1.8 mmol) in 94% yield as light-yellow powder; mp = 220–222 °C. ^1^H NMR (DMSO-*d_6_*) δ: 2.13 (m, 2H, CH_2_); 4.20 (m, 4H, 2xCH_2_); 7.05 (m, 1H, Ar); 7.15 (m, 2H, Ar); 7.50 (s, 1H, CH=); 13.68 (br s, 1H, NH). ^13^C NMR (DMSO-*d_6_*) δ: 30.7 (CH_2_); 70.4 (CH_2_); 70.5 (CH_2_); 122.4 (CH=) 123.7 (Ar); 123.8 (C=, C-5); 125.9 (Ar); 128.1 (Ar); 131.0 (Ar); 150.9 (Ar); 153.0 (Ar); 169.3 (C=O, C-4); 195.4 (C=S, C-2). HRMS, *m*/*z:* 316.0079 found (calculated for C_18_H_13_NO_2_NaS_2_, [M+Na]^+.^ requires 316.0078).

*(5Z)-3-Amino-5-benzo[1,3]dioxol-5-ylmethylene-2-thioxo-thiazolidin-4-one* (**3o**). In a 25 mL round-bottomed flask provided with a magnetic stirrer and condenser, a mixture of commercial 3-amino rhodanine **1d** (197 mg, 1.33 mmol), piperonaldehyde **2a** (200 g, 1.33 mmol), triethylamine (19 mL, 13 mg, 0.13 mmol, 0.1 eq), and glacial acetic acid (8 μL, 8 mg, 0.13 mmol, 0.1 eq) in 4.3 mL of ethyl acetate was stirred vigorously under a stream of argon for 3.5 h at 85 °C in an oil bath. After cooling down to room temperature, the insoluble product of the orange reaction mixture was filtered, washed with absolute ethanol (2 × 3 mL), and was dried under high vacuum (10^−2^ Torr) for 1 h. The desired 3-amino-5-benzo[1,3]dioxol-5-ylmethylene-2-thioxo-thiazolidin-4-one **3o** was obtained as a light-yellow powder in 26% yield (97 mg); mp = 224–226 °C. ^1^H NMR (DMSO-*d_6_*) δ: 5.92 (s, 2H, NH_2_); 6.15 (s, 2H, H-1); 7.12 (d, 1H, *J =* 8.1Hz, H-7); 7.19–7.25 (m, 2H, H-3, H-4); 7.79 (s, 1H, H-5); 12.71 (br s, 1H, NH). ^13^C NMR (DMSO-*d_6_*) δ: 102.1 (C-1′); 109.3 (C-4′); 109.6 (C-3′); 117.5 (C=, C-5); 127.0 (C-6′); 127.2 (C-5′); 133.5 (CH=); 148.3 (C-2′); 149.9 (C-2′); 163.7 (C=O, C-4); 187.1 (C=S, C-2). HRMS, *m*/*z:* 302.9872 found (calculated for C_11_H_8_N_2_O_3_NaS_2_, [M+Na]^+.^ requires 302.9874).

#### 3.1.3. Standard Procedure for the Preparation of 5-Arylidene-2-thioxo-1,3-thiazolidin-4-one (**3p**,**q**) and (**3s**,**t**) in the Explorer^®^ 24 Microwave Reactor

In a 10 mL glass tube were placed successively the 3-*N*-amino rhodanine derivative **1** (1 eq), piperonaldehyde **2a** (1 eq), piperidine (0.1 eq) or sodium acetate (0.1 eq), and glacial acetic acid (0.1–6.6 eq). The glass tube was placed in the Explorer^®^ 24 CEM microwave cavity (power = 300 W). The mixture was irradiated at 120 or 150 °C (with a power of 100 or 200 W) for 20 min under vigorous magnetic stirring. After microwave dielectric heating, the insoluble compound **3** was separated from the crude reaction mixture by filtration. To the collected crude compound **3**, absolute ethanol or deionized water was added, and the resulting suspension was stirred vigorously under magnetic stirring for 18 h. The desired compound **3** was collected by filtration and was dried under high vacuum (10^−2^ Torr) at 25 °C for 1 h.

*(5Z)-N-(5-Benzo[1,3]dioxol-5-ylmethylene-4-oxo-2-thioxo-thiazolidin-3-yl)-benzamide* (**3p**). According to the standard procedure, **3p** was synthesized at 150 °C with a power of 200 W from *N*-(4-oxo-2-thioxo-thiazolidin-3-yl)-benzamide **1f** (222 mg, 1 mmol), piperonaldehyde **2a** (150 mg, 1 mmol), piperidine (10 μL, 9 mg, 0.1 mmol, 0.1 eq), and glacial acetic acid (6 μL, 6 mg, 0.1 mmol, 0.1 eq) in 27% yield as light-yellow powder; mp = 182–184 °C. ^1^H NMR (DMSO-*d_6_*) δ: 6.64 (s, 2H, H-1); 7.56 (d, *J =* 8.1 Hz, 1H, Ar); 7.66 (d, *J =* 1.7 Hz, 1H, Ar); 7.77 (dd, *J =* 1.3, 8.2 Hz, 1H, Ar); 8.03 (m, 2H, Ar); 8.29 (s, 1H, CH=); 8.50 (m, 1H, Ar); 8.53 (m, 1H, Ar); 12.03 (br s, 1H, H-6′). ^13^C NMR (DMSO-*d_6_*) δ: 102.3 (OCH_2_O); 109.4 (Ar); 109.80 (Ar); 116.4 (C=, C-5); 126.9 (C=6); 127.6 (Ar); 127.8 (Ar); 128.8 (Ar); 132.9 (Ar); 135.2 (CH=); 148.4 (C*_ipso_*, C*_ortho_*, Ar); 150.3 (C-2′); 163.4 (C=O); 164.5 (C=O, C-4); 190.3 (C=S, C-2). HRMS, *m*/*z:* 385.0307 found (calculated for C_18_H_13_N_2_O_4_S_2_, [M+H]^+.^ requires 385.0317).

*(5Z)-N-(5-Benzo[1,3]dioxol-5-ylmethylene-4-oxo-2-thioxo-thiazolidin-3-yl)-2-phenyl-acetamide* (**3q**). According to the standard procedure, **3q** was synthesized at 120 °C with a power of 100 W from *N*-(4-oxo-2-thioxo-thiazolidin-3-yl)-2-phenyl-acetamide **1h** (50 mg, 0.19 mmol), piperonaldehyde **2a** (28 mg, 0.19 mmol), sodium acetate (15 mg, 0.19 mmol, 0.1 eq), and glacial acetic acid (70 μL, 74 mg, 1.24 mmol, 6.6 eq) in 98% yield as light-yellow powder; mp = 216–218 °C. ^1^H NMR (DMSO-*d_6_*) δ: 3.71 (s, 2H, CH_2_Ph); 6.16 (s, 2H, OCH_2_O); 7.13 (d, 1H, *J =* 8.1 Hz, Ar); 7.21 (m, 1H, Ar); 7.25 (m, 1H, Ar); 7.28 (m, 1H, Ar); 7.32–7.34 (m, 4H, Ar); 7.84 (s, 1H, CH=); 11.44 (br s, 1H, NH). ^13^C NMR (DMSO-*d_6_*) δ: 39.5 (CH_2_); 102.3 (OCH_2_O); 109.4 (Ar); 109.7 (Ar); 116.6 (C=, C-5); 126.7 (Ar); 126.9 (C*_ipso_*, Ar); 127.4 (Ar); 128.3 (Ar); 129.1 (Ar); 134.6 (C*_ipso_*, Ar); 134.9 (Ar); 148.4 (C_Ar_-O); 150.2 (C_Ar_-O); 163.2 (C=O); 168.6 (C=O, C-4); 190.2 (C=S, C-2). HRMS, *m*/*z:* 421.0294 found (calculated for C_19_H_14_N_2_O_4_NaS_2_, [M+Na]^+.^ requires 421.0293).

*(5Z)-5-Benzo[1,3]dioxol-5-ylmethylene-3-[2-(4-methoxy-phenyl)-2-oxo-ethyl]-2-thioxo-thiazolidin-4-one* (**3s**). According to the standard procedure, **3s** was synthesized at 120 °C with a power of 100 W from 4-methoxy-*N*-(4-oxo-2-thioxo-thiazolidin-3-yl)-benzamide **1g** (50 mg, 0.18 mmol), piperonaldehyde **2a** (27 mg, 0.18 mmol), sodium acetate (15 mg, 0.18 mmol, 0.1 eq), and glacial acetic acid (70 μL, 70 mg, 1.17 mmol, 6.6 eq) in 83% yield (61 mg) as light-yellow powder; mp = 226–228 °C. ^1^H NMR (DMSO-*d_6_*) δ: 3.85 (s, 3H, MeO); 6.18 (s, 2H, OCH_2_O); 7.11 (m, 2H, Ar); 7.15 (d, 1H, *J =* 8.2 Hz, Ar); 7.26 (d, 1H, *J =* 1.7 Hz, Ar); 7.31 (dd, 1H, *J =* 1.5, 1.6, 8.4 Hz, Ar); 7.90 (s, 1H, CH=); 7.93 (m, 1H, Ar); 7.96 (m, 1H, Ar); 11.61 (s, 1H, Ar). ^13^C NMR (DMSO-*d_6_*) δ: 55.5 (CH_3_O); 102.3 (OCH_2_O); 109.4 (Ar); 109.8 (Ar); 114.0 (Ar); 116.5 (C=, C-5); 122.8 (C*_ipso_*, Ar); 126.9 (C*_ipso_*, Ar); 127.6 (Ar); 129.9 (Ar); 135.1 (CH=); 148.4 (O-C_Ar_); 150.3 (O-C_Ar_); 162.8 (C*_ipso_*, Ar); 163.5 (C=O); 163.9 (C=O, C-4); 190.5 (C=S, C-2). HRMS, *m*/*z:* 437.0237 found (calculated for C_19_H_14_N_2_O_5_NaS_2_, [M+Na]^+.^ requires 437.0242).

*(5Z)-N-(5-Benzo[1,3]dioxol-5-ylmethylene-4-oxo-2-thioxo-thiazolidin-3-yl)-benzenesulfonamide* (**3t**). According to the standard procedure, **3t** was synthesized at 150 °C with a power of 200 W from *N*-(4-oxo-2-thioxo-thiazolidin-3-yl)-benzenesulfonamide **1e** (100 mg, 0.35 mmol), piperonaldehyde **2a** (52 mg, 0.35 mmol), piperidine (3 μL, 3 mg, 0.034 mmol, 0.1 eq), and glacial acetic acid (2 μL, 2 mg, 0.034 mmol, 0.1 eq) in 64% yield (94 mg) as light-yellow powder; mp = 214–216 °C. ^1^H NMR (DMSO-*d_6_*) δ: 6.16 (s, 2H, OCH_2_O); 7.12 (d, 1H, *J =* 8.1 Hz, Ar); 7.19 (d, 1H, *J =* 1.6 Hz, Ar); 7.24 (dd, 1H, *J =* 1.7, 8.2 Hz, Ar); 7.59 (m, 2H, Ar); 7.69 (m, 1H, Ar); 7.79 (s, 1H, CH=); 7.86 (m, 2H, Ar); 11.57 (br s, 1H, NH). ^13^C NMR (DMSO-*d_6_*) δ: 102.3 (OCH_2_O); 109.4 (Ar); 109.7 (Ar); 115.9 (C=, C-5); 126.8 (C*_ipso_*, Ar); 127.3 (Ar); 127.5 (Ar); 129.1 (Ar); 133.5 (Ar); 134.9 (CH=); 140.7 (C*_ipso_*, Ar); 148.3 (O-C_Ar_); 150.2 (O-C_Ar_); 163.4 (C=O, C-4); 189.6 (C=S, C-2). HRMS, *m*/*z:* 442.9785 found (calculated for C_17_H_12_N_2_O_5_NaS_3_, [M+Na]^+.^ requires 442.9806) and 301.9780 found (calculated for C_11_H_7_N_2_O_3_NaS_2_, [M-SO_2_Ph + Na]^+.^ requires 301.9796).

#### 3.1.4. Standard Procedure for the Preparation of 5-Arylidene-2-thioxo-1,3-thiazolidin-4-one (**3r**) and (**3u**,**v**) in the Monowave^®^ 300 Microwave Reactor

In a 10 mL glass tube, the 3-*N*-amino rhodanine derivative **1** (1 eq), piperonaldehyde **2a** (1 eq), piperidine (0.1 eq) or sodium acetate (0.1 eq), and glacial acetic acid (0.1–6.6 eq) were successively placed. The glass tube was placed in the Monowave^®^ 300 Anton Paar microwave cavity (power = 850 W). The mixture was irradiated at 120 or 150 °C for 20 min under vigorous magnetic stirring. After microwave dielectric heating, the reaction was allowed to cool down to room temperature. To this crude mixture, 2.5 mL of deionized water was added, and the resulting suspension was submitted to ultrasound in a Branson 1510 apparatus at 25 °C for 30 min. Then, the desired insoluble compound **3** was collected by filtration, and 2 mL of absolute ethanol was added to the collected compound **3**. The resulting suspension was stirred vigorously under magnetic stirring for 18 h. The desired compound **3** was finally collected by filtration and was dried under high vacuum (10^−2^ Torr) at 25 °C for 1 h.

*(5Z)-N-(5-Benzo[1,3]dioxol-5-ylmethylene-4-oxo-2-thioxo-thiazolidin-3-yl)-3-phenyl-propionamide* (**3r**). According to the standard procedure, **3r** was synthesized at 120 °C from *N*-(4-oxo-2-thioxo-thiazolidin-3-yl)-3-phenyl-propionamide **1i** (50 mg, 0.18 mmol), piperonaldehyde **2a** (27 mg, 0.18 mmol), sodium acetate (15 mg, 0.18 mmol, 0.1 eq), and glacial acetic acid (68 μL, 396 mg, 6.6 mmol, 6.6 eq) in 68% yield (50 mg) as light-yellow powder; mp = 226–228 °C. ^1^H NMR (DMSO-*d_6_*) δ: 2.65 (m, 2H, CH_2_CO); 2.91 (m, 2H, CH_2_Ph); 6.16 (s, 2H, OCH_2_O); 7.12–7.32 (m, 8H, Ar); 7.85 (s, 1H, CH=); 11.23 (br s, 1H, NH). ^13^C NMR (DMSO-*d_6_*) δ: 30.4 (CH_2_Ar); 34.5 (CH_2_CO); 102.3 (OCH_2_O); 109.4 (Ar); 109.8 (Ar); 116.7 (C=, C-5); 126.1 (Ar); 126.9 (C*_ipso_*, Ar); 127.4 (Ar); 128.3–128.4 (Ar); 134.8 (CH=); 140.5 (C*_ipso_*, Ar); 148.4 (O-C_Ar_); 150.2 (O-C_Ar_); 163.3 (C=O); 170.0 (C=O, C-4); 190.2 (C=S, C-2). HRMS, *m*/*z:* 435.0447 found (calculated for C_20_H_16_N_2_O_4_NaS_2_, [M+Na]^+.^ requires 435.0449).

*(5Z)-(5-Benzo[1,3]dioxol-5-ylmethylene-4-oxo-2-thioxo-thiazolidin-3-yl)-acetic acid* (**3u**). According to the standard procedure, **3u** was synthesized at 120 °C from 2-(4-oxo-2-thioxo-thiazolidin-3-yl)acetic acid **1b** (255 mg, 1.33 mmol), piperonaldehyde **2a** (200 mg, 1.33 mmol), piperidine (13 μL, 11 mg, 0.13 mmol, 0.1 eq), and glacial acetic acid (8 μL, 7.8 mg, 0.13 mmol, 0.1 eq) in 72% yield (308 mg) as light-yellow powder; mp > 250 °C. ^1^H NMR (DMSO-*d_6_*) δ: 4.81 (s, 2H, CH_2_COH); 6.26 (s, 2H, OCH_2_O); 7.23 (d, 1H, *J =* 8.1 Hz, Ar); 7.31–7.35 (m, 2H, Ar); 7.92 (s, 1H, CH=); 12.37 (br s, 1H, OH). ^13^C NMR (DMSO-*d_6_*) δ: 45.5 (CH_2_CO_2_H); 102.0 (OCH_2_O); 109.1 (Ar); 109.4 (Ar); 118.9 (C=, C-5); 126.7 (C*_ipso_*, Ar); 127.0 (Ar); 133.9 (CH=); 148.1 (O-C_Ar_); 149.9 (O-C_Ar_); 166.1 (C=O); 167.1 (C=O, C-4); 192.7 (C=S, C-2). HRMS, *m*/*z:* 322.9922 found (calculated for C_13_H_9_NO_5_S_2_, [M]^+.^ requires 322.9922) and 305.9897 found (calculated for C_13_H_8_NO_4_S_2_, [M − OH]^+^ requires 305.9895).

*(5Z)-3-(5-Benzo[1,3]dioxol-5-ylmethylene-4-oxo-2-thioxo-thiazolidin-3-yl)-propionic acid* (**3v**). According to the standard procedure, **3v** was synthesized at 150°C from 3-(4-oxo-2-thioxo-thiazolidin-3-yl)-propionic acid **1c** (50 mg, 0.24 mmol), piperonaldehyde **2a** (37 mg, 0.24 mmol), piperidine (2 μL, 2 mg, 0.024 mmol, 0.1 eq), and glacial acetic acid (1.4 μL, 1.4 mg, 0.024 mmol, 0.1 eq) in 62% yield (50 mg) as light-yellow powder; mp = 218–220 °C. ^1^H NMR (DMSO-*d_6_*) δ: 2.58 (m, 2H, CH_2_CO_2_H); 4.21 (t, 2H, *J =* 6.9Hz, NCH_2_); 6.15 (s, 2H, OCH_2_O); 7.10–7.24 (m, 3H, Ar); 7.75 (br s, 1H, CH=); 11.95 (br s, 1H, OH). ^13^C NMR (DMSO-*d_6_*) δ: 31.0 (CH_2_CO_2_H); 102.2 (OCH_2_O); 109.3–109.6 (Ar); 119.7 (C*_ipso_*, Ar); 126.8 (Ar); 127.2 (C=, C-5); 133.2 (CH=); 148.3 (O-C_Ar_); 149.9 (O-C_Ar_); 166.7 (C=O); 171.8 (C=O, C-4); 193.0 (C=S, C-2). HRMS, *m*/*z:* 336.0005 found (calculated for C_14_H_10_NO_5_S_2_, [M]^+.^ requires 336.0004).

#### 3.1.5. Standard Procedure for the Preparation of (5*Z*) 2-Amino-5-arylidene-1,3-thiazol-4(5*H*)-4-one (**5a**–**q**) by Sulphur-Nitrogen Displacement in the Synthewave^®^ 402 Microwave Reactor

In a cylindrical quartz reactor (Ø = 1.8 cm), the (5*Z*)-5-arylidene-2-thioxo-1,3-thiazolidin-4-one **3** (0.5 mmol, 1 eq), and the secondary cyclic amine **4** (1.5 mmol, 3 eq) were successively placed. The reactor was then introduced into the Synthewave^®^ 402 Prolabo microwave cavity (power = 300 W). The stirred mixture was irradiated (after a ramp of 3 min from 20 to the appropriate reaction temperature) at 80–120 °C (power: 80–150 W) for 20–120 min. After microwave dielectric heating, the crude reaction mixture was allowed to cooling down at room temperature, and ethanol (20 mL) was added in the cylindrical quartz reactor. The resulting insoluble product **3** was filtered and recrystallized in absolute EtOH and then dried under high vacuum (10^−2^ Torr) for 1 h.

*(5Z)-5-(1,3-Benzodioxol-5-ylmethylene)-2-(morpholin-1-yl)-1,3-thiazol-4(5H)-one* (**5a**). According to the standard procedure, **5a** was synthesized at 80 °C (power: 80 W) after a reaction time of 20 min from (5*Z*)-5-(1,3-benzodioxol-5-ylmethylene)-2-thioxo-1,3-thiazolidin-4-one **3a** (132 mg, 0.5 mmol) and commercial morpholine **4a** (131 μL, 131 mg, 1.5 mmol) in 96% yield (153 mg) as light-yellow powder; mp = 250–252 °C. ^1^H NMR (DMSO-*d_6_*) δ: 3.63 (m, 4H, CH_2_NCH_2_); 3.90 (m, 4H, CH_2_OCH_2_); 6.10 (s, 2H, OCH_2_O); 7.04 (d, 1H, *J =* 8.1 Hz, H-2′); 7.15 (d, 1H, *J =* 8 Hz, H-5′); 7.17 (d, 1H, *J =* 7.9 Hz, H-6′); 7.58 (s, 1H, CH=). ^13^C NMR (DMSO-*d_6_*) δ: 66.1 (CH_2_NCH_2_); 74.4 (CH_2_OCH_2_); 102.2 (OCH_2_O); 109.1 (C-3′); 109.4 (C-5′); 113.9 (CH=); 125.1 (C-6′); 126.9 (C-1′); 130.6 (C=, C-4); 148.6 (C-4′); 149.2 (C-3′); 174.9 (C=O, C-4). HRMS, *m*/*z:* 318.0688 found (calculated for C_15_H_14_N_2_O_4_S, [M]^+.^ requires 318.0674).

*(5Z)-5-(3,4-Dimethoxybenzylidene)-2-(morpholin-1-yl)-1,3-thiazol-4(5H)-one* (**5b**). According to the standard procedure, **5b** was synthesized at 80 °C (power: 80 W) after a reaction time of 25 min from (5*Z*)-5-(3,4-dimethoxybenzylidene)-2-thioxo-1,3-thiazolidin-4-one **3d** (141 mg, 0.5 mmol) and commercial morpholine **4a** (131 μL, 131 mg, 1.5 mmol) in 64% yield (107 mg) as light-yellow powder; mp > 260 °C. ^1^H NMR (DMSO-*d_6_*) δ: 3.73 (m, 4H, CH_2_NCH_2_); 3.74 (s, 3H, OCH_3_); 3.81 (s, 3H, OCH_3_); 3.90 (m, 2H, CH_2_OCH_2_); 7.04 (d, 1H, *J =* 8.1 Hz, H-2′); 7.15 (d, 1H, *J =* 8 Hz, H-5′); 7.17 (d, 1H, *J =* 7.9 Hz, H-6′); 7.58 (s, 1H, CH=). ^13^C NMR (DMSO-*d_6_*) δ: 57.0 (OCH_3_); 57.1 (OCH_3_); 66.3 (CH_2_NCH_2_); 74.40 (CH_2_OCH_2_); 102.2 (OCH_2_O); 109.1 (C-2′); 109.4 (C-5′); 113.9 (CH=); 125.1 (C-6′); 126.9 (C-1′); 130.6 (C=, C-4); 148.6 (C-4′); 149.2 (C-3′); 174.9 (C=O, C-4). HRMS, *m*/*z:* 334.0970 found (calculated for C_16_H_18_N_2_O_4_S, [M]^+.^ requires 334.0987).

*(5Z)-5-(4-Hydroxy-3-methoxybenzylidene)-2-(morpholin-1-yl)-1,3-thiazol-4(5H)-one* (**5c**). According to the standard procedure, **5c** was synthesized at 80 °C (power: 80 W) after a reaction time of 20 min from (5*Z*)-5-(4-hydroxy-3-methoxybenzylidene)-2-thioxo-1,3-thiazolidin-4-one **3b** (134 mg, 0.5 mmol) and commercial morpholine **4a** (131 μL, 131 mg, 1.5 mmol) in 88% yield (141 mg) as light-yellow powder; mp = 251–253 °C. ^1^H NMR (DMSO-*d_6_*) δ: 3.46 (m, 4H, CH_2_NCH_2_); 3.81 (s, 3H, OCH_3_); 3.90 (m, 2H, CH_2_OCH_2_); 7.04 (d, 1H, *J =* 8.1 Hz, H-2′); 7,15 (d, 1H, *J =* 8 Hz, H-5′); 7.17 (d, 1H, *J* = 7.9 Hz, H-6′); 7.58 (s, 1H, CH=); 9.57 (br s, 1H, OH). ^13^C NMR (DMSO-*d_6_*) δ: 58.1 (OCH_3_); 66.1 (CH_2_NCH_2_); 74.5 (CH_2_OCH_2_); 109.1 (C-2′); 109.4 (C-5′); 113.9 (CH=); 125.1 (C-6′); 126.9 (C-1′); 130.6 (C=, C-4); 148.6 (C-4′); 150.4 (C-3′); 174.9 (C=O, C-4). HRMS, *m*/*z:* 320.0836 found (calculated for C_15_H_16_N_2_O_4_S, [M]^+.^ requires 320.0831).

*(5Z)-5-(3-Hydroxy-4-methoxybenzylidene)-2-(morpholin-1-yl)-1,3-thiazol-4(5H)-one* (**5d**). According to the standard procedure, **5d** was synthesized at 80 °C (power: 80 W) after a reaction time of 20 min from (5*Z*)-5-(3-hydroxy-4-methoxybenzylidene)-2-thioxo-1,3-thiazolidin-4-one **3c** (134 mg, 0.5 mmol) and commercial morpholine **4a** (131 μL, 131 mg, 1.5 mmol) in 86% yield (138 mg) as light-yellow powder; mp = 256–258 °C. ^1^H NMR (DMSO-*d_6_*) δ: 3.45 (m, 4H, CH_2_NCH_2_); 3.91 (s, 3H, OCH_3_); 4.01 (m, 2H, CH_2_OCH_2_); 7.04 (d, 1H, *J =* 8.1 Hz, H-2′); 7.15 (d, 1H, *J =* 8 Hz, H-5′); 7.17 (d, 1H, *J =* 7.9 Hz, H-6′); 7.58 (s, 1H, CH=); 9.54 (br s, 1H, OH). ^13^C NMR (DMSO-*d_6_*) δ: 58.1 (OCH_3_); 66.1 (CH_2_NCH_2_); 74.4 (CH_2_OCH_2_); 109.1 (C-2′); 109.4 (C-5′); 113.5 (CH=); 125.3 (C-6′); 126.9 (C-1′); 130.3 (C=, C-4); 148.8 (C-4′); 149.2 (C-3′); 175.1 (C=O, C-4). HRMS, *m*/*z:* 320.0834 found (calculated for C_15_H_16_N_2_O_4_S, [M]^+.^ requires 320.0831).

*(5Z)-5-(1,3-Benzodioxol-5-ylmethylene)-2-(piperazin-1-yl)-1,3-thiazol-4(5H)-one* (**5e**). According to the standard procedure, **5e** was synthesized at 80 °C (power: 80 W) after a reaction time of 60 min from (5*Z*)-5-(1,3-benzodioxol-5-ylmethylene)-2-thioxo-1,3-thiazolidin-4-one **3a** (132 mg, 0.5 mmol) and commercial piperazine **4d** (129 mg, 1.5 mmol) in 78% yield (124 mg) as light-yellow powder; mp > 260 °C. ^1^H NMR (DMSO-*d_6_*) δ: 3.75 (m, 4H, CH_2_NCH_2_); 3.90 (m, 4H, CH_2_NHCH_2_); 6.10 (s, 2H, OCH_2_O); 7.06 (d, 1H, *J =* 8.1 Hz, H-2′); 7.17 (d, 1H, *J =* 8 Hz, H-5′); 7.15 (d, 1H, *J =* 7.9 Hz, H-6′); 7.56 (s, 1H, CH=); 12.58 (br s, 1H, NH). ^13^C NMR (DMSO-*d_6_*) δ: 66.2 (CH_2_NCH_2_); 74.4 (CH_2_NHCH_2_); 102.2 (OCH_2_O); 109.3 (C-2′); 109.1 (C-5′); 113.9 (CH=); 125.1 (C-6′); 126.9 (C-1′); 130.6 (C=, C-4); 148.6 (C-4′); 149.2 (C-3′); 174.9 (C=O, C-4). HRMS, *m*/*z:* 317.0831 found (calculated for C_15_H_15_N_3_O_3_S, [M]^+.^ requires 317.0834).

*(5Z)-5-(1,3-Benzodioxol-5-ylmethylene)-2-(4-N-methylpiperazin-1-yl)-1,3-thiazol-4(5H)-one* (**5f**). According to the standard procedure, **5f** was synthesized at 80 °C (power: 80 W) after a reaction time of 60 min from (5*Z*)-5-(1,3-benzodioxol-5-ylmethylene)-2-thioxo-1,3-thiazolidin-4-one **3a** (132 mg, 0.5 mmol) and commercial *N*-methylpiperazine **4d** (166 μL, 150 mg, 1.5 mmol) in 84% yield (139 mg) as light-yellow powder; mp = 202–204 °C. ^1^H NMR (DMSO-*d_6_*) δ: 3.03 (S, 3H, NCH_3_); 3.73 (m, 4H, CH_2_NCH_2_); 3.90 (m, 4H, CH_2_NMeCH_2_); 6.10 (s, 2H, OCH_2_O); 7.04 (d, 1H, *J =* 8.1 Hz, H-2′); 7.15 (d, 1H, *J =* 8 Hz, H-6′); 7.17 (d, 1H, *J =* 7.9 Hz, H-6′); 7.57 (s, 1H, CH=). ^13^C NMR (DMSO-*d_6_*) δ: 36.1 (NCH_3_); 65.1 (CH_2_NCH_2_); 74.2 (CH_2_NMeCH_2_); 102.2 (OCH_2_O); 109.4 (C-5′); 113.9 (CH=); 125.4 (C-6′); 126.2 (C-1′); 130.6 (C=, C-4); 148.1 (C-4′); 149.6 (C-3′); 173.6 (C=O, C-4). HRMS, *m*/*z:* 331.0962 found (calculated for C_16_H_17_N_3_O_3_S, [M]^+.^ requires 331.0991).

*(5Z)-5-(1,3-Benzodioxol-5-ylmethylene)-2-(thiomorpholin-1-yl)-1,3-thiazol-4(5H)-one* (**5g**). According to the standard procedure, **5g** was synthesized at 80 °C (power: 80 W) after a reaction time of 30 min from (5*Z*)-5-(1,3-benzodioxol-5-ylmethylene)-2-thioxo-1,3-thiazolidin-4-one **3a** (132 mg, 0.5 mmol) and commercial thiomorpholine **4b** (150 μL, 154 mg, 1.5 mmol) in 80% yield (134 mg) as light-yellow powder; mp = 204–208 °C. ^1^H NMR (DMSO-*d_6_*) δ: 3.79 (m, 4H, CH_2_NCH_2_); 3.90 (m, 4H, CH_2_SCH_2_); 6.10 (s, 2H, OCH_2_O); 7.01 (d, 1H, *J =* 8.1 Hz, H-2′); 7.16 (d, 1H, *J =* 8 Hz, H-6′); 7.17 (d, 1H, *J =* 7.8 Hz, H-6′); 7.57 (s, 1H, CH=). ^13^C NMR (DMSO-*d_6_*) δ: 67.0 (CH_2_NCH_2_); 74.4 (CH_2_SCH_2_); 102.7 (OCH_2_O); 109.5 (C-5′); 114.1 (CH=); 125.1 (C-6′); 126.9 (C-1′); 130.8 (C=, C-4); 148.6 (C-4′); 149.2 (C-3′); 174.9 (C=O, C-4). HRMS, *m*/*z:* 334.0441 found (calculated for C_15_H_14_N_2_O_3_S_2_, [M]^+.^ requires 334.0449).

*(5Z)-5-(3,4-Dimethoxybenzylidene)-2-(thiomorpholin-1-yl)-1,3-thiazol-4(5H)-one* (**5h**). According to the standard procedure, **5h** was synthesized at 80 °C (power: 80 W) after a reaction time of 30 min from (5*Z*)-5-(3,4-dimethoxybenzylidene)-2-thioxo-1,3-thiazolidin-4-one **3d** (141 mg, 0.5 mmol) and commercial thiomorpholine **4b** (150 μL, 154 mg, 1.5 mmol) in 78% yield (137 mg) as light-yellow powder; mp = 240–242 °C. ^1^H NMR (DMSO-*d_6_*) δ: 3.73 (m, 4H, CH_2_NCH_2_); 3.74 (s, 3H, OCH_3_); 3.81 (s, 3H, OCH_3_); 3.90 (m, 2H, CH_2_SCH_2_); 7.04 (d, 1H, *J =* 8.1 Hz, H-2′); 7.15 (d, 1H, *J =* 8 Hz, H-5′); 7.17 (d, 1H, *J =* 7.9 Hz, H-6′); 7.60 (s, 1H, CH=). ^13^C NMR (DMSO-*d_6_*) δ: 57.0 (OCH_3_); 57.1 (OCH_3_); 66.0 (CH_2_NCH_2_); 74.4 (CH_2_SCH_2_); 109.1 (C-5′); 113.9 (CH=); 125.1 (C-6′); 126.9 (C-1′); 130.6 (C=, C-4); 148.6 (C-4′); 149.2 (C-3′); 174.9 (C=O, C-4). HRMS, *m*/*z:* 350.0749 found (calculated for C_16_H_18_N_2_O_3_S_2_, [M]^+.^ requires 350.0759).

*(5Z)-5-(1,3-Benzodioxol-5-ylmethylene)-2-(piperidin-1-yl)-1,3-thiazol-4(5H)-one* (**5i**). According to the standard procedure, **5i** was synthesized at 80 °C (power: 80 W) after a reaction time of 20 min from (5*Z*)-5-(1,3-benzodioxol-5-ylmethylene)-2-thioxo-1,3-thiazolidin-4-one **3a** (132 mg, 0.5 mmol) and commercial piperidine **4f** (149 μL, 128 mg, 1.5 mmol) in 80% yield (127 mg) as light-yellow powder; mp = 201–203 °C. ^1^H NMR (DMSO-*d_6_*) δ: 3.79 (m, 4H, CH_2_NCH_2_); 3.90 (m, 4H, CH_2_SCH_2_); 6.10 (s, 2H, OCH_2_O); 7.04 (d, 1H, *J =* 8.1 Hz, H-2′); 7.15 (d, 1H, *J =* 8 Hz, H-5′; 7.17 (d, 1H, *J =* 7.8 Hz, H-6′); 7.58 (s, 1H, CH=). ^13^C NMR (DMSO-*d_6_*) δ: 60.0 (CH_2_CH_2_CH_2_); 65.9 (CH_2_CH_2_CH_2_); 74.4 (CH_2_NCH_2_); 102.3 (OCH_2_O); 109.1 (C-5′); 109.4 (C-5′); 113.7 (CH=); 125.1 (C-6′); 126.6 (C-1′); 130.5 (C=, C-4); 148.8 (C-4′); 149.4 (C-3′); 174.7 (C=O, C-4). HRMS, *m*/*z:* 316.0860 found (calculated for C_16_H_16_N_2_O_3_S, [M]^+.^ requires 316.0882).

*(5Z)-5-(1,3-Benzodioxol-5-ylmethylene)-2-[1-(1,3-benzodioxol-5-ylmethyl)piperazin-1-yl]-1,3-thiazol-4(5H)-one* (**5j**). According to the standard procedure, **5j** was synthesized at 100 °C (power: 80 W) after a reaction time of 30 min from (5*Z*)-5-(1,3-benzodioxol-5-ylmethylene)-2-thioxo-1,3-thiazolidin-4-one **3a** (132 mg, 0.5 mmol) and commercial 1-(1,3-benzodioxol-5-ylmethyl)piperazine **4e** (330 mg, 1.5 mmol) in 75% yield (169 mg) as light-yellow powder; mp > 260 °C. ^1^H NMR (DMSO-*d_6_*) δ: 3.24 (s, 2H, CH_2_N); 3.73 (m, 4H, CH_2_NCH_2_); 3.92 (m, 4H, CH_2_NCH_2_); 6.12 (s, 2H, OCH_2_O); 6.15 (s, 2H, OCH_2_O); 7.06 (d, 1H, *J =* 8.1 Hz, H-2′); 7.09 (d, 1H, *J =* 8.1 Hz, H-2″); 7.15 (d, 1H, *J =* 8 Hz, H-5′); 7.16 (d, 1H, *J =* 8 Hz, H-3″); 7.18 (d, 1H, *J =* 7.9 Hz, H-6′); 7.19 (d, 1H, *J =* 7.9 Hz, H-2″); 7.59 (s, 1H, CH=). ^13^C NMR (DMSO-*d_6_*) δ: 66.1 (CH_2_NCH_2_); 74.6 (CH_2_NCH_2_); 102.4 (OCH_2_O); 103.2 (OCH_2_O); 109.1 (C-2′); 109.2 (Ar); 109.4 (C-5′); 110.1 (Ar); 113.9 (CH=); 125.3 (C-6′); 125.6 (Ar); 126.9 (C-1′); 127.3 (C*_ipso_* Ar); 130.6 (C=, C-4); 148.6 (C-4′); 148.8 (C*_ipso_* Ar); 149.2 (C-3′); 149.8 (Ar); 174.9 (C=O, C-4). HRMS, *m*/*z:* 451.1191 found (calculated for C_21_H_21_N_3_O_5_S, [M]^+.^ requires 451.1202).

*(5Z)-5-(1,3-Benzodioxol-5-ylmethylene)-2-[1-(4-fluorophenyl)piperazin-1-yl]-1,3-thiazol-4(5H)-one (**5k**).* According to the standard procedure, **5k** was synthesized at 120 °C (power: 150 W) after a reaction time of 30 min from (5*Z*)-5-(1,3-benzodioxol-5-ylmethylene)-2-thioxo-1,3-thiazolidin-4-one **3a** (132 mg, 0.5 mmol) and commercial 1-(4-fluorophenyl)piperazine **4g** (270 mg, 1.5 mmol) in 72% yield (148 mg) as light-yellow powder; mp > 260 °C. ^1^H NMR (DMSO-*d_6_*) δ: 3.71 (m, 4H, CH_2_NCH_2_); 3.90 (m, 4H, CH_2_NCH_2_); 6.10 (s, 2H, OCH_2_O); 7.04 (d, 1H, *J =* 8 Hz, H-2′); 7.12 (d, 2H, *J =* 8.1 Hz, H-2″); 7.15 (d, 1H, *J =* 8 Hz, H-5′); 7.16 (d, 1H, *J =* 7.9 Hz, H-6); 7.18 (d, 1H, *J* = 7.9 Hz, H-3″); 7.56 (s, 1H, CH=). ^13^C NMR (DMSO-*d_6_*) δ: 65.9 (CH_2_NCH_2_); 74.4 (CH_2_NCH_2_); 102.7 (OCH_2_O); 109.4 (C-2′); 109.2 (C*_ortho_* Ar); 109.4 (C-5′); 110.1 (C*_meta_* Ar); 113.7 (CH=); 125.1 (C-6′); 125.6 (C-4″); 126.9 (C-1′); 127.3 (Ar); 130.6 (C=, C-4); 148.4 (C-4′); 148.7 (C*_ipso_* Ar); 149.5 (C-3′); 174.5 (C=O, C-4). HRMS, *m*/*z:* 411.1021 found (calculated for C_21_H_18_N_3_O_3_S^19^F, [M]^+.^ requires 411.1053).

*(5Z)-5-(1,3-Benzodioxol-5-ylmethylene)-2-[4-(pyrimidin-2-yl)piperazin-1-yl]-1,3-thiazol-4(5H)-one (**5l**).* According to the standard procedure, **5l** was synthesized at 100 °C (power: 150 W) after a reaction time of 25 min from (5*Z*)-5-(1,3-benzodioxol-5-ylmethylene)-2-thioxo-1,3-thiazolidin-4-one **3a** (132 mg, 0.5 mmol) and commercial 2-(1-piperazin-1-yl)pyrimidine **4h** (212 μL, 246 mg, 1.5 mmol) in 80% yield (158 mg) as light-yellow powder; mp > 260 °C. ^1^H NMR (DMSO-*d_6_*) δ: 3.75 (m, 4H, CH_2_NCH_2_); 3.87 (m, 4H, CH_2_NCH_2_); 6.14 (s, 2H, OCH_2_O); 7.06 (d, 1H, *J =* 7.9 Hz, H-2′); 7.13 (d, 1H, *J =* 8 Hz, H-5′); 7.16 (d, 1H, *J =* 7.9 Hz, H-6′); 7.18 (m, 3H, Ar); 7.61 (s, 1H, CH=). ^13^C NMR (DMSO-*d_6_*) δ: 66.0 (CH_2_NCH_2_); 74.2 (CH_2_NCH_2_); 102.5 (OCH_2_O); 109.2 (C-2′); 109.4 (C-5′); 110.1 (Ar); 113.9 (CH=); 125.2 (C-6′); 125.6 (C*_ipso_* Ar); 126.9 (C-1′); 130.5 (C=, C-4); 148.6 (C-4′); 148.8 (C*_ipso_* Ar); 149.2 (C-3′); 174.7 (C=O, C-4). HRMS, *m*/*z:* 395.1006 found (calculated for C_19_H_17_N_5_O_3_S, [M]^+.^ requires 395.1052).

*(5Z)-5-(2,3-Dihydro-benzofuran-5-ylmethylene)-2-(morpholin-4-yl)-1,3-thiazol-4(5H)-one(**5m**).* According to the standard procedure, **5m** was synthesized at 80 °C (power: 90 W) after a reaction time of 20 min from (5*Z*)-5-(2,3-dihydro-benzofuran-5-ylmethylene)-2-thioxo-1,3-thiazolidin-4-one **3g** (131 mg, 0.5 mmol) and commercial morpholine **4a** (131 μL, 131 mg, 1.5 mmol) in 80% yield (127 mg) as light-yellow powder; mp = 222–224 °C. ^1^H NMR (DMSO-*d_6_*) δ: 3.70 (t, 2H, *J* = 6.8 Hz; CH**_2_**CH_2_O); 3.73 (m, 4H, CH_2_NCH_2_); 3.90 (t, 2H, *J* = 6.9 Hz; CH_2_CH_2_O); 5.20 (m, 2H, CH_2_OCH_2_); 7.04 (d, 1H, *J =* 8.1 Hz, H-2′); 7.15 (d, 1H, *J =* 8 Hz, H-5′); 7.17 (d, 1H, *J =* 7.9 Hz, H-6′); 7.58 (s, 1H, CH=). ^13^C NMR (DMSO-*d_6_*) δ: 29.2 (CH_2_CH_2_O); 65.9 (CH_2_NCH_2_); 71.7 (CH_2_CH_2_O); 74.4 (CH_2_OCH_2_); 109.1 (C-2′); 109.5 (C-5′); 113.9 (CH=); 125.1 (C-6′); 126.9 (C-1′); 130.6 (C=, C-4); 148.6 (C-4′); 149.4 (C-3′); 174.9 (C=O, C-4). HRMS, *m*/*z:* 316.0864 found (calculated for C_16_H_16_N_2_O_3_S, [M]^+.^ requires 316.0882).

*(5Z)-5-(2,3-Dihydro-benzofuran-5-ylmethylene)-2-(thiomorpholin-4-yl)-1,3-thiazol-4(5H)-one(**5n**).* According to the standard procedure, **5n** was synthesized at 80 °C (power: 90 W) after a reaction time of 20 min from (5*Z*)-5-(2,3-dihydro-benzofuran-5-ylmethylene)-2-thioxo-1,3-thiazolidin-4-one **3g** (131 mg, 0.5 mmol) and commercial thiomorpholine **4b** (150 μL, 154 mg, 1.5 mmol) in 84% yield (140 mg) as light-yellow powder; mp = 230–232 °C. ^1^H NMR (DMSO-*d_6_*) δ: 3.73 (t, 2H, *J* = 6.8 Hz; CH_2_CH_2_O); 4.02 (m, 4H, CH_2_NCH_2_); 4.09 (m, 4H, CH_2_SCH_2_); 5.22 (t, 2H, *J* = 6.9 Hz; CH_2_CH_2_O); 7.02 (d, 1H, *J =* 8.1 Hz, H-2′); 7.13 (d, 1H, *J =* 8 Hz, H-5′); 7.19 (d, 1H, *J =* 7.9 Hz, H-6′); 7.60 (s, 1H, CH=). ^13^C NMR (DMSO-*d_6_*) δ: 29.3 (CH_2_CH_2_O); 66.1 (CH_2_NCH_2_); 71.7 (CH_2_SCH_2_); 74.4 (CH_2_CH_2_O); 108.9 (C-2′); 109.4 (C-5′); 113.7 (CH=); 125.2 (C-6′); 126.9 (C-1′); 130.7 (C=, C-4); 148.4 (C-4′); 149.2 (C-3′); 174.6 (C=O, C-4). HRMS, *m*/*z:* 332.0643 found (calculated for C_16_H_16_N_2_O_2_S_2_, [M]^+.^ requires 332.0653).

*(5Z)-5-(2,3-Dihydro-benzofuran-5-ylmethylene)-2-(piperidin-1-yl)-1,3-thiazol-4(5H)-one(**5o**).* According to the standard procedure, **5o** was synthesized at 80 °C (power: 90 W) after a reaction time of 20 min from (5*Z*)-5-(2,3-dihydro-benzofuran-5-ylmethylene)-2-thioxo-1,3-thiazolidin-4-one **3g** (131 mg, 0.5 mmol) and commercial piperidine **4f** (149 μL, 128 mg, 1.5 mmol) in 86% yield (135 mg) as light-yellow powder; mp = 252–254 °C. ^1^H NMR (DMSO-*d_6_*) δ: 3.71 (m, 6H, CH_2_); 3.73 (t, 2H, *J* = 6.8 Hz; CH_2_CH_2_O); 4.02 (m, 4H, CH_2_NCH_2_); 5.22 (t, 2H, *J* = 6.9 Hz; CH_2_CH_2_O); 7.06 (d, 1H, *J =* 8 Hz, H-2′); 7.15 (d, 1H, *J =* 8.2 Hz, H-5′); 7.17 (d, 1H, *J =* 7.9 Hz, H-6′); 7.56 (s, 1H, CH=). ^13^C NMR (DMSO-*d_6_*) δ: 24.1 (CH_2_CH_2_CH_2_); 29.1 (CH_2_CH_2_CH_2_); 31.2 (CH_2_CH_2_O); 66.2 (CH_2_NCH_2_); 74.3 (CH_2_CH_2_O); 109.1 (C-2′); 109.3 (C-5′); 113.9 (CH=); 125.1 (C-6′); 126.8 (C-1′); 130.6 (C=, C-4); 148.6 (C-4′); 149.3 (C-3′); 174.9 (C=O, C-4). HRMS, *m*/*z:* 314.1075 found (calculated for C_17_H_18_N_2_O_2_S, [M]^+.^ requires 314.1089).

*(5Z)-5-(2,3-Dihydro-1,4-benzodioxin-6-ylmethylene)-2-(piperidin-1-yl)-1,3-thiazol-4(5H)-one (**5p**).* According to the standard procedure, **5p** was synthesized at 80 °C (power: 90 W) after a reaction time of 20 min from (5*Z*)-5-(2,3-dihydro-1,4-benzodioxin-6-ylmethylene)-2-thioxo-1,3-thiazolidin-4-one **3f** (140 mg, 0.5 mmol) and commercial piperidine **4f** (149 μL, 128 mg, 1.5 mmol) in 88% yield (145 mg) as light-yellow powder; mp = 257–259 °C. ^1^H NMR (DMSO-*d_6_*) δ: 2.93 (m, 6H, CH_2_); 3.72 (m, 4H, CH_2_NCH_2_); 4.28 (t, 2H, OCH_2_CH_2_O); 4.30 (t, 2H, OCH_2_CH_2_O); 7.04 (d, 1H, *J =* 8.1 Hz, H-2′); 7,14 (d, 1H, *J =* 8 Hz, H-5′); 7.18 (d, 1H, *J =* 7.9 Hz, H-6′); 7.60 (s, 1H, CH=). ^13^C NMR (DMSO-*d_6_*) δ: 24.0 (CH_2_CH_2_CH_2_); 29.0 (CH_2_CH_2_CH_2_); 63.1 (CH_2_NCH_2_); 64.8 (OCH_2_CH_2_O); 66.4 (OCH_2_CH_2_O); 109.2 (C-2′); 109.4 (C-5′); 113.7 (CH=); 125.2 (C-6′); 126.9 (C-1′); 130.7 (C=, C-4); 148.7 (C-4′); 149.2 (C-3′); 175.1 (C=O, C-4). HRMS, *m*/*z:* 330.1081 found (calculated for C_17_H_18_N_2_O_3_S, [M]^+.^ requires 330.1038).

*(5Z)-5-(2,3-Dihydro-1,4-benzodioxin-6-ylmethylene)-2-(thiomorpholin-4-yl)-1,3-thiazol-4(5H)-one (**5q**).* According to the standard procedure, **5q** was synthesized at 80 °C (power: 90 W) after a reaction time of 20 min from (5*Z*)-5-(2,3-dihydro-1,4-benzodioxin-6-ylmethylene)-2-thioxo-1,3-thiazolidin-4-one **3f** (140 mg, 0.5 mmol) and commercial thiomorpholine **4b** (150 μL, 154 mg, 1.5 mmol) in 80% yield (139 mg) as light-yellow powder; mp > 260 °C. ^1^H NMR (DMSO-*d_6_*) δ: 3.71 (m, 4H, CH_2_NCH_2_); 3.90 (m, 2H, CH_2_SCH_2_); 4.27 (t, 2H, OCH_2_CH_2_O); 4.30 (t, 2H, OCH_2_CH_2_O); 5.10 (m, 2H, CH_2_OCH_2_); 7.01 (d, 1H, *J =* 8.2 Hz, H-2′); 7.15 (d, 1H, *J =* 8 Hz, H-5′); 7.19 (d, 1H, *J =* 7.9 Hz, H-6′); 7.58 (s, 1H, CH=). ^13^C NMR (DMSO-*d_6_*) δ: 64.8 (OCH_2_CH_2_O); 66.2 (CH_2_NCH_2_O); 66.6 (OCH_2_CH_2_O); 74.40 (CH_2_SCH_2_); 102.2 (OCH_2_O); 109.0 (C-2′); 109.4 (C-5′); 113.9 (CH=); 125.1 (C-6′); 126.9 (C-1′); 130.8 (C=, C-4); 148.6 (C-4′); 149.4 (C-3′); 174.9 (C=O, C-4). HRMS, *m*/*z:* 348.0603 found (calculated for C_16_H_16_N_2_O_3_S_2_, [M]^+.^ requires 348.0602).

#### 3.1.6. Standard Procedure for the Preparation of (5*Z*) 2-*N*-Heteroarylamino-5-arylidene-1,3-thiazol-4(5*H*)-one Derivatives (**5r**–**x**) by Solution Phase Condensation under Microwave in the Explorer^®^ 24 Reactor

In a 10 mL glass tube, the 2-*N*-heteroarylamino-thiazolidin-4-one **8** (1 eq), aromatic aldehyde **2** (1 eq), piperidine (0.1 eq) or sodium acetate (0.1 eq), and glacial acetic acid (0.1–6.6 eq) were successively placed. The glass tube was placed in the Explorer^®^ 24 CEM microwave cavity (power = 300 W). The mixture was irradiated at 120 or 150 °C (with a power of 100 or 200 W) for 20 or 40 min under vigorous magnetic stirring. After microwave dielectric heating, the reaction was allowed to cool down to room temperature. To this crude mixture, 1–2 mL of deionized water was added, and the resulting suspension was submitted to ultrasound in a Branson 1510 apparatus at 25 °C for 30 min. Then, the desired insoluble compound **3** was collected by filtration, and 2 mL of absolute ethanol was added to this collected compound **5**. The resulting suspension was stirred vigorously under magnetic stirring for 18 h. The desired compound **3** was finally collected by filtration and was dried under high vacuum (10^−2^ Torr) at 25 °C for 1 h.

*(5Z)-5-Benzo[1,3]dioxol-5-ylmethylene-2-phenylamino-1,3-thiazol-4(5H)-one (**5r**).* According to the standard procedure, **5r** was synthesized at 150 °C with a power of 200 W for 20 min from 2-phenylamino-1,3-thiazolidin-4-one **8a** (192 mg, 1 mmol), piperonaldehyde **2a** (150 mg, 1 mmol), piperidine (10 μL, 9 mg, 0.1 mmol, 0.1 eq), and glacial acetic acid (6 μL, 6 mg, 0.1 mmol, 0.1 eq) in 34% yield (100 mg) as reddish powder; mp > 250 °C. ^1^H NMR (DMSO-*d_6_*) δ: 6.08 (s, 2H, OCH_2_O); 7.03–7.22 (m, 5H, Ar + CH=); 7.42 (q, *J =* 7.4Hz, 2H, Ar); 7.56–7.78 (m, 2H, Ar); 12.21 (br s, 1H, NH). ^13^C NMR (DMSO-*d_6_*) δ: 101.8 (OCH_2_O); 108.7 (C-4′); 109.0 (C-7′); 120.4 (C-6′); 121.0 (C*_ipso_*, C-5′); 121.4 (C-2″); 124.8 (C-2″); 125.2 (C-4″); 127.5 (C=, C-5); 129.1 (C-3″); 129.4 (C-3″); 130.6 (CH=); 147.9 (C*_ipso_*, C-1″); 148.1 (C*_ipso_*, C-3a′); 148.7 (C*_ipso_*, C-7a′); 170.4 (C=N, C-2); 180.5 (C=O, C-4). HRMS, *m*/*z:* 325.0646 found (calculated for C_17_H_13_N_2_O_3_S, [M+H]^+.^ requires 325.0647).

*(5Z)-5-Benzo[1,3]dioxol-5-ylmethylene-2-(pyridin-2-yl)amino-1,3-thiazol-4(5H)-one* (**5s**). According to the standard procedure, **5s** was synthesized at 120 °C with a power of 100 W for 20 min from 2*-(*pyridin-2-yl)amino-1,3-thiazolidin-4-one **8b** (50 mg, 0.26 mmol), piperonaldehyde **2a** (39 mg, 0.26 mmol), sodium acetate (21 mg, 0.26 mmol, 1 eq), and glacial acetic acid (0.1 mL, 102 mg, 1.7 mmol, 6.6 eq) in 90% yield (76 mg) as green-yellowish powder; mp > 250 °C. ^1^H NMR (DMSO-*d_6_*) δ: 6.13 (s, 2H, OCH_2_O); 7.12 (d, *J =* 7.9 Hz, 1H, CH=); 7.17–7.23 (m, 4H, H-7′, H-4′, H-3″, H-5″); 7.58 (m, 1H, H-6′); 7.85 (t, *J =* 7.6 Hz, 1H, H-4″); 8.52 (d, *J =* 4.1 Hz, 1H, H-6″); 12.39 (br s, 1H, NH). ^13^C NMR (DMSO-*d_6_*) δ: 101.9 (OCH_2_O); 109.0 (C-4′); 120.1 (Ar); 127.8 (C=, C-5); 130.2 (Ar); 131.1 (CH=); 133.1 (Ar); 138.9 (Ar); 145.6 (C*_ipso_*, C-2″); 148.0 (C*_ipso_*, C-3a′); 148.8 (C*_ipso_*, C-7a′); 163.6 (C=N, C-2); 187.4 (C=O, C-4). HRMS, *m*/*z:* 348.0418 found (calculated for C_16_H_11_N_3_O_3_SNa, [M+Na]^+.^ requires 348.0419).

*(5Z)-5-Benzo[1,3]dioxol-5-ylmethylene-2-(pyridin-4-yl)amino-1,3-thiazol-4(5H)-one* (**5t**). According to the standard procedure, **5t** was synthesized at 120 °C with a power of 100 W for 40 min from 2-(pyridin-4-yl)amino-1,3-thiazolidin-4-one **8c** (150 mg, 0.78 mmol), piperonaldehyde **2a** (117 mg, 0.78 mmol), sodium acetate (64 mg, 0.78 mmol, 1 eq), and glacial acetic acid (295 μL, 307 mg, 5.11 mmol, 6.6 eq) in 43% yield (109 mg) as orange-yellowish powder; mp > 250 °C. ^1^H NMR (DMSO-*d_6_*) δ: 6.11 (s, 2H, OCH_2_O); 7.11 (m, 2H, H-3″); 7.13 (s, 1H, H-7′); 7.72–7.67 (m, 3H, CH=, H-6′, H-4′); 8.72 (m, 2H, H-2″). ^13^C NMR (DMSO-*d_6_*) δ: 102.4 (OCH_2_O); 109.6–109.6 (C-7′, C-3″); 117.9 (C-4′); 126.1 (C-6′); 127.6 (C-5′); 132.4 (CH=); 146.3–146.3 (C-2″); 149.2–148.1 (C-3a′, C-7a′); 170.4 (C=N, C-2); 180.5 (C=O, C-4). HRMS, *m*/*z:* 370.0243 found (calculated for C_16_H_10_N_3_O_3_Na_2_S, [M-H+2Na]^+.^ requires 370.0238).

*(5Z)-5-Benzo[1,3]dioxol-5-ylmethylene-2-(4-methoxy-phenylamino)-1,3-thiazol-4(5H)-one* (**5u**). According to the standard procedure, **5u** was synthesized at 120 °C with a power of 100 W for 20 min from 2-(4-methoxyphenyl)amino-1,3-thiazolidin-4-one **8d** (50 mg, 0.22 mmol), piperonaldehyde **2a** (34 mg, 0.22 mmol), sodium acetate (18 mg, 0.22 mmol, 1 eq), and glacial acetic acid (80 μL, 87 mg, 1.45 mmol, 6.6 eq) in 90% yield (72 mg) as light-yellow powder; mp > 250 °C. ^1^H NMR (DMSO-*d_6_*) δ: 3.76 (s, 3H, CH_3_O); 6.13 (s, 2H, OCH_2_O); 7.04–7.16 (m, 5H, CH=, Ar); 7.55–7.70 (m, 3H, Ar); 11.56 (br s, 1H, NH). ^13^C NMR (DMSO-*d_6_*) δ: 55.3 (CH_3_O); 101.8 (OCH_2_O); 108.7 (C-5′); 109.0 (C-4′); 114.2 (C-3″); 114.6 (C-3″); 122.0 (C-7′); 123.1 (C=, C-5); 124.8 (C-2″); 125.1 (C-2″); 127.7 (C*_ipso_*, C-1″); 128.2 (C-4″); 129.4 (C-6′); 130.0 (CH=); 147.9 (C-3a′); 148.1 (C-7a′); 148.7 (C=O, C-4); 156.5 (C=N, C-2). HRMS, *m*/*z:* 377.0574 found (calculated for C_18_H_14_N_2_O_4_NaS, [M+Na]^+.^ requires 377.0572).

*(5Z)-5-Benzo[1,3]dioxol-5-ylmethylene-2-(4-chloro-phenylamino)-1,3-thiazol-4(5H)-one* (**5v**). According to the standard procedure, **5v** was synthesized at 120 °C with a power of 100 W for 20 min from 2-(4-chlorophenyl)amino-1,3-thiazolidin-4-one **8e** (50 mg, 0.22 mmol), piperonaldehyde **2a** (34 mg, 0.22 mmol), sodium acetate (18 mg, 0.22 mmol, 1 eq), and glacial acetic acid (80 μL, 87 mg, 1.45 mmol, 6.6 eq) in 79% yield (62 mg) as light-yellow powder; mp > 250 °C. ^1^H NMR (DMSO-*d_6_*) δ: 6.08 (s, 2H, OCH_2_O); 7.04–7.17 (m, 4H, Ar); 7.43–7.51 (m, 2H, Ar); 7.57–7.68 (m, 2H, Ar); 7.81 (d, *J =* 7.2 Hz, 1H, CH=); 11.65 (br s, 1H, NH). ^13^C NMR (DMSO-*d_6_*) δ: 101.8 (OCH_2_O); 108.8 (C-4″); 109.1 (C-5′); 109.3 (C-4′); 122.0 (C-7′); 123.1 (C-2″); 124.9 (C-2″); 125.3 (C-5′); 127.4 (C=, C-5); 129.0 (C-1″); 129.4 (C-4″); 129.8 (C-6′); 131.0 (CH=); 148.0 (C-3a′); 148.1 (C-7a′); 148.8 (C=O, C-4); 156.7 (C=N, C-2). HRMS, *m*/*z:* 381.0079 found (calculated for C_17_H_11_N_2_O_3_^35^ClSNa, [M+Na]^+.^ requires 381.0077).

*(5Z)-5-(2,3-Dihydro-benzofuran-5-ylmethylene)-2-phenylamino-1,3-thiazol-4(5H)-one* (**5w**). According to the standard procedure, **5w** was synthesized at 150 °C with a power of 200 W for 20 min from 2-phenylamino-1,3-thiazolidin-4-one **8a** (100 mg, 0.22 mmol), 2,3-dihydro-1-benzofuran-5-carbaldehyde **2b** (77 mg, 0.52 mmol), piperidine (5 mL, 4 mg, 0.052 mmol, 0.1 eq), and glacial acetic acid (3 μL, 3 mg, 0.052 mmol, 0.1 eq) in 64% yield (107 mg) as light-yellow powder; mp > 250 °C. ^1^H NMR (DMSO-*d_6_*) δ: 3.21 (m, 2H, CH_2_); 4.54–4.65 (m, 2H, CH_2_O); 6.90 (m, 1H, H-2″); 7.03 (m, 1H, H-4″); 7.19 (t, *J =* 7.4 Hz, 1H, H-7′); 7.27–7.66 (m, 5H, H-4′, H-6′, H-3″); 7.77 (d, *J =* 7.7 Hz, 1H, CH=); 12.25 (br s, 1H, NH). ^13^C NMR (DMSO-*d_6_*) δ: 28.5 (CH_2_); 71.8 (OCH_2_); 108.7 (C-7′); 120.4 (C-2″); 121.4 (C-2″); 124.8 (C-4″); 125.9 (C-3a′); 126.5 (C-6′); 128.9 (C=, C-5); 129.0 (C-1″); 129.2 (C-4′); 129.4 (C-3″); 129.9 (C-3″); 131.0 (CH=); 148.0 (C-3a′); 148.1 (C-7a′); 161.4 (C=O, C-4); 206.7 (C=N, C-2). HRMS, *m*/*z:* 345.0676 found (calculated for C_18_H_14_N_2_O_2_SNa, [M+Na]^+.^ requires 345.0674).

*(5Z)-5-(2,3-Dihydro-benzo[1,4]dioxin-6-ylmethylene)-2-phenylamino-1,3-thiazol-4(5H)-one* (**5x**). According to the standard procedure, **5x** was synthesized at 120 °C with a power of 100 W for 20 min from 2-phenylamino-1,3-thiazolidin-4-one **8a** (100 mg, 0.52 mmol), 2,3-dihydro-1,4-benzodioxine-6-carbaldehyde **2c** (85 mg, 0.52 mmol), sodium acetate (43 mg, 0.52 mmol, 1 eq), and glacial acetic acid (0.2 μL, 205 mg, 3.42 mmol, 6.6 eq) in 98% yield (175 mg) as light-yellow powder; mp > 250 °C. ^1^H NMR (DMSO-*d_6_*) δ: 4.24–4.33 (m, 4H, OCH_2_CH_2_O); 6.93–7.06 (m, 2H, H-2″); 7.12 (m, 1H, H-8′); 7.20 (t, *J =* 7.4 Hz, 1H, H-4″); 7.42 (m, 2H, H-3″); 7.52 (m, 1H, H-5′); 7.62 (m, 1H, H-7′); 7.77 (m, 1H, CH=); 12.26 (br s, 1H, NH). ^13^C NMR (DMSO-*d_6_*) δ: 63.9–64.4 (CH_2_CH_2_O); 117.7 (C-5′); 117.8 (C-8′); 120.5 (C-2″); 121.0 (C-1′); 121.4 (C-2″); 123.3 (C-7′); 124.9 (C-4″); 126.7 (C=, C-5); 127.1 (C-1″); 129.1 (C-3″); 129.4 (C-3″); 130.4 (CH=); 143.5 (C-4a′); 145.1 (C-8a′); 170.5 (C=O, C-4); 117.3 (C=N, C-2). HRMS, *m*/*z:* 361.0623 found (calculated for C_18_H_14_N_2_O_3_SNa, [M+Na]^+.^ requires 361.0623).

### 3.2. Biochemistry Part

#### 3.2.1. Protein Kinase Assay Buffers

*Buffer A*: 10 mM MgCl_2_, 1 mM EGTA, 1 mM DTT, 25 mM Tris-HCl pH 7.5, 50 µg heparin/mL.

*Buffer C:* 60 mM β-glycerophosphate, 15 mM *p*-nitrophenyl-phosphate, 25 mM Mops (pH 7.2), 5 mM EGTA, 15 mM MgCl_2_, 1 mM DTT, 1 mM sodium vanadate, 1 mM phenylphosphate.

#### 3.2.2. Kinase Preparations and Assays

Kinase activities for each enzyme were assayed in buffer A or C, with their corresponding substrates, in the presence of 15 µM ATP in a final volume of 30 µL. After 30-min incubation at 30 °C, the reaction was stopped by harvesting, using a FilterMate harvester (Packard), onto P81 phospho-cellulose papers (GE Healthcare, Chicago, IL, USA) which were washed in 1% phosphoric acid. Scintillation fluid was added and the radioactivity measured in a Packard counter. Blank values were subtracted and activities calculated as pmoles of phosphate incorporated for the 30-min incubation. The activities were expressed in % of the maximal activity, i.e., in the absence of inhibitors. Controls were performed with appropriate dilutions of DMSO.

*CDK5*/*p25* (human, recombinant) was prepared as previously described [57]. Its kinase activity was assayed in buffer C, with 1 mg histone H1/mL, in the presence of 15 μM [γ-^33^P] ATP (3000 Ci/mmol; 10 mCi/mL) in a final volume of 30 μL. After 30-min incubation at 30 °C, 25 μL aliquots of supernatant were spotted onto 2.5 × 3 cm pieces of Whatman P81 phospho-cellulose paper, and, 20 s later, the filters were washed five times (for at least 5 min each time) in a solution of 10 mL phosphoric acid/litter of water. The wet filters were counted in the presence of 1 mL ACS (Amersham, UK) scintillation fluid.

*DYRK1A* (rat, recombinant, expressed in *Escherichia coli* as a GST fusion protein, provided by Dr. W. Becker) was purified by affinity chromatography on glutathione–agarose and assayed as described for CDK5/p25 using myelin basic protein (1 mg/mL) as a substrate.

*Casein kinase 1* (*CK1δ*/*ε*) (porcine brain, native) was assayed with 0.67 µg of CKS peptide (RRKHAAIGpSAYSITA), a CK1 specific substrate obtained from Millegen (Labege, France) [58].

*GSK-3α*/*β* (porcine brain, native) was assayed, as described for CDK5/p25 but in Buffer A and using a GSK-3 specific substrate (GS-1: YRRAAVPPSPSLSRHSSPHQSpEDEEE) (pS stands for phosphorylated serine) [59]. GS-1 was synthesized by Millegen (Labege, France).

#### 3.2.3. Cell Culture and Survival Assays

Skin diploid fibroblastic cells were provided by BIOPREDIC International Company (Rennes, France). Caco2 (Ref ECACC: 86010202), Huh-7D12 (Ref ECACC: 01042712), MDA-MB-231 (Ref ECACC: 92020424), HCT-116 (Ref ECACC: 91091005), PC3 (Ref ECACC: 90112714), and NCI-H727 (Ref ECACC: 94060303) cell lines were obtained from the ECACC collection. Cells were grown according to ECACC recommendations [60]. The toxicity test of the compounds on these cells was as follows: 2 × 10^3^ cells for HCT-116 cells or 4 × 10^3^ for the other cells were seeded in 96-multi-well plates in triplicate and left for 24 h for attachment, spreading and growing. Then, cells were exposed for 48 h to increasing concentrations of the compounds, ranging from 0.1 to 25 mM, in a final volume of 120 μL of culture medium. Cells were fixed in cooled 90% ethanol/5% acetic acid solution, and nuclei were stained with Hoechst 3342 (Sigma) and counted using automated imaging analysis (Cellomics Arrayscan VTI/HCS Reader, Thermo/Scientific, Waltham, MA, USA). The IC_50_ were graphically determined.

## 4. Conclusions

All the data presented in this study demonstrate the interest and the potential of the 1,3-thiazolidin-4-one core in inhibition of the protein kinase DYRK1A. In this preliminary project of pharmacology, we have developed two series of new compounds under microwave irradiation. In the first library, the twenty-two 5-arylidene-2-thioxo-1,3-thiazolidin-4-ones **3a**–**v** were synthesized in yields ranging from 26 to 98% by microwave irradiation assisted Knoevenagel condensation using an “open or closed vessel” approach using organic bases (propylamine, TEA, and AcONa). Next, most of the compounds **3** were transformed into 2-amino-5-arylidene-1,3-thiazol-4(5*H*)-ones **5a**–**q** under microwave irradiation by sulphur/nitrogen displacement with appropriate secondary cyclic amines **4a**–**h**. This second family was completed by the preparation of seven new 2-*N*-heteroarylamino-5-arylidene-1,3-thiazol-4(5*H*)-ones **5r**–**x** from the 2-*N*-heteroarylamino-1,3-thiazolidin-4-one building blocks **8a**–**e** under microwave irradiation (34–98% yield). In the two libraries, all the 46 compounds **3** and **5** have been built with a *Z*-geometry and were evaluated against four protein kinases. Among all these compounds, nine of them turned out to be very interesting because they presented sub-micromolar inhibition activity on DYRK1A (0.090 μM > IC_50_ > 0.028 μM). The most effective compounds in these two libraries are the molecules **3e** and **5s**, as new nano-molar DYRK1A inhibitors (**3e**: IC_50_ 0.028 μM and **5s**: IC_50_ 0.033 μM). The current results are the starting point of a new larger program within our group to investigate the biological properties of these new inhibitors with potential application in Alzheimer’s disease or in cancer.

## Figures and Tables

**Table 1:** Results for the preparation under microwave irradiation of (5*Z*) 5-arylidene-2-thioxo-1,3-thiazolidin-4-one derivatives **3a**–**v** from aromatic aldehydes **2a**–**p** and 2-thioxo-1,3-thiazolidin-4-ones **1a**–**i**.

Compound 3		Reagents	Reactions Conditions Used under Microwave *^a^*				Yield *^b^* of 3 (%) *^d^*
			Starting Aldehyde 2	Reaction Temp. (°C)	Reaction Time (min.)	Power (W)	
**3a**	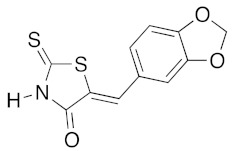	*n*-PrNH_2_ 2 eq	**2a**	80	60	90 *^c^*	79
**3b**	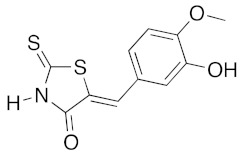	*n*-PrNH_2_ 2 eq	**2d**	80	60	90 *^c^*	81
**3c**	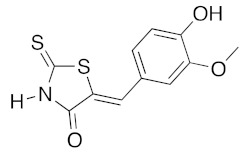	*n*-PrNH_2_ 4 eq	**2e**	80	60	90 *^c^*	80
**3d**	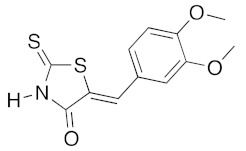	*n*-PrNH_2_ 2 eq	**2h**	80	60	90 *^c^*	83
**3e**	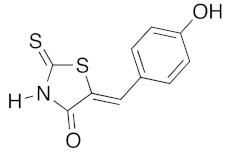	*n*-PrNH_2_ 2 eq	**2i**	80	40	90 *^c^*	79
**3f**	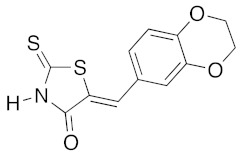	*n*-PrNH_2_ 2 eq	**2c**	80	60	90 *^c^*	89
**3g**	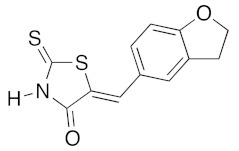	*n*-PrNH_2_ 2 eq	**2b**	80	60	90 c	79
**3h**	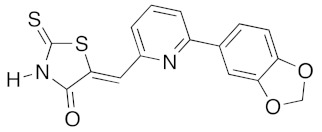	*n*-PrNH_2_ 2 eq	**2k**	80	60	90 *^c^*	68
**3i**	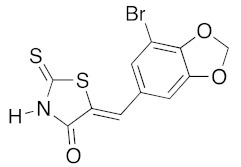	*n*-PrNH_2_ 1 eq	**2l**	80	60	90 *^c^*	70
**3j**	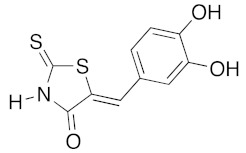	*n*-PrNH_2_ 2 eq	**2f**	80	60	90 *^c^*	79
**3k**	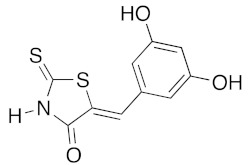	*n*-PrNH_2_ 2 eq	**2n**	80	60	90 *^c^*	79
**3l**	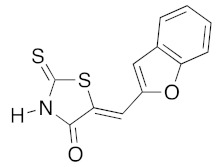	*n*-PrNH_2_ 2 eq	**2m**	80	60	90 *^c^*	62
**3m**	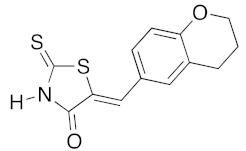	AcONa 1.2 eq AcOH 1.2 eq	**2o**	140	20	- *^e^*	93
**3n**	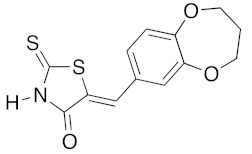	AcONa 1.2 eq AcOH 1.2 eq	**2p**	140	30	- *^e^*	94
**3o**	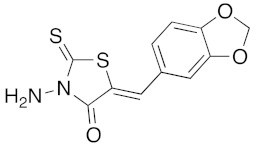	Et_3_N 0.1 eq AcOH 0.1 eq AcOEt	**2a**	85	210	-*^f^*	26
**3p**	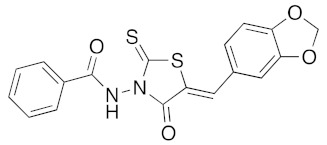	piperidine 0.1 eq AcOH 0.1 eq	**2a**	150	20	200 *^d^*	27
**3q**	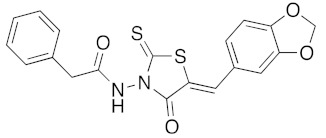	AcONa 1 eq AcOH 6.6 eq	**2a**	120	20	100 *^d^*	98
**3r**	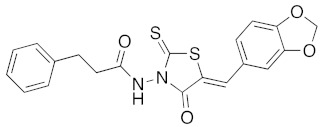	AcONa 1 eq AcOH 6.6 eq	**2a**	120	20	- *^e^*	68
**3s**	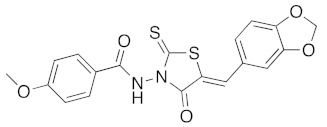	AcONa 1 eq AcOH 6.6 eq	**2a**	120	20	100 *^d^*	83
**3t**	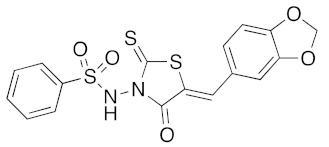	piperidine 0.1 eq AcOH 0.1 eq	**2a**	150	20	200 *^d^*	64
**3u**	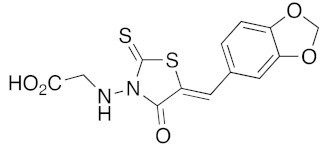	AcONa 0.1 eq AcOH 0.1 eq	**2a**	150	20	- *^e^*	72
**3v**	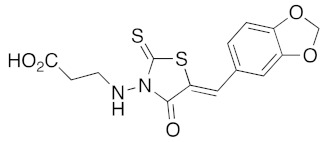	piperidine 0.1 eq AcOH 0.1 eq	**2a**	150	20	- *^e^*	62

*^a^* Microwave irradiation (MWI) of the reaction mixture was realized in an open glass microwave reactor or in tube sealed with a snap cap (closed vessel). *^b^* Isolated yield after work up, crystallization, and washing using appropriate solvent. *^c^* Synthewave^®^ 402 (Merck Eurolab Prolabo France) used as microwave reactor (Power max.: 300 W). Reaction under microwave was realized in an open glass reactor. *^d^* Explorer^®^ 24 (CEM μWave France) used as a microwave reactor (Power max.: 300 W). Reaction under microwave was realized in a tube sealed with a snap cap. *^e^* Monowave^®^ 300 (Anton Paar France) used as a microwave reactor (Power max.: 800 W). Reaction under microwave was realized in a tube sealed with a snap cap. *^f^* Compound **3o** was synthesized in an oil bath, and initial attempts to obtain **3o** under microwave irradiation were unsuccessful.

**Table 2:** Results for the solvent-free synthesis under microwave irradiation of (5*Z*) 2-amino-5-arylidene-1,3-thiazol-4(5*H*)-one derivatives **5a**–**q** by sulfur/nitrogen displacement reaction from (5*Z*) 5-arylidene-2-thioxo-1,3-thiazolidin-4-one derivatives **3** and cyclic secondary amines **4a**–**h**.

Compound 5		Starting Compound 3	Starting Compound 4	Reactions Conditions Used under Microwave *^a^*			Yield *^b^* of 5 (%)
				Temp. (°C)	Time (min.)	Power *^c^* (W)	
**5a**	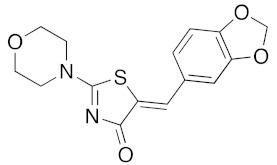	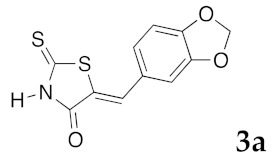	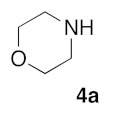	80	20	80	96
**5b**	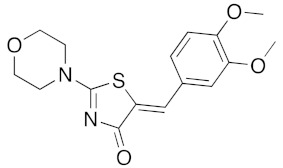	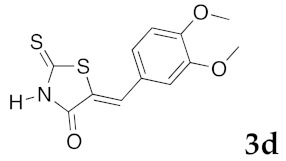	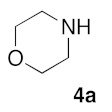	80	25	80	64
**5c**	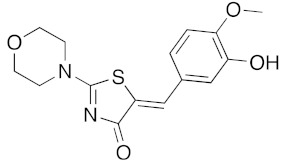	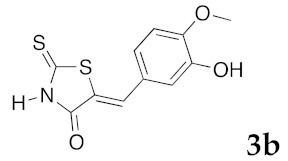	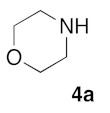	80	20	80	88
**5d**	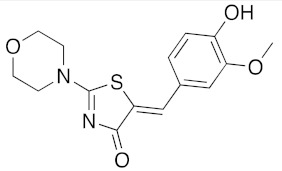	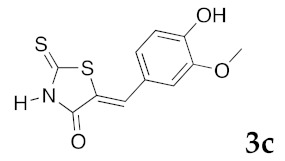	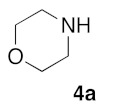	80	20	80	86
**5e**	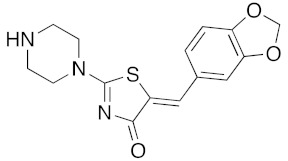	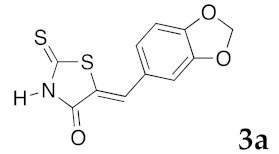	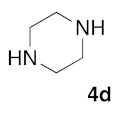	80	60	80	78
**5f**	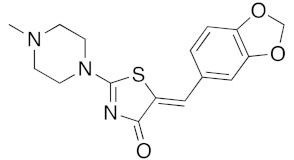	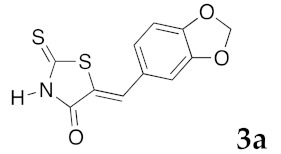	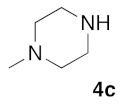	80	60	80	84
**5g**	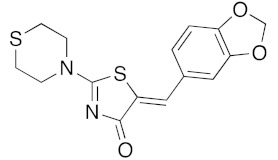	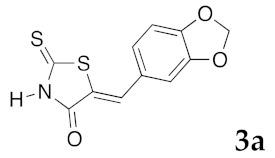	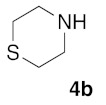	80	30	80	80
**5h**	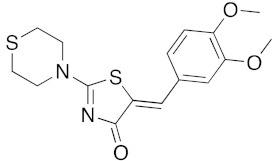	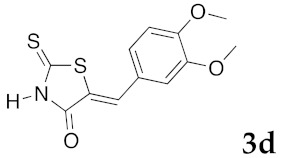	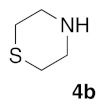	80	35	80	78
**5i**	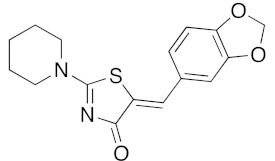	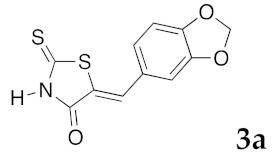	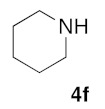	80	20	80	80
**5j**	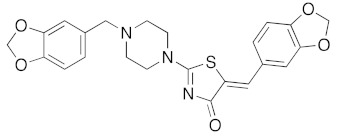	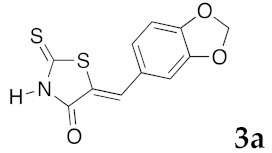	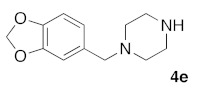	100	30	80	75
**5k**	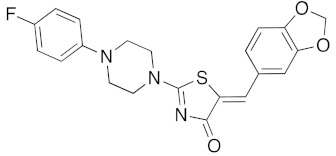	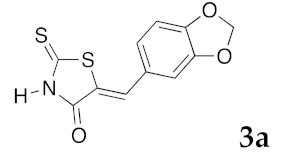	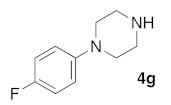	120	20	150	72
**5l**	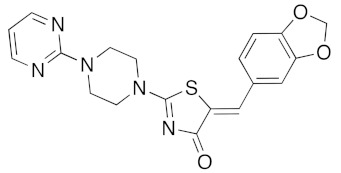	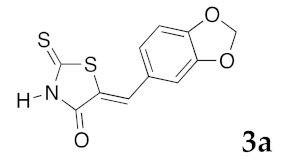	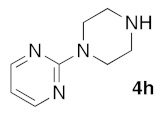	100	25	150	80
**5m**	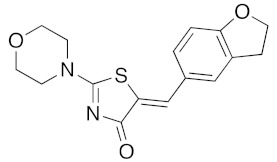	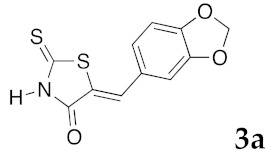	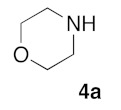	80	20	90	80
**5n**	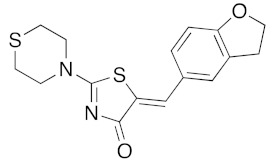	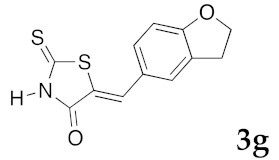	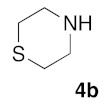	80	20	90	84
**5o**	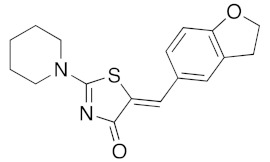	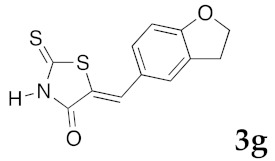	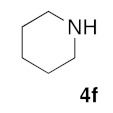	80	20	90	86
**5p**	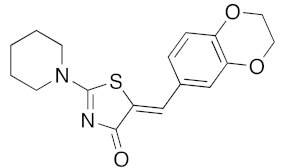	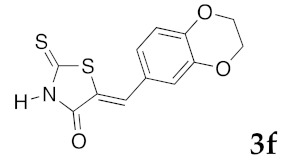	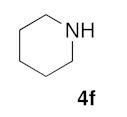	80	20	90	88
**5q**	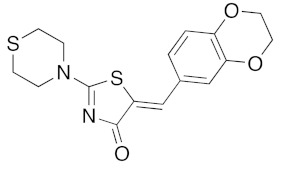	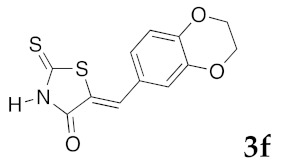	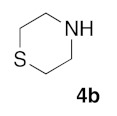	80	20	90	80

*^a^* Microwave irradiation (MWI) of the reaction mixture was realized in an open glass microwave reactor. *^b^* Isolated yield after work up, crystallization, and washing using appropriate solvent. *^c^* Synthewave^®^ 402 (Merck Eurolab Prolabo Fr) used as a microwave reactor (Power max.: 300 W).

**Table 3:** Results for the preparation under microwave irradiation of (5*Z*) 2-*N*-heteroarylamino-5-arylidene-1,3-thiazol-4(5*H*)-one derivatives **5r**–**x** from 2-*N*-heteroarylamino-thiazolidin-4-one **8a**–**e** and aromatic aldehydes **3a**–**c**.

Compound 5		Reaction Conditions Used under Microwave *^a^*				Yield *^c^* of 5 (%)
		Reagents	Reaction Temp. (°C)	Reaction Time (min.)	Power *^b^* (W)	
**5r**	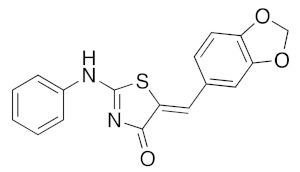	piperidine 0.1 eq AcOH 0.1 eq	150	20	200	34
**5s**	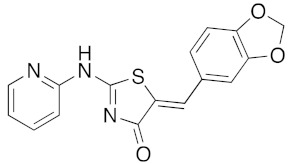	AcONa 0.1 eq AcOH 6.6 eq	120	20	100	90
**5t**	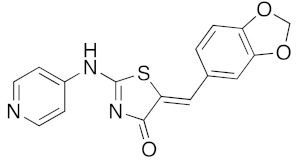	AcONa 0.1 eq AcOH 6.6 eq	120	40	100	43
**5u**	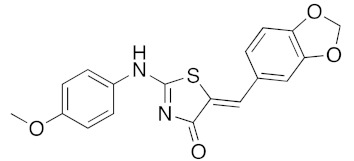	AcONa 0.1 eq AcOH 6.6 eq	120	20	100	90
**5v**	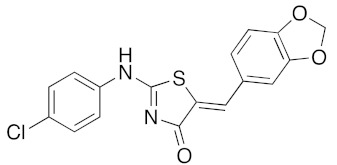	AcONa 0.1 eq AcOH 6.6 eq	120	20	100	79
**5w**	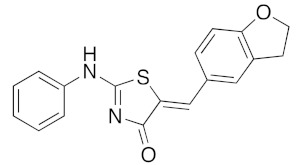	piperidine 0.1 eq AcOH 0.1 eq	150	20	200	64
**5x**	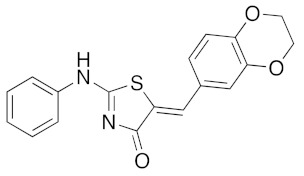	AcONa 0.1 eq AcOH 6.6 eq	120	20	100	98

*^a^* Microwave irradiation (MWI) of the reaction mixture was realized in a glass tube sealed with a snap cap (closed vessel). *^b^* Explorer^®^ 24 (CEM μWave France) used as a microwave reactor (Power max.: 300 W). *^c^* Isolated yield after work up, crystallization, and washing using appropriate solvent.

**Table 4:** Effects of the (5*Z*) 5-arylidene-2-thioxo-1,3-thiazolidin-4-one **3a**–**v** intermediates on the catalytic activity of four purified protein kinases *^a^*.

Compound	DYRK1A	CK1	CDK5/p25	GSK3α/β
**3a**	0.070	-	>10	8.5
**3b**	1.4	-	-	>10
**3c**	0.064	≥10	8	8
**3d**	>10	-	-	-
**3e**	0.028	>10	>10	>10
**3f**	0.056	-	-	≤10
**3g**	0.090	-	-	≥10
**3h**	0.35	-	-	1.6
**3i**	0.7	-	-	>10
**3j**	>10	-	-	>10
**3k**	>10	-	>10	>10
**3l**	15	-	-	>100
**3m**	6.5	-	-	>10
**3n**	>10	-	-	>10
**3o**	>10	-	-	>10
**3p**	>10	-	-	>10
**3q**	>10	-	-	>10
**3r**	>10	-	-	>10
**3s**	>10	-	-	>10
**3t**	>10	-	-	>10
**3u**	0.16	-	-	>10
**3v**	0.70	-	-	>10
Harmine *^b^*	0.029	1.5	>10	>10
(*R*)-Roscovitine *^b^*	11.0	2.3	0.2	130

*^a^* Compounds were tested at various concentrations on each kinase as described in Experimental Section. IC_50_ values, calculated from the dose-response curves, are reported in μM. -, inactive at the highest concentration tested (10 μM); >10, inhibitory, but IC_50_ > 10 μM. *^b^* IC_50_ values; see refs. [39,40].

**Table 5:** Effects of the (5*Z*) 2-amino-5-arylidene-1,3-thiazol-4(5*H*)-one derivatives **5a**–**x** on the catalytic activity of four purified protein kinases *^a^*.

Compound	DYRK1A	CK1	CDK5/p25	GSK3α/β
**5a**	0.63	-	-	7.4
**5b**	>10	-	-	-
**5c**	-	-	-	-
**5d**	>10	-	-	-
**5e**	1.8	-	-	≤8.8
**5f**	2.3	-	-	-
**5g**	0.4	-	-	≥10
**5h**	-	-	-	-
**5i**	0.053	-	-	7.4
**5j**	≥10	-	-	-
**5k**	1.6	-	-	-
**5l**	1.8	-	-	>10
**5m**	0.6	>10	-	≥10
**5n**	0.070	>10	-	5.3
**5o**	0.090	≥10	-	4.7
**5p**	0.94	≥10	-	>10
**5q**	0.17	-	-	3.7
**5r**	0.61	-	-	-
**5s**	0.033	-	-	≤10
**5t**	>10	-	-	>10
**5u**	0.9	-	-	>10
**5v**	9.0	>10	>10	>10
**5w**	0.12	-	-	-
**5x**	0.27	-	-	-
Harmine	0.029	1.5	>10	>10
*(R)*-Roscovitine	11	2.3	0.2	130

*^a^* Compounds were tested at various concentrations on each kinase as described in Experimental Section. IC_50_ values, calculated from the dose-response curves, are reported in μM. -, inactive at the highest concentration tested (10 μM); >10, inhibitory, but IC_50_ > 10 μM.

**Table 6:** Effects of the (5*Z*) 2-amino-5-arylidene-1,3-thiazol-4(5*H*)-one derivatives **5r**–**x** *^a^* and (5*Z*) 5-arylidene-2-thioxo-1,3-thiazolidin-4-one **3a**–**c**, **3f**,**g** derivatives *^b^* on six representative tumoral cell lines.

Compound	Cell Lines IC_50_ (μM)						
	Huh7 D12	Caco2	MDA-MB231	HCT116	PC3	NCI-H727	Fibroblasts
**5**(**r**–**x**)	>25	>25	>25	>25	>25	>25	>25
**3a**	>25	>25	>25	>25	>25	>25	>25
**3b**	>25	>25	>25	>25	>25	>25	>25
**3c**	>25	>25	>25	12	>25	>25	>25
**3f**	>25	>25	>25	>25	>25	>25	>25
**3g**	>25	>25	>25	>25	>25	>25	>25
Roscovitine	10	8	15	6	20	>25	>25
DMSO	>25	>25	>25	>25	>25	>25	>25

*^a^* The other derivatives **5a**–**q** have not been tested, or some of them are not soluble in the conditions for the preparation of the biological test. *^b^* The other derivatives **3d**,**e** and **3h**–**v** have not been tested, or some of them are not soluble in the conditions for the preparation of the biological test.

## Data Availability

The data presented in this study are available on request from the corresponding author.

## Supplementary Materials

The following are available online at https://www.mdpi.com/article/10.3390/ph14111086/s1, Supporting information.
