# Supplementary material for: Design and Microwave Synthesis of New (5Z) 5-Arylidene-2-thioxo-1,3-thiazolinidin-4-one and (5Z) 2-Amino-5-arylidene-1,3-thiazol-4(5H)-one as New Inhibitors of Protein Kinase DYRK1A
Source: Pharmaceuticals (Basel). 2021 Oct 27;14(11):1086. doi: 10.3390/ph14111086 (PMC8623179; doi:10.3390/ph14111086)

pharmaceuticals

MDPI

Supporting Information

Design and microwave synthesis of new (5*Z*) 5-arylidene-2-thioxo-1,3-thiazolidin-4-one and (5*Z*) 2-amino-5-arylidene-1,3-thiazol-4(5*H*)-one as new inhibitors of protein kinase DYRK1A.

Khadidja Bourhala [1], Solène Guihéneuf [1], Emmanuelle Limanton [1,6], Ludovic Paquin [1,6], Rémy Le Guével [2], Thierry Charlier [2,3], Mustapha Rahmouni [4], Emilie Durieu[5,7], Olivier Lozach[7] , François Carreaux [1], Laurent Meijer [5] and Jean-Pierre Bazureau [1,6,*]

1 Institut des Sciences Chimiques de Rennes ISCR UMR CNRS 6226, Université de Rennes 1, Bât. 10A, Campus de Beaulieu, CS 74205, 263 Avenue du Général Leclerc, 35042 Rennes Cedex, France
2 ImPACcell platform, SFR Biosit, Université de Rennes 1, Bât. 8, Campus Villejean, 2 Avenue du Prof. Léon Bernard, CS 34317, 35043 Rennes Cedex, France
3 Institut de Recherche en Santé, Environnement et Travail, IRSET Inserm U1085, Université de Rennes 1, 9 Avenue du Prof. Léon Bernard, 35000 Rennes, France
4 Université Ibn Khaldoun, Laboratoire de Synthèse et Catalyse, 14000 Tiaret, Algérie
5 Perha Pharmaceuticals & ManRos Therapeutics, "From Sea to Pharmacy", Hôtel de Recherche, 29680 Roscoff, France
6 S2Wave platform, ScanMAT UMS 2001 CNRS, Université de Rennes 1, Bât. 10A, Campus de Beaulieu, CS 74205, 263 Avenue du Général Leclerc, 35042 Rennes Cedex, France
7 Protein Phosphorylation & Human Disease group, Station Biologique, 29680 Roscoff, France

* Correspondence: jean-pierre.bazureau@univ-rennes1.fr; Tel.: +33 223 236 603

	Pages
**Section 1:** ^{1}H and ^{13}C NMR spectra of compounds **3**	2-22
**Section 2:** ^{1}H and ^{13}C NMR spectra of compounds **5**	23-39

Section 1: ^{1}H and ^{13}C NMR spectra of compounds **3.**

^{1}H NMR (300 MHz, DMSO-d_6) of

(5*Z*)-5-{[6-(1,3-benzodioxol-5-yl)pyridin-2-yl]methylene}-2-thioxo-1,3-thiazolidin-4-one (**3h**).

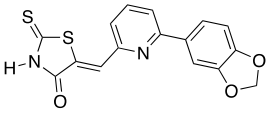

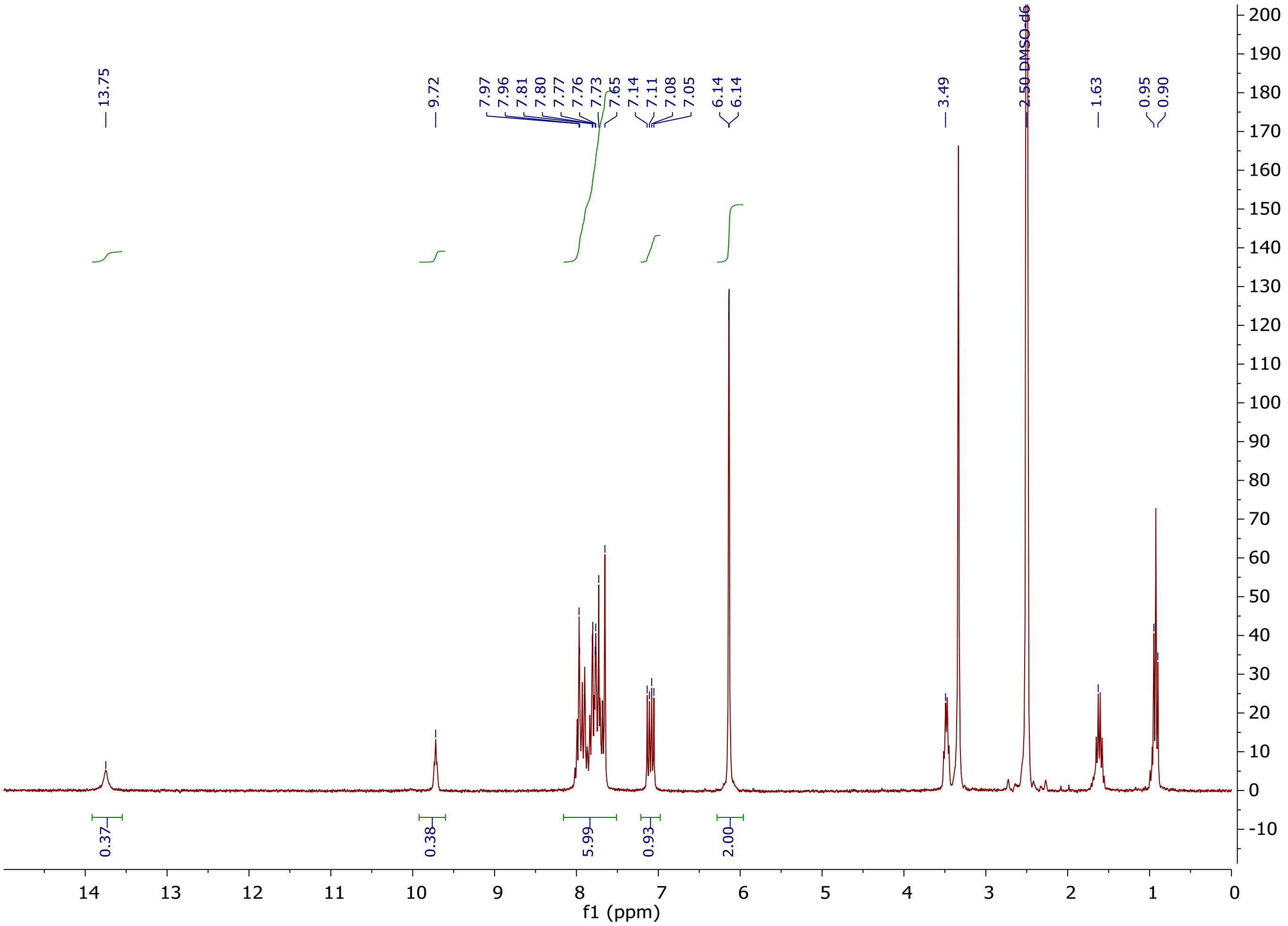

^{1}H NMR (300 MHz, DMSO-d_6) of (5*Z*)-5-(chroman-6-yl)methylene-2-thioxo-1,3-thiazolidine-4-one (**3m**).

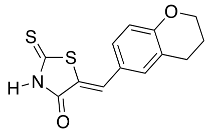

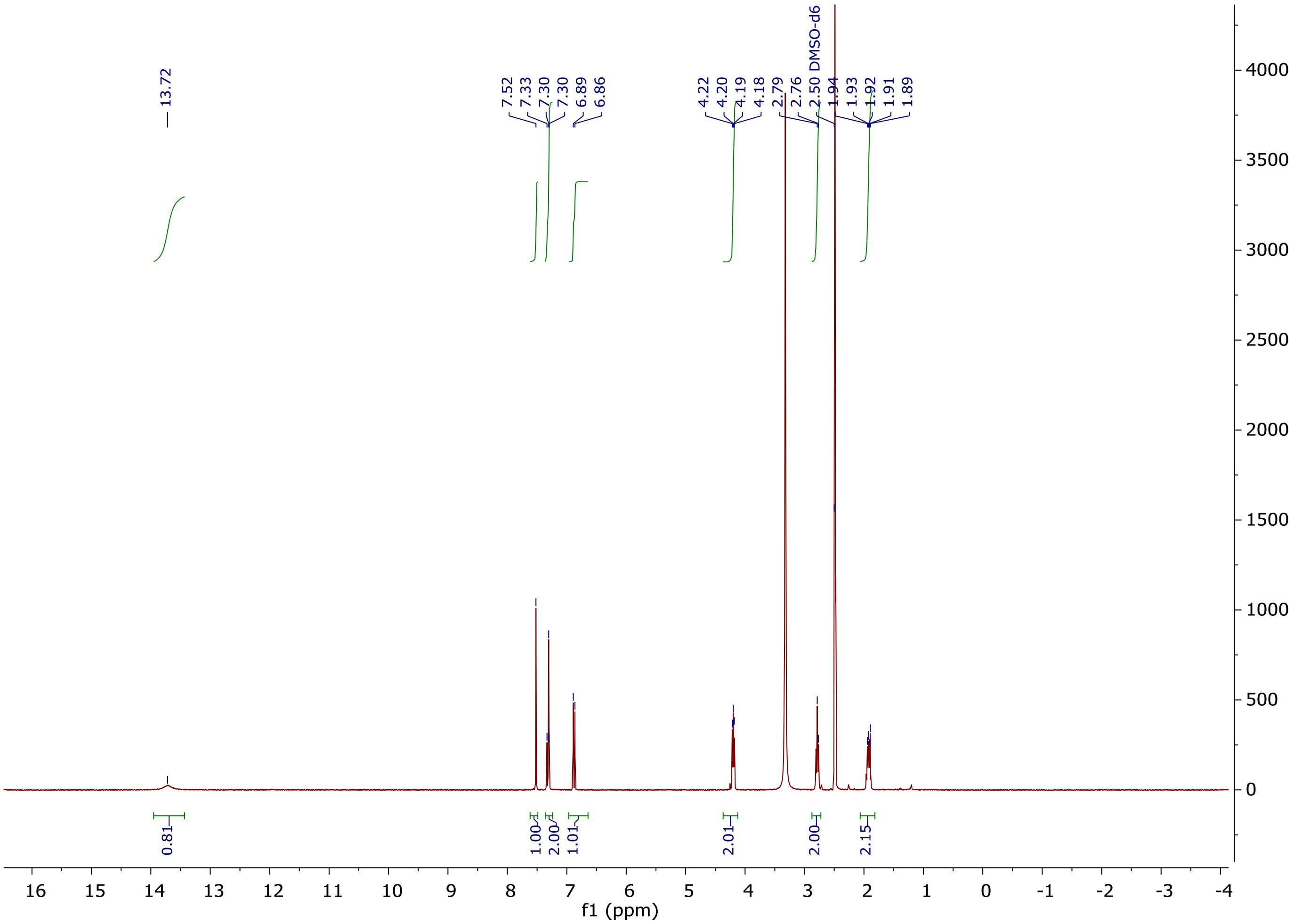

^{13}C NMR (75 MHz, DMSO-d_6) of (5*Z*)-5-(chroman-6-yl)methylene-2-thioxo-1,3-thiazolidine-4-one (**3m**).

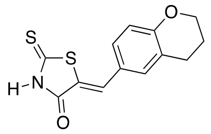

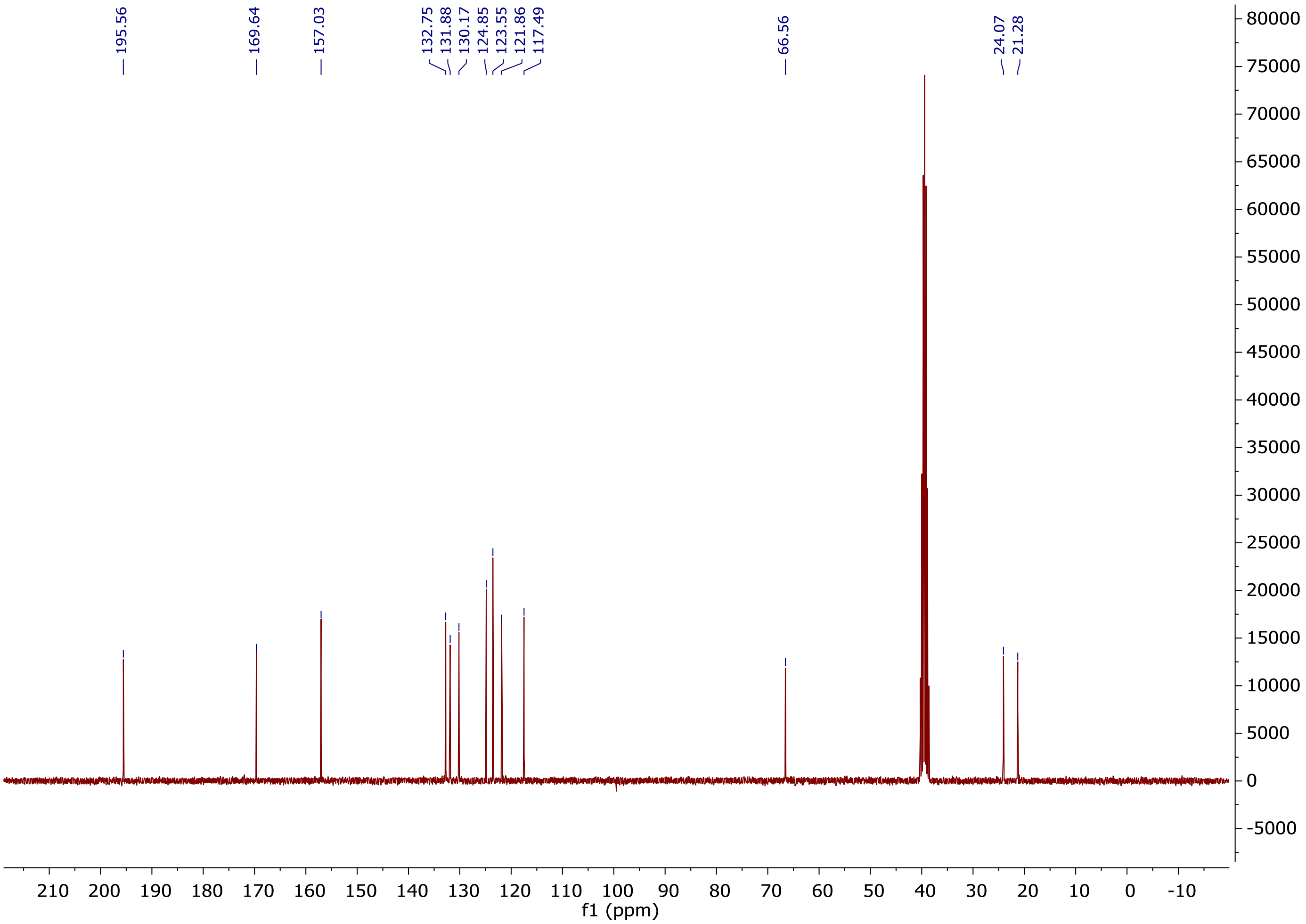

^{1}H NMR (300 MHz, DMSO-d_6) of
(5*Z*)-5-(3,4-dihydro-2*H*-1,5-benzodioxepine-7-yl)methylene-2-thioxo-1,3-thiazolidine-4-one (**3n**).

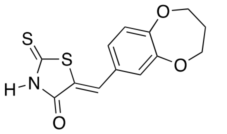

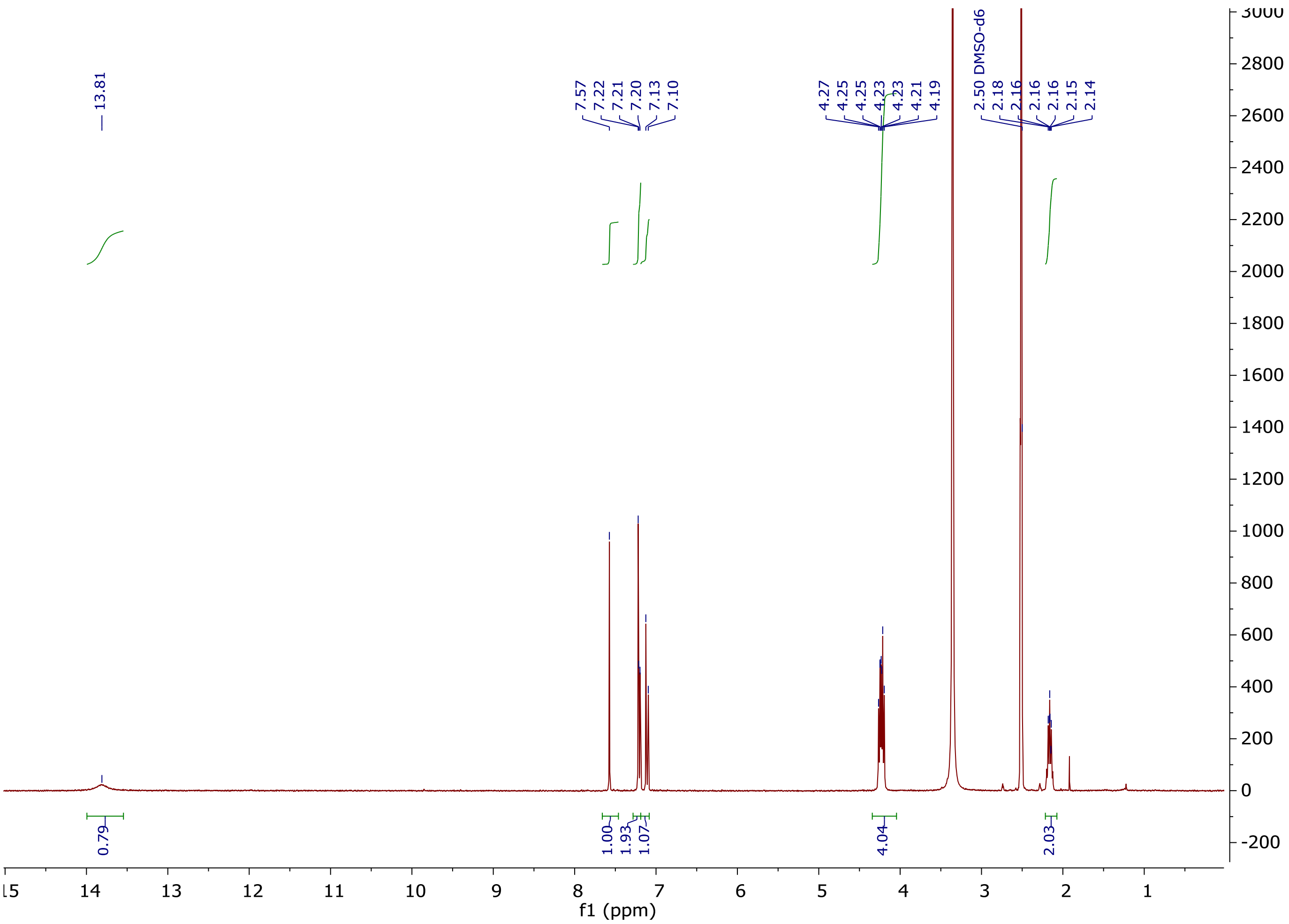

^{13}C NMR (75 MHz, DMSO-d_6) of

(5*Z*)-5-(3,4-dihydro-2*H*-1,5-benzodioxepine-7-yl)methylene-2-thioxo-1,3-thiazolidine-4-one (**3n**).

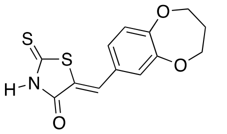

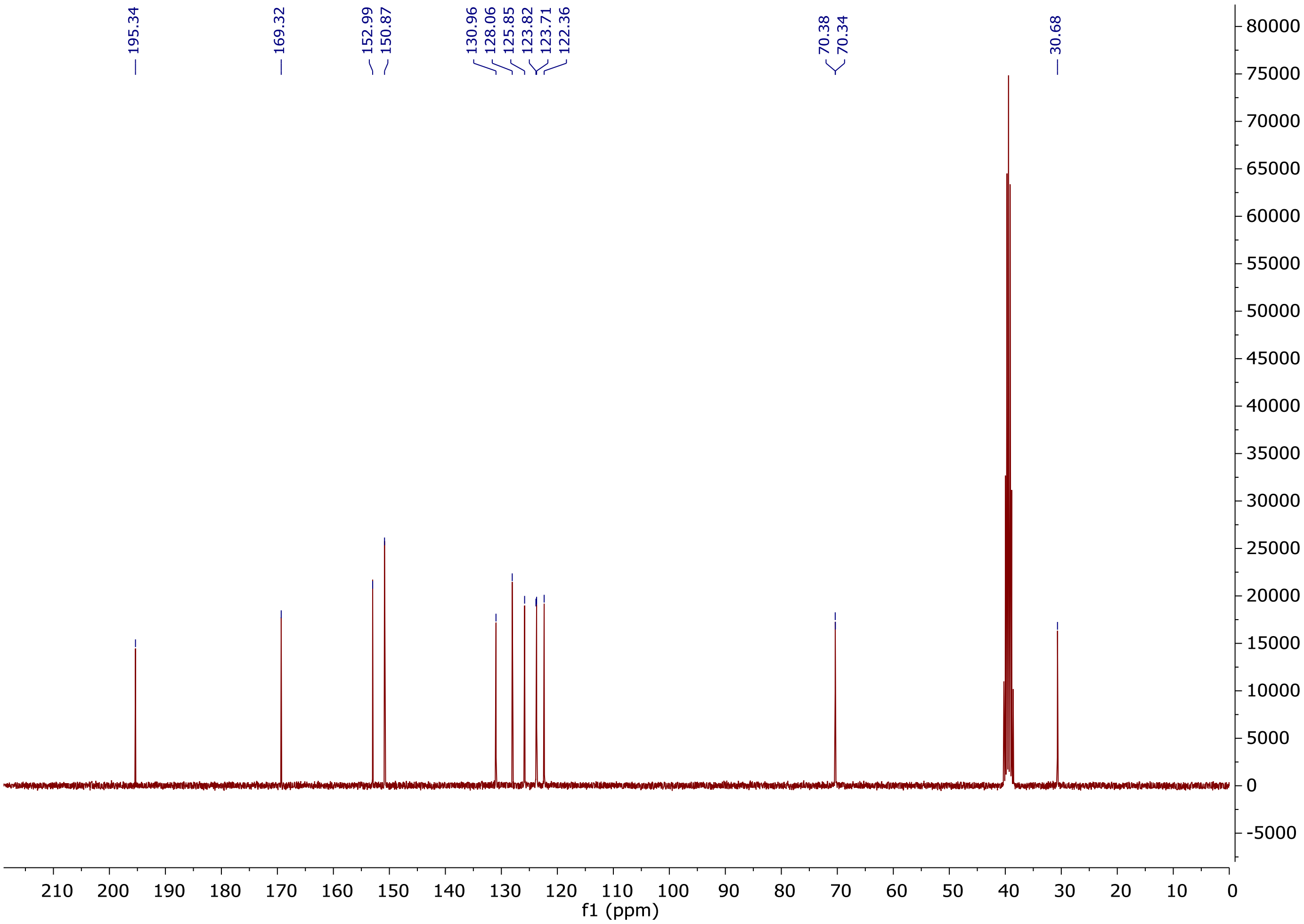

^{1}H NMR (300 MHz, DMSO-d_6) of (5*Z*)-3-amino-5-benzo[1,3]dioxol-5-ylmethylene-2-thioxo-thiazolidin-4-one (**3o**).

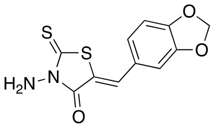

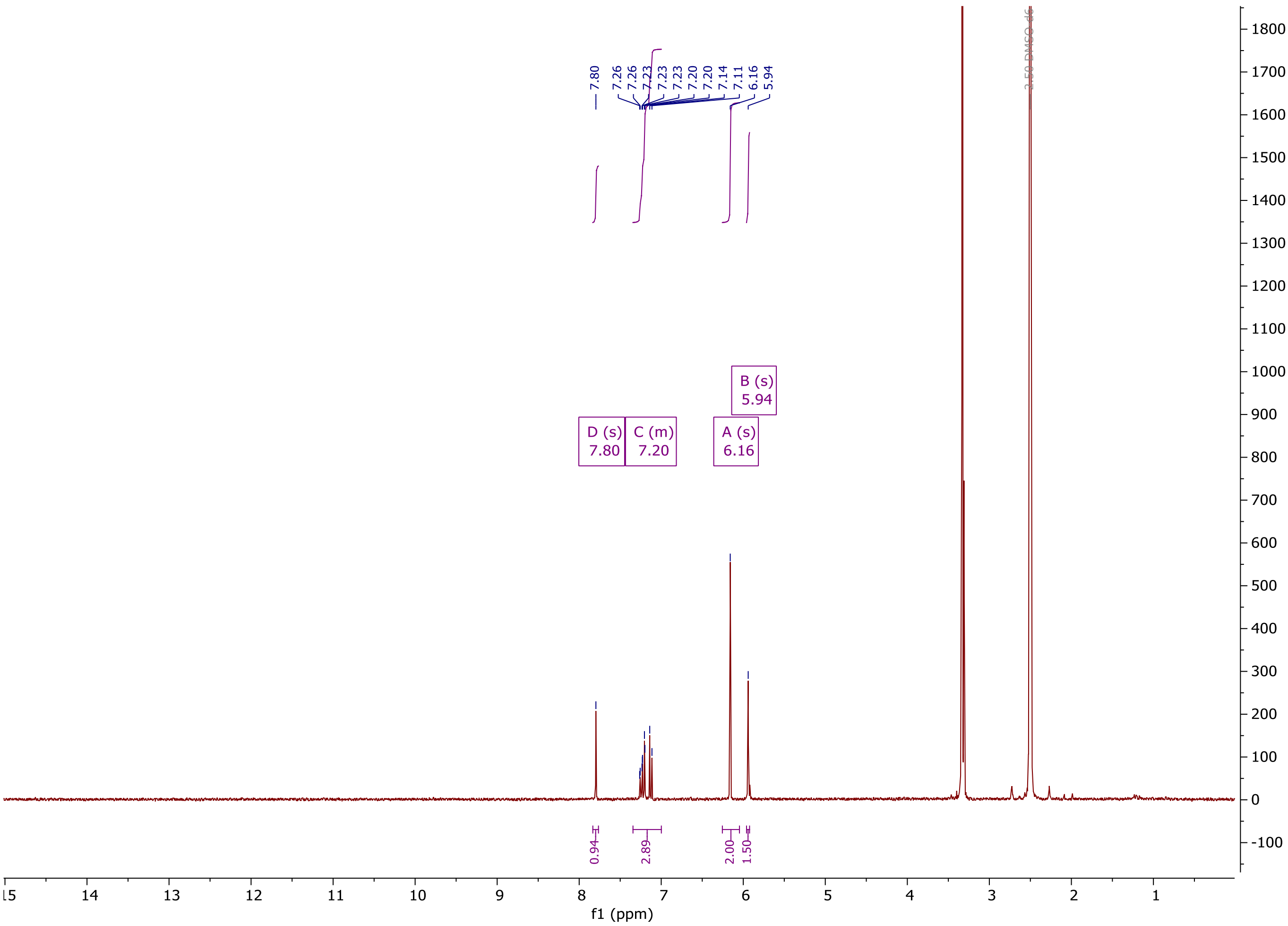

^{13}C NMR (75 MHz, DMSO-*d*$_6$) of (5*Z*)-3-amino-5-benzo[1,3]dioxol-5-ylmethylene-2-thioxo-thiazolidin-4-one (**3o**).

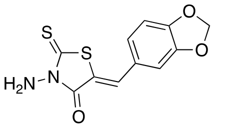

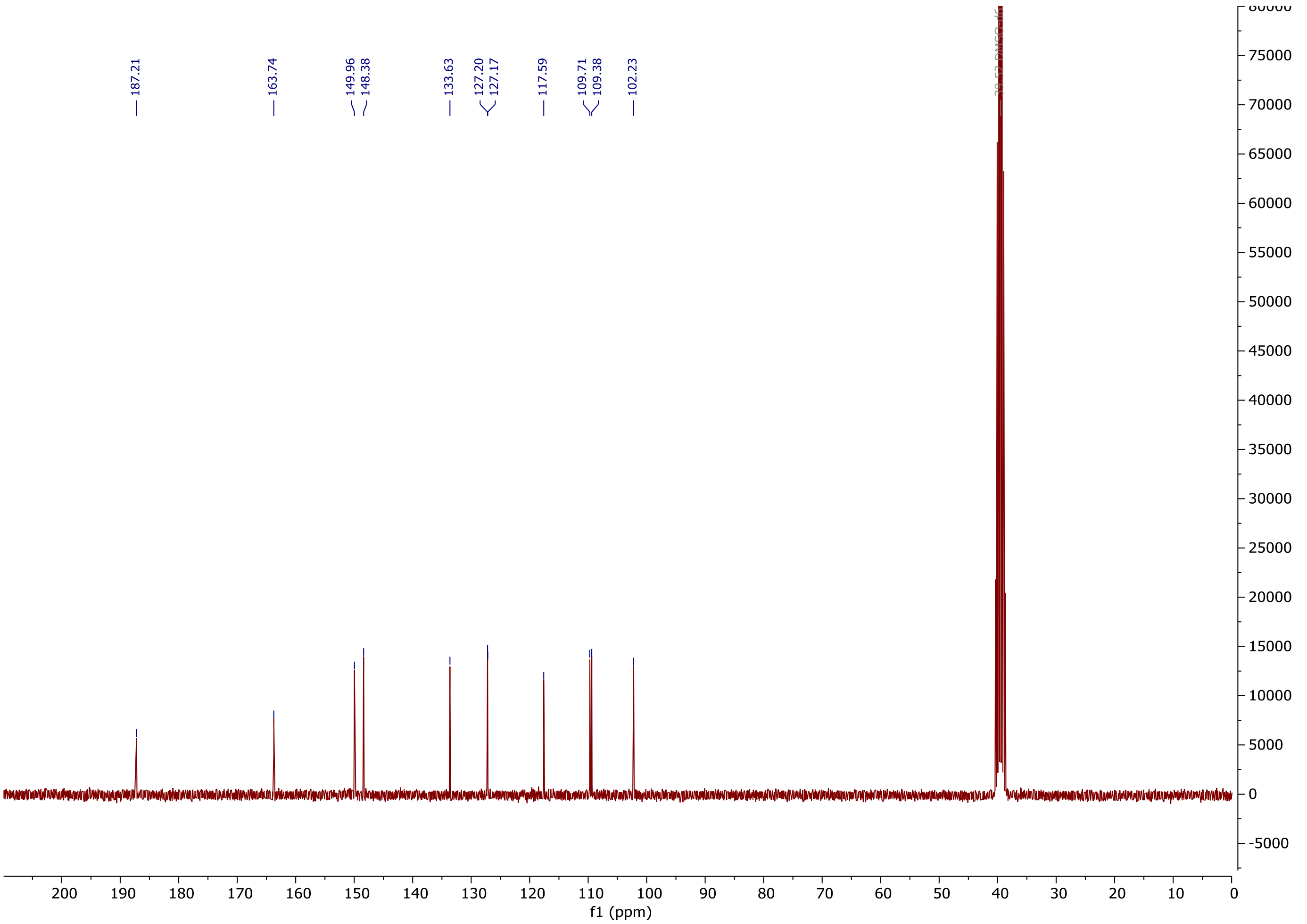

^{1}H NMR (300 MHz, DMSO-d_6) of

(5*Z*)-*N*-(5-benzo[1,3]dioxol-5-ylmethylene-4-oxo-2-thioxo-thiazolidin-3-yl)-benzamide (**3p**).

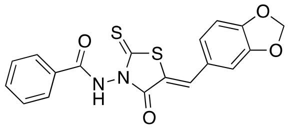

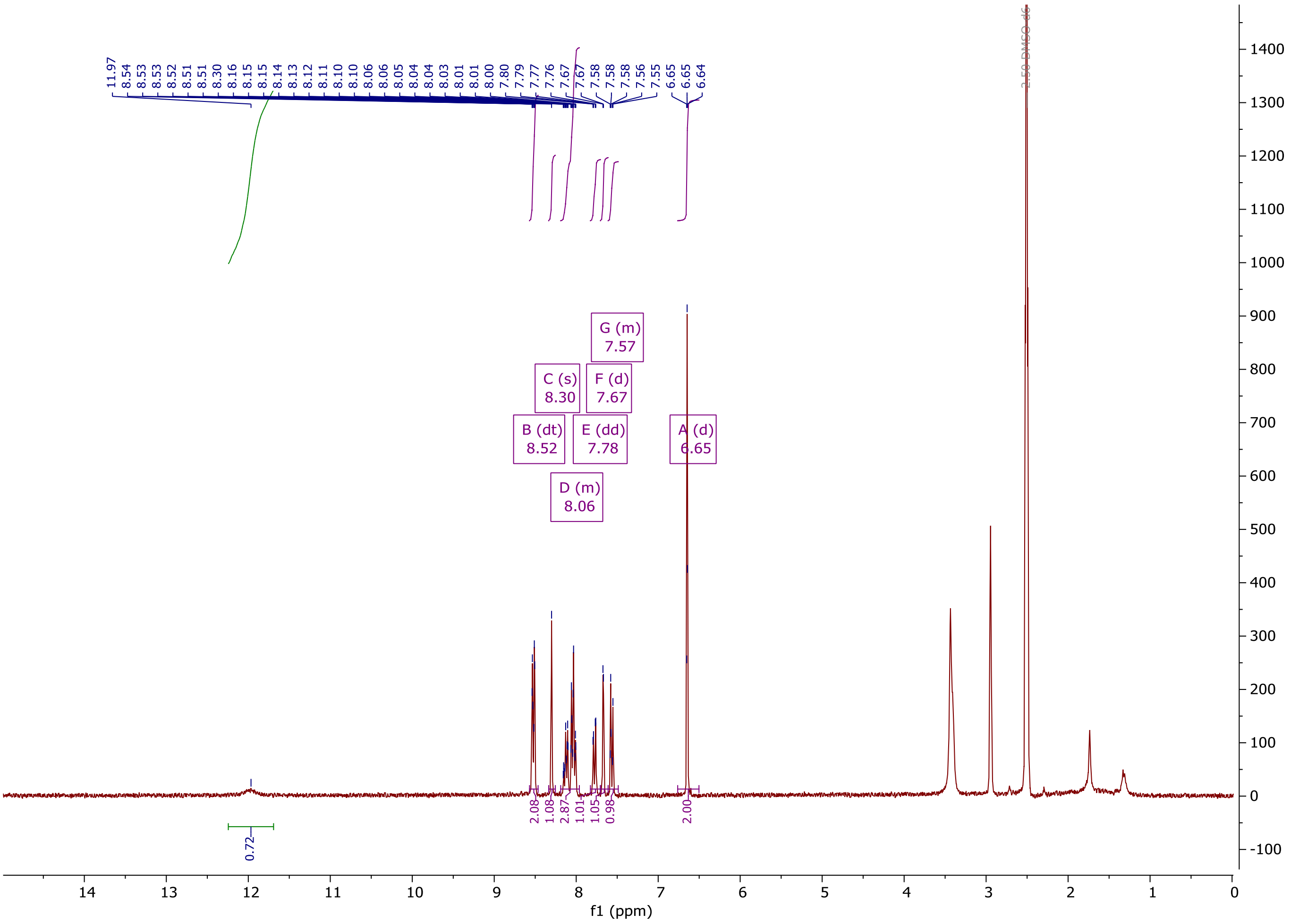

^{13}C NMR (75 MHz, DMSO-d_6) of

(5*Z*)-*N*-(5-benzo[1,3]dioxol-5-ylmethylene-4-oxo-2-thioxo-thiazolidin-3-yl)-benzamide (**3p**).

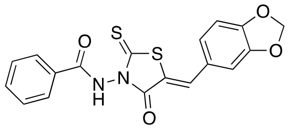

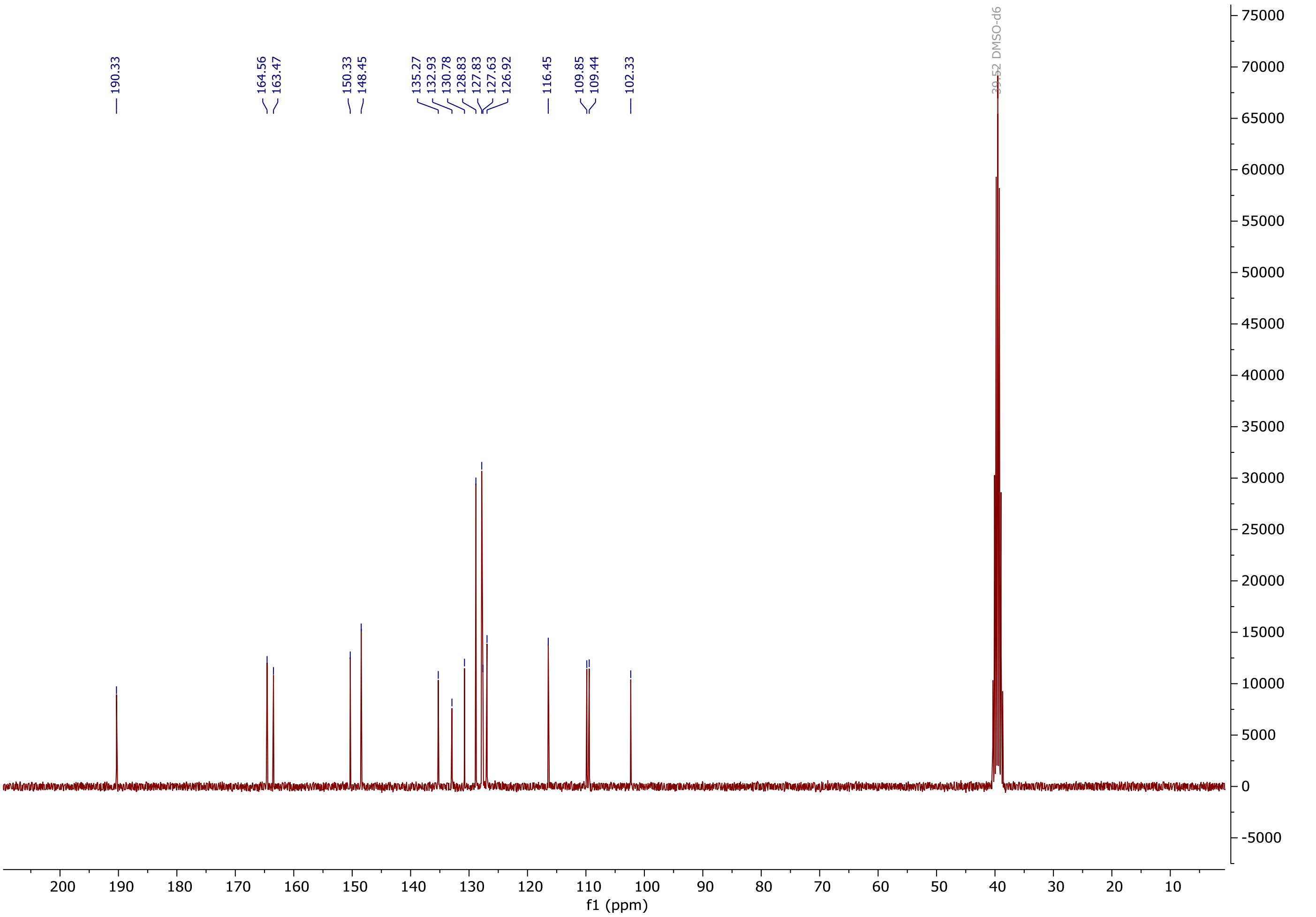

^{1}H NMR (300 MHz, DMSO-d_6) of

(5*Z*)-*N*-(5-benzo[1,3]dioxol-5-ylmethylene-4-oxo-2-thioxo-thiazolidin-3-yl)-2-phenyl-acetamide (**3q**).

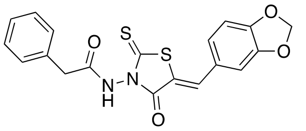

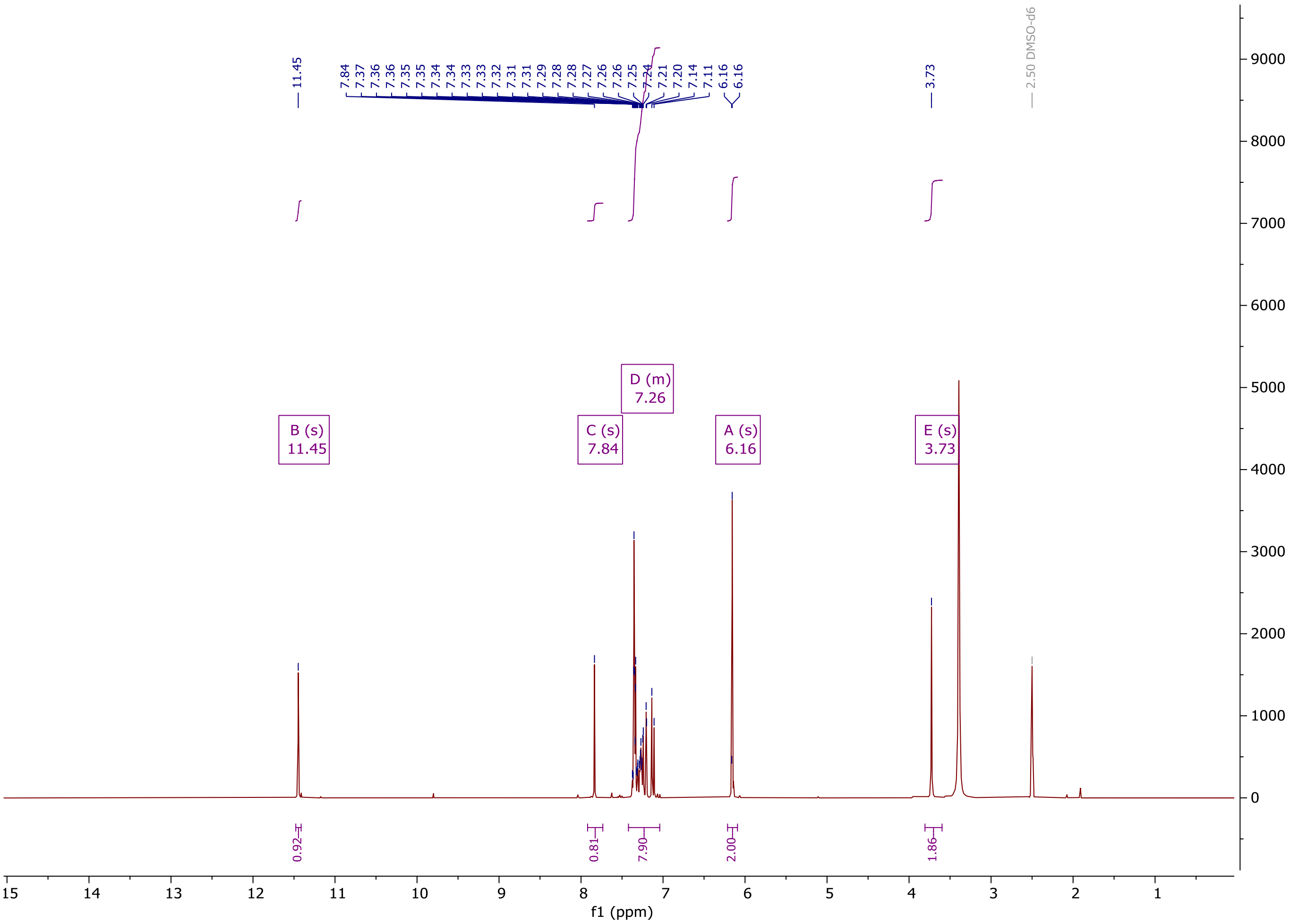

^{13}C NMR (75 MHz, DMSO-d_6) of
(5*Z*)-*N*-(5-benzo[1,3]dioxol-5-ylmethylene-4-oxo-2-thioxo-thiazolidin-3-yl)-2-phenyl-acetamide (**3q**).

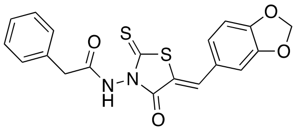

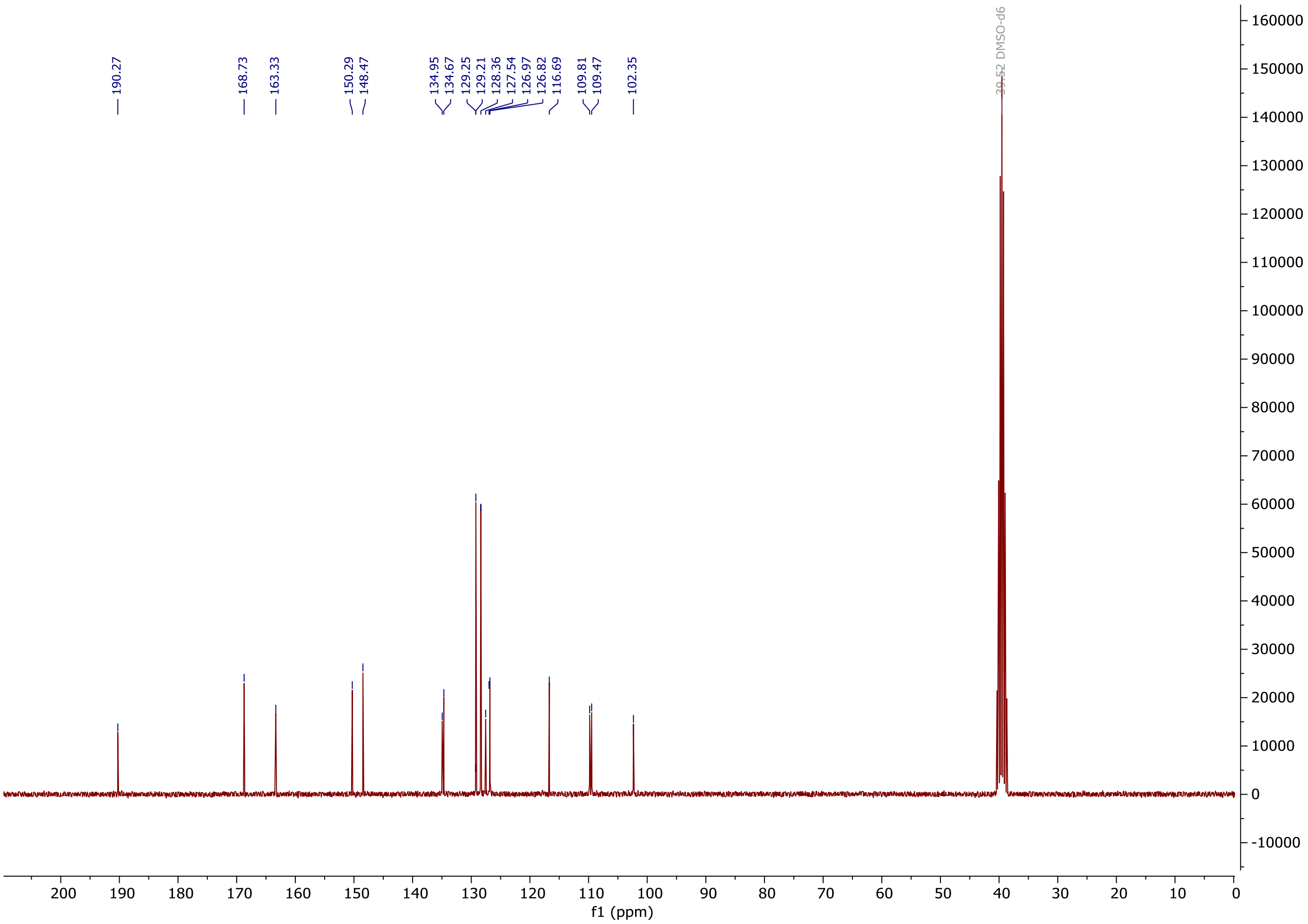

^{1}H NMR (300 MHz, DMSO-d_6) of

(5*Z*)-N-(5-benzo[1,3]dioxol-5-ylmethylene-4-oxo-2-thioxo-thiazolidin-3-yl)-3-phenyl-propionamide (**3r**).

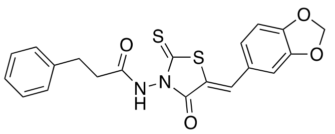

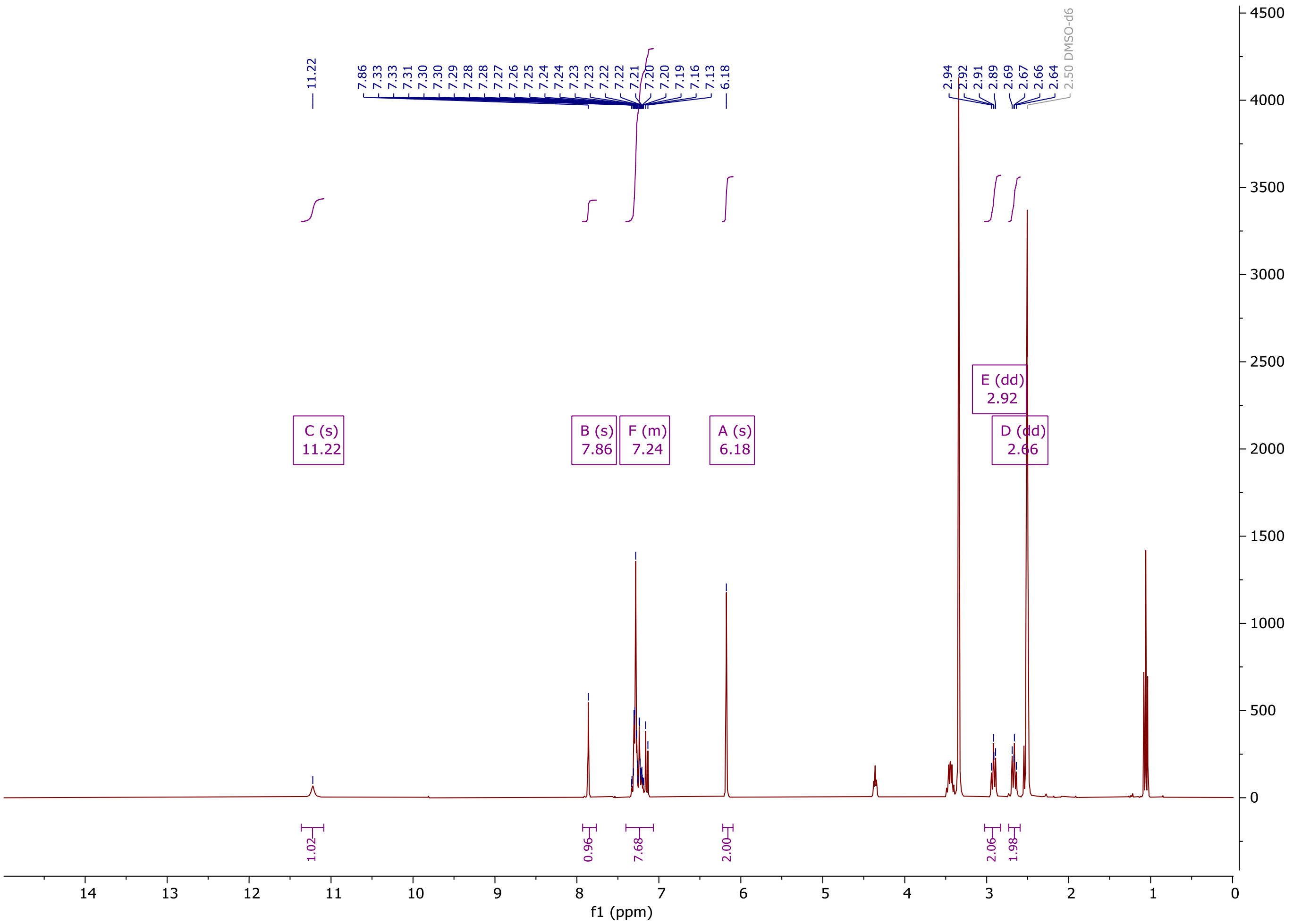

^{13}C NMR (75 MHz, DMSO-d_6) of

(5*Z*)-N-(5-benzo[1,3]dioxol-5-ylmethylene-4-oxo-2-thioxo-thiazolidin-3-yl)-3-phenyl-propionamide (**3r**).

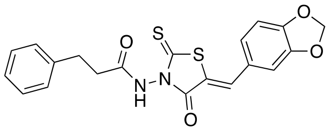

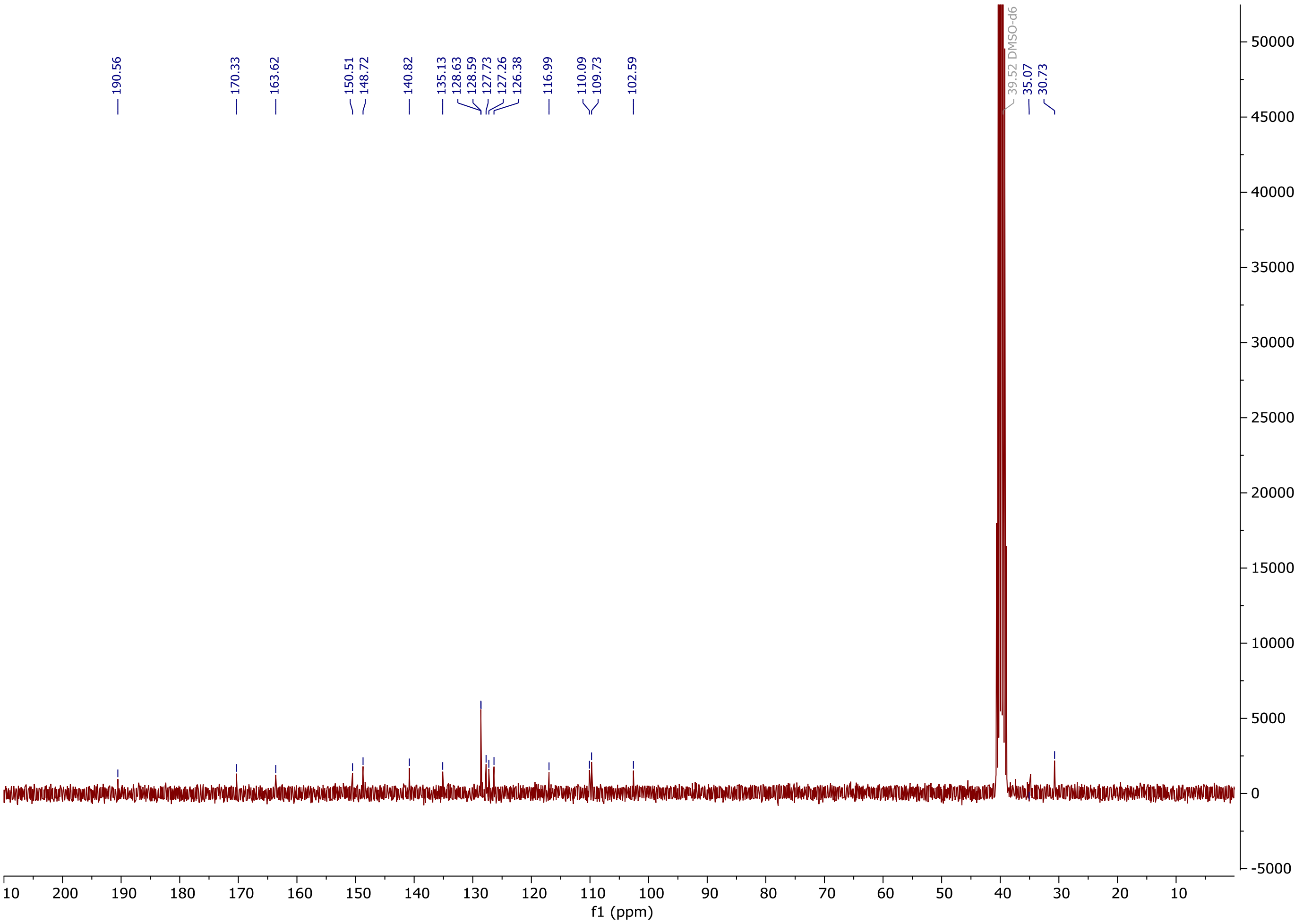

^{1}H NMR (300 MHz, DMSO-d_6) of

(5*Z*)-5-benzo[1,3]dioxol-5-ylmethylene-3-[2-(4-methoxy-phenyl)-2-oxo-ethyl]-2-thioxo-thiazolidin-4-one (**3s**).

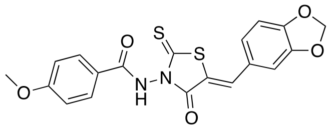

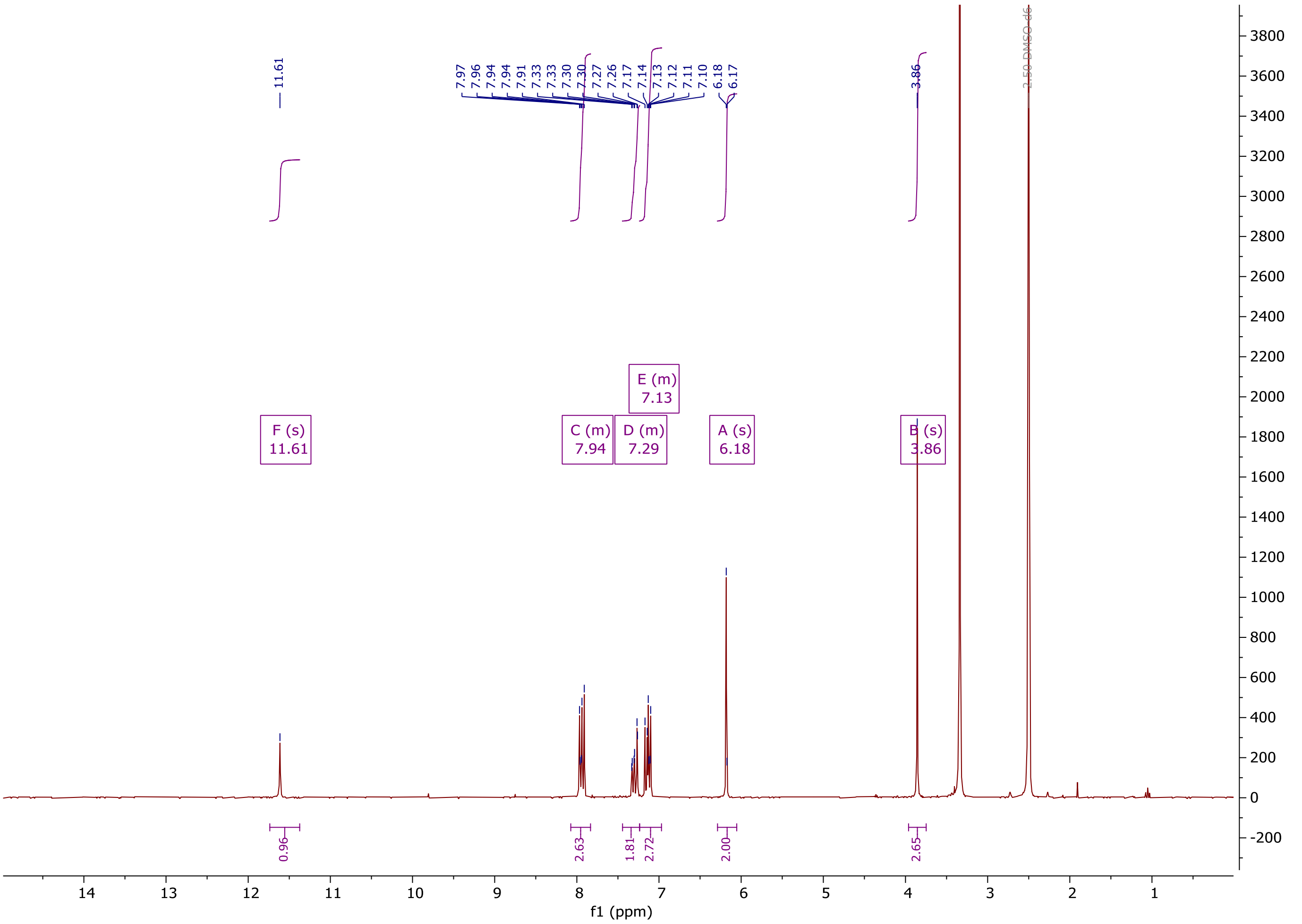

^{13}C NMR (75 MHz, DMSO-d_6) of

(5*Z*)-5-benzo[1,3]dioxol-5-ylmethylene-3-[2-(4-methoxy-phenyl)-2-oxo-ethyl]-2-thioxo-thiazolidin-4-one (**3s**).

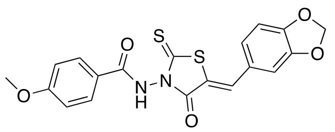

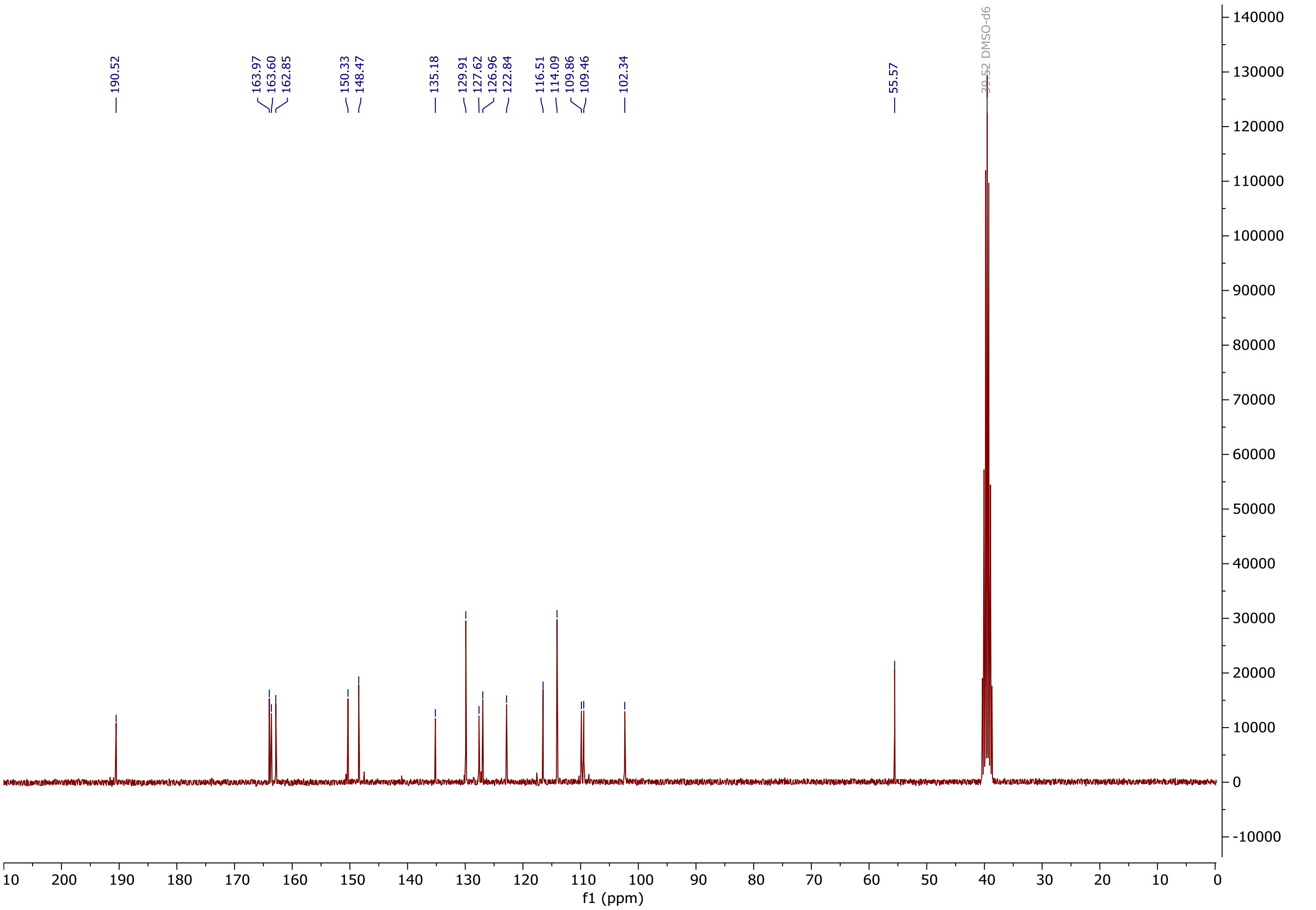

^{1}H NMR (300 MHz, DMSO-d_6) of

(5*Z*)-*N*-(5-benzo[1,3]dioxol-5-ylmethylene-4-oxo-2-thioxo-thiazolidin-3-yl)-benzenesulfonamide (**3t**).

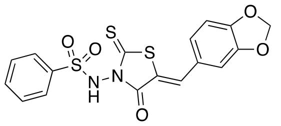

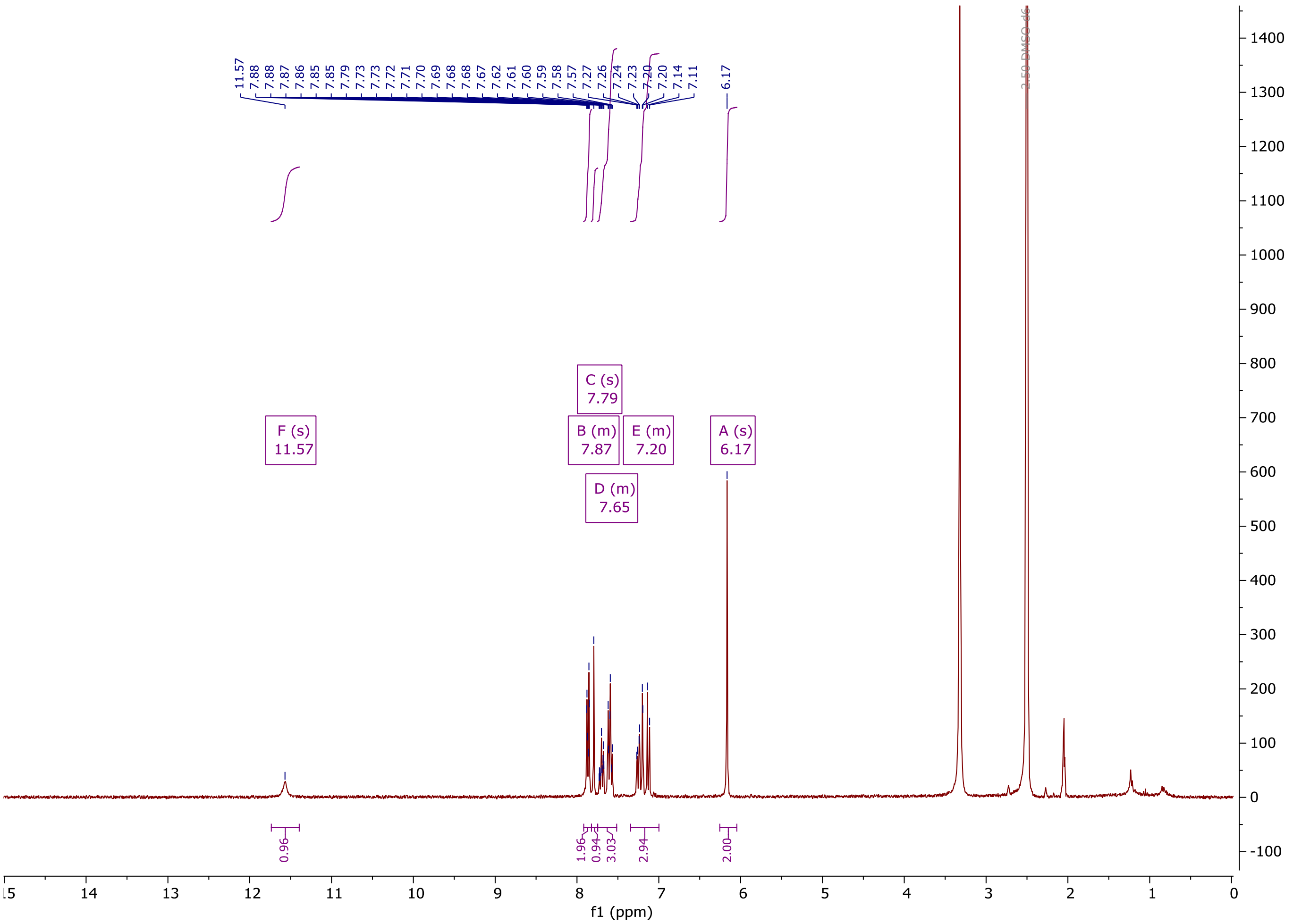

^{13}C NMR (75 MHz, DMSO-d_6) of

(5*Z*)-*N*-(5-benzo[1,3]dioxol-5-ylmethylene-4-oxo-2-thioxo-thiazolidin-3-yl)-benzenesulfonamide (**3t**).

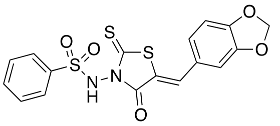

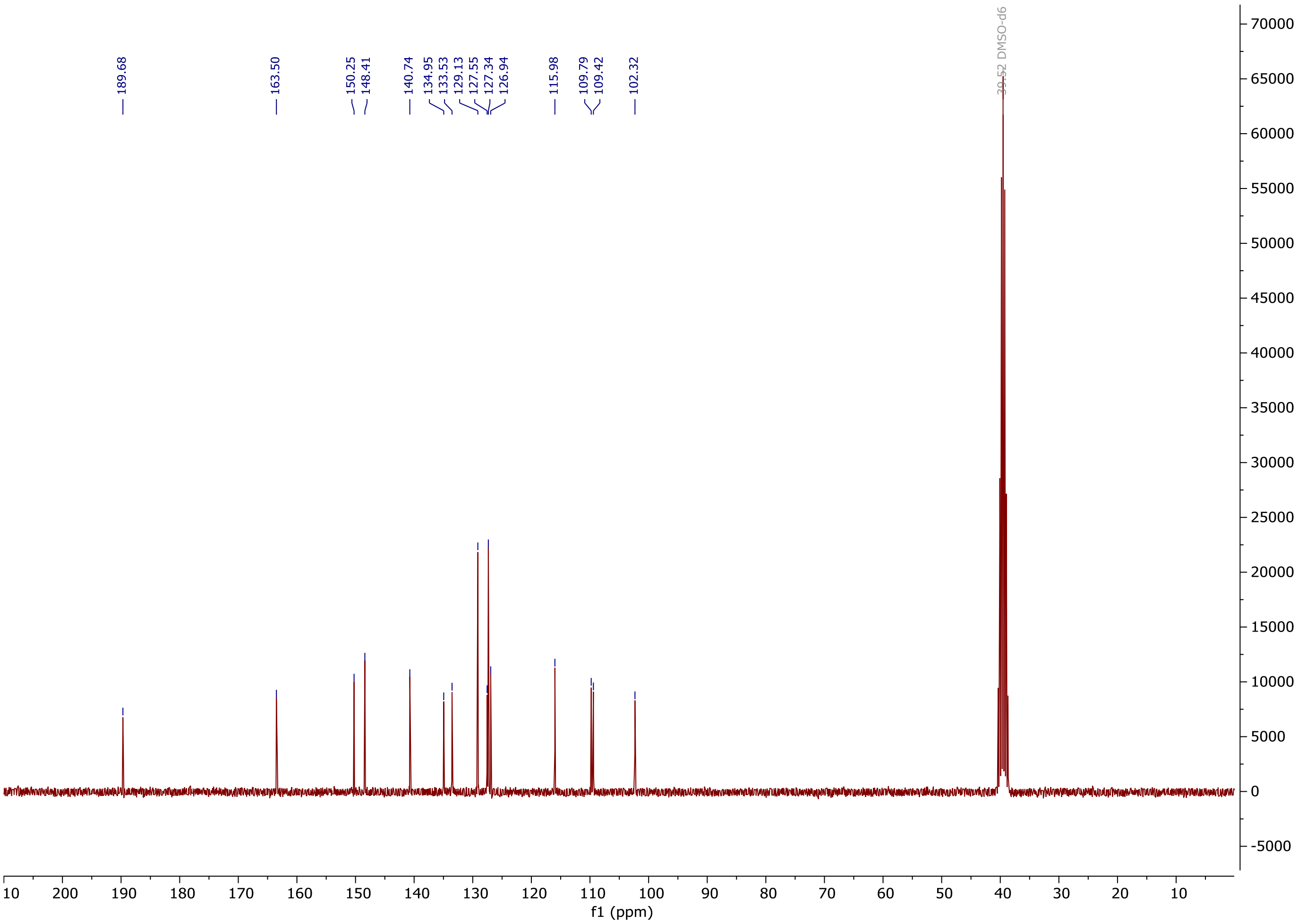

^{1}H NMR (300 MHz, DMSO-d_6) of (5*Z*)-(5-benzo[1,3]dioxol-5-ylmethylene-4-oxo-2-thioxo-thiazolidin-3-yl)-acetic acid (**3u**).

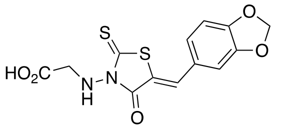

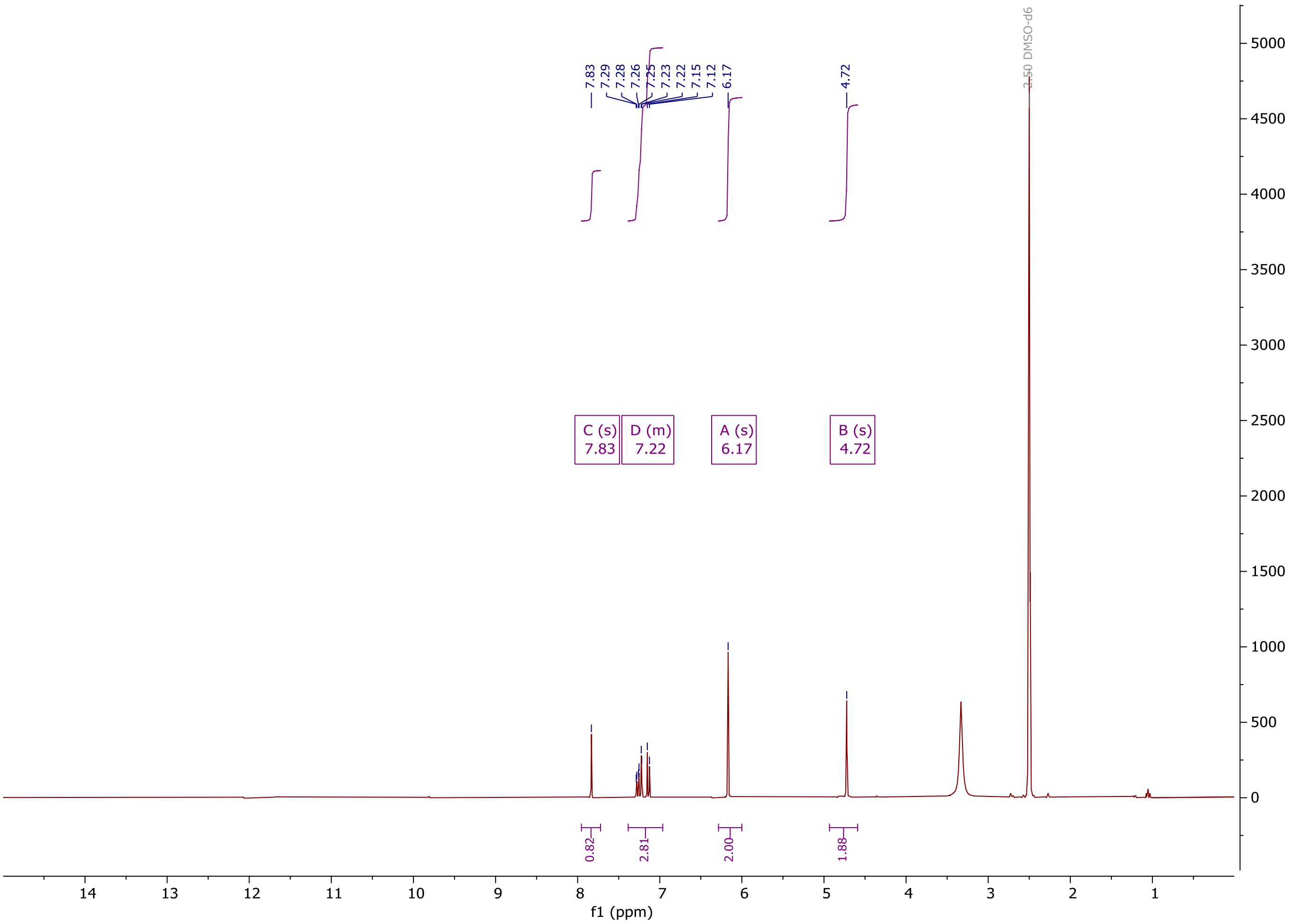

^{13}C NMR (75 MHz, DMSO-d_6) of (5*Z*)-(5-benzo[1,3]dioxol-5-ylmethylene-4-oxo-2-thioxo-thiazolidin-3-yl)-acetic acid (**3u**).

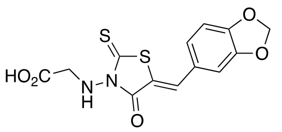

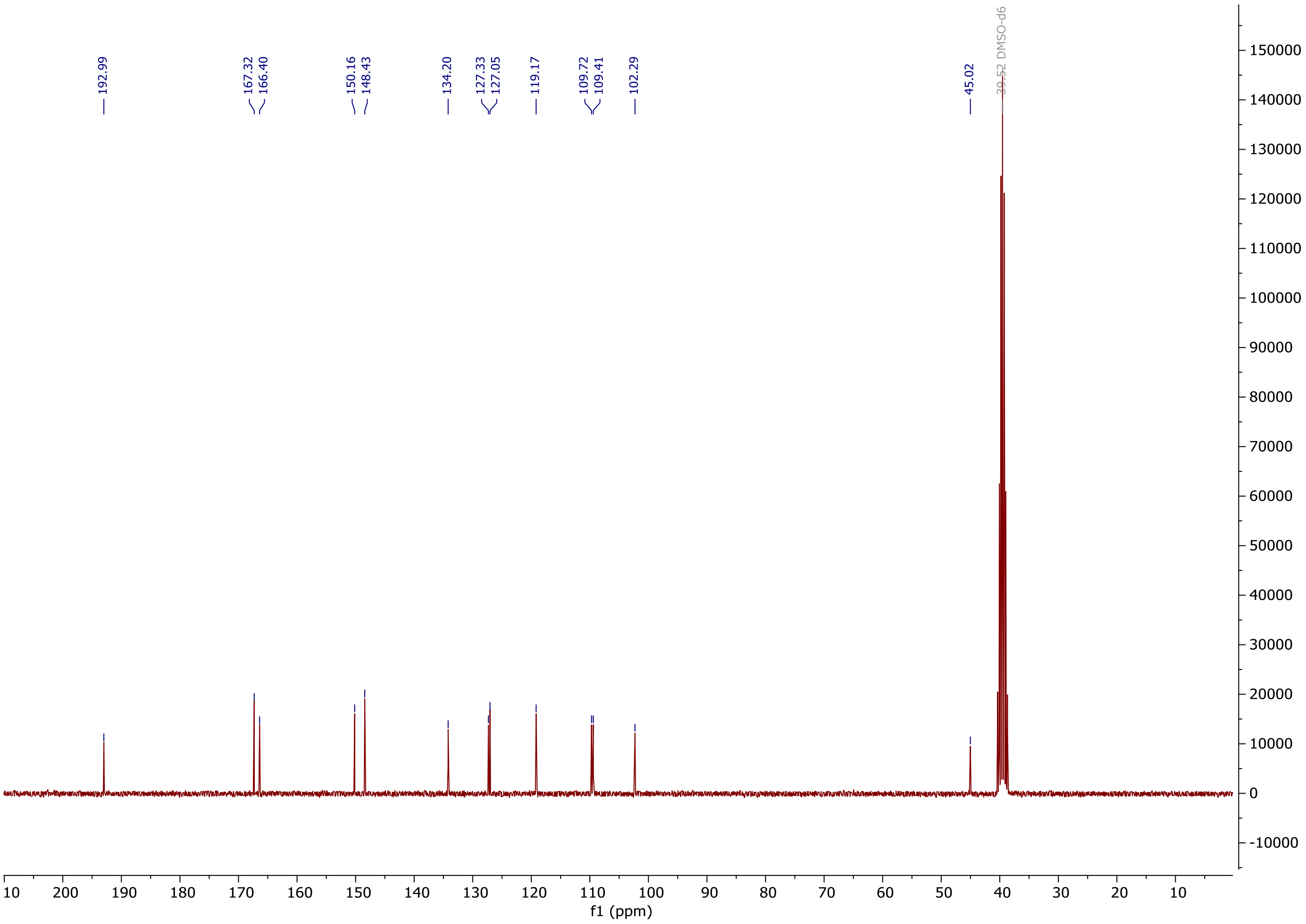

^{1}H NMR (300 MHz, DMSO-d_6) of (5*Z*)-3-(5-benzo[1,3]dioxol-5-ylmethylene-4-oxo-2-thioxo-thiazolidin-3-yl)-propionic acid (**3v**).

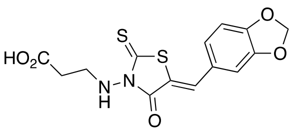

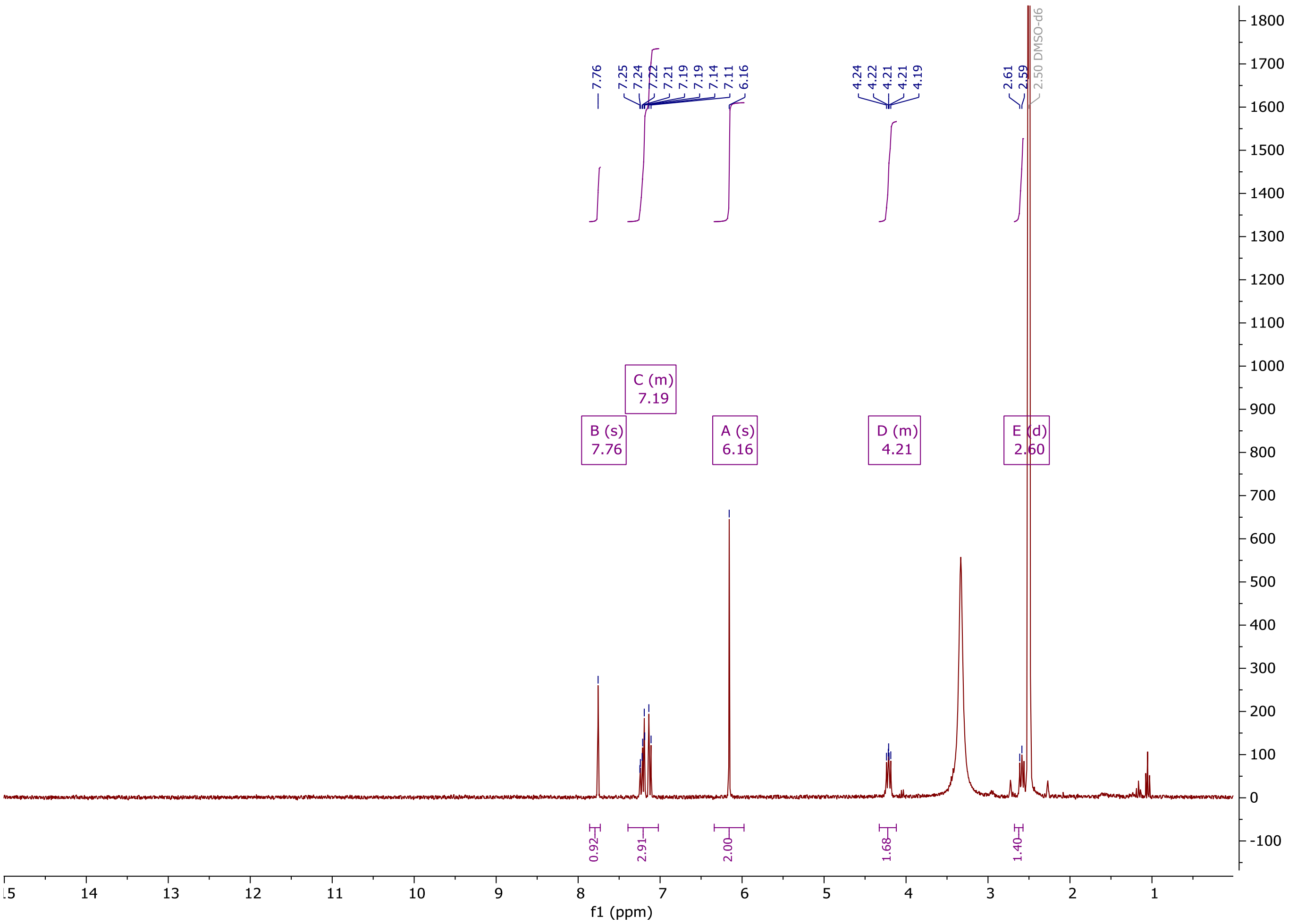

^{13}C NMR (75 MHz, DMSO-*d*$_6$) of (5*Z*)-3-(5-benzo[1,3]dioxol-5-ylmethylene-4-oxo-2-thioxo-thiazolidin-3-yl)-propionic acid (**3v**).

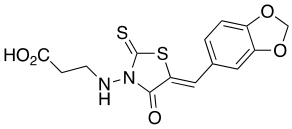

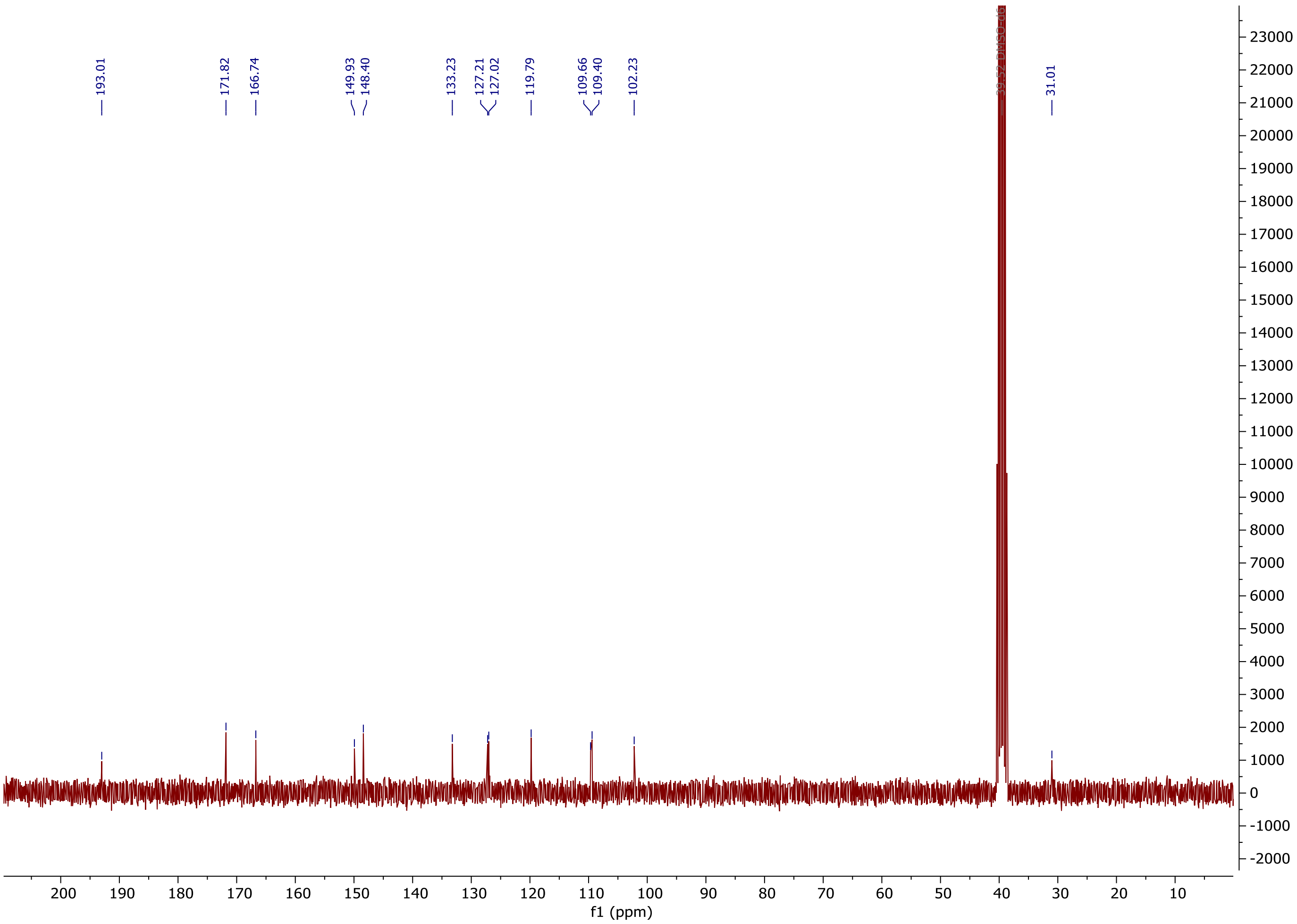

Section 2: ^{1}H and ^{13}C NMR spectra of compounds **5.**

^{1}H NMR (300 MHz, DMSO-d_6) of (5*Z*)-5-(1,3-benzodioxol-5-ylmethylene)-2-(morpholin-1-yl)-1,3-thiazol-4(5*H*)-one (**5a**).

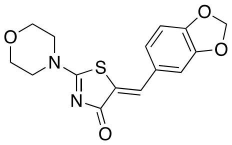

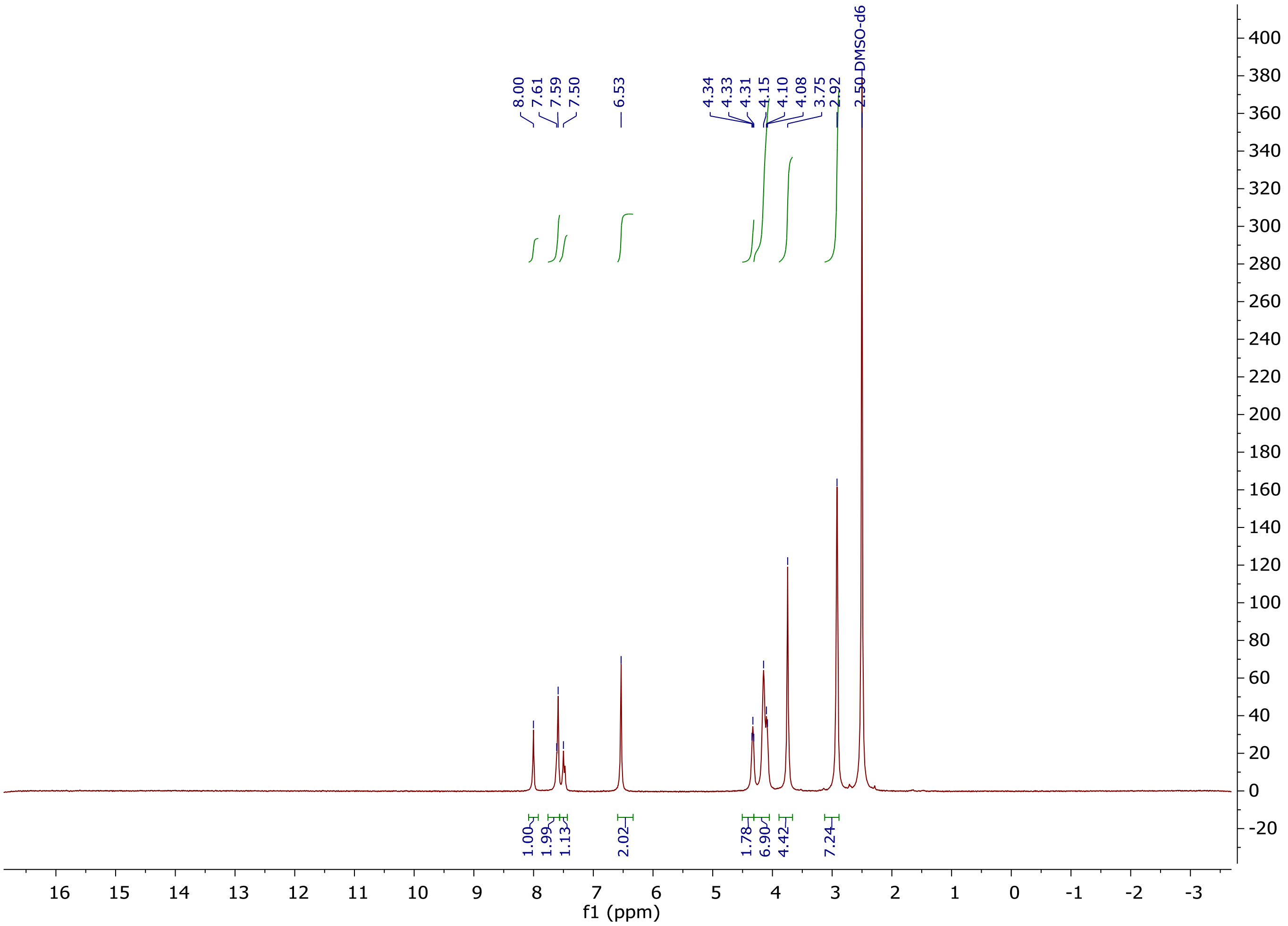

^{1}H NMR (300 MHz, DMSO-*d*$_6$) of

(5*Z*)-5-(1,3-benzodioxol-5-ylmethylene)-2-[1-(4-fluorophenyl)piperazin-1-yl]-1,3-thiazol-4(5*H*)-one (**5k**).

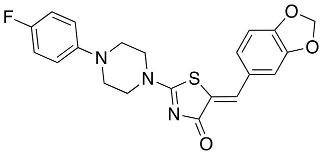

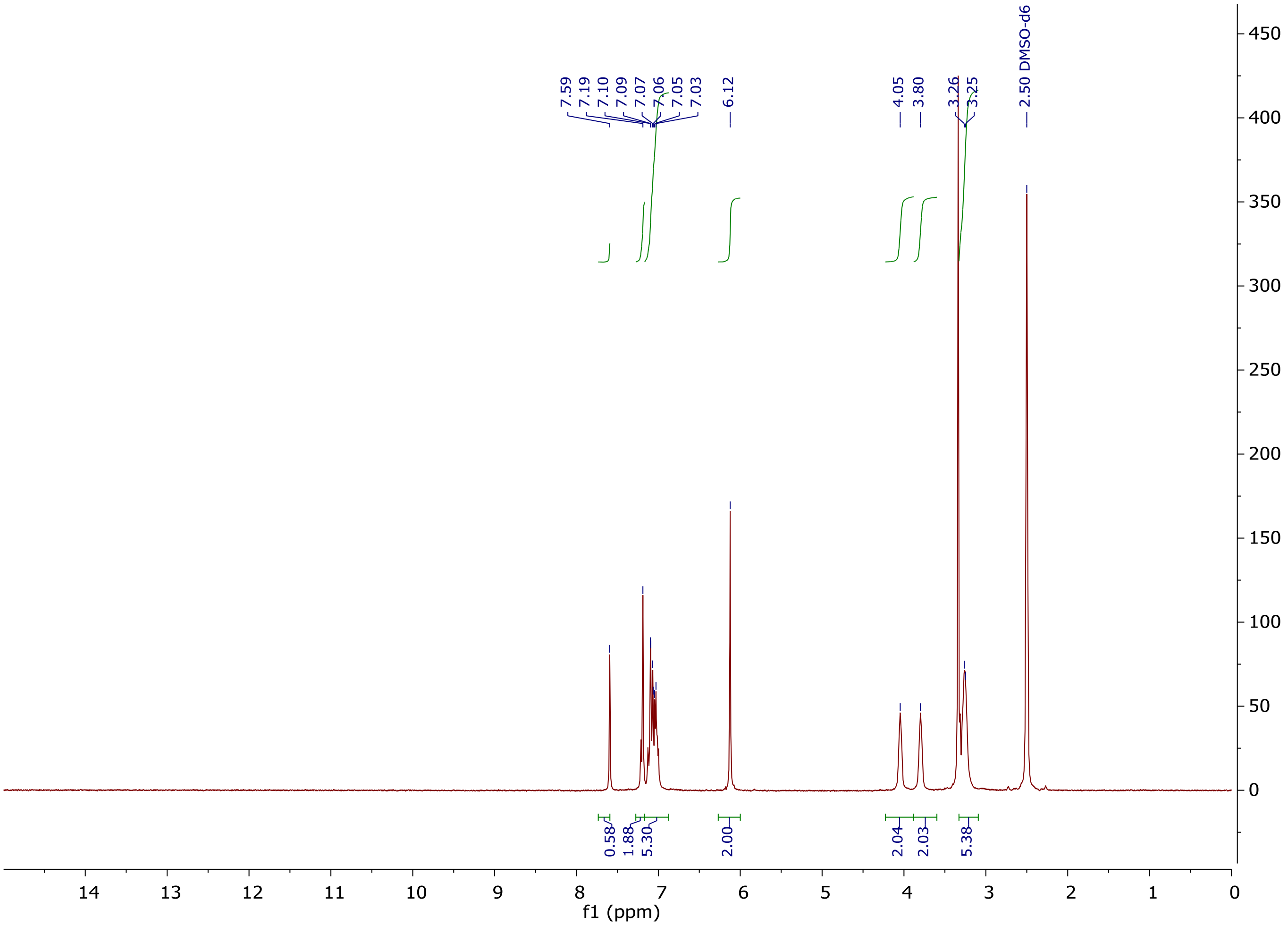

^{1}H NMR (300 MHz, DMSO-d_6) of

(5*Z*)-5-(1,3-benzodioxol-5-ylmethylene)-2-[4-(pyrimidin-2-yl)piperazin-1-yl]-1,3-thiazol-4(5*H*)-one (**5l**).

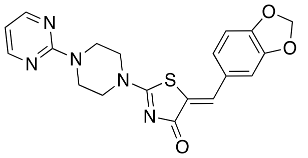

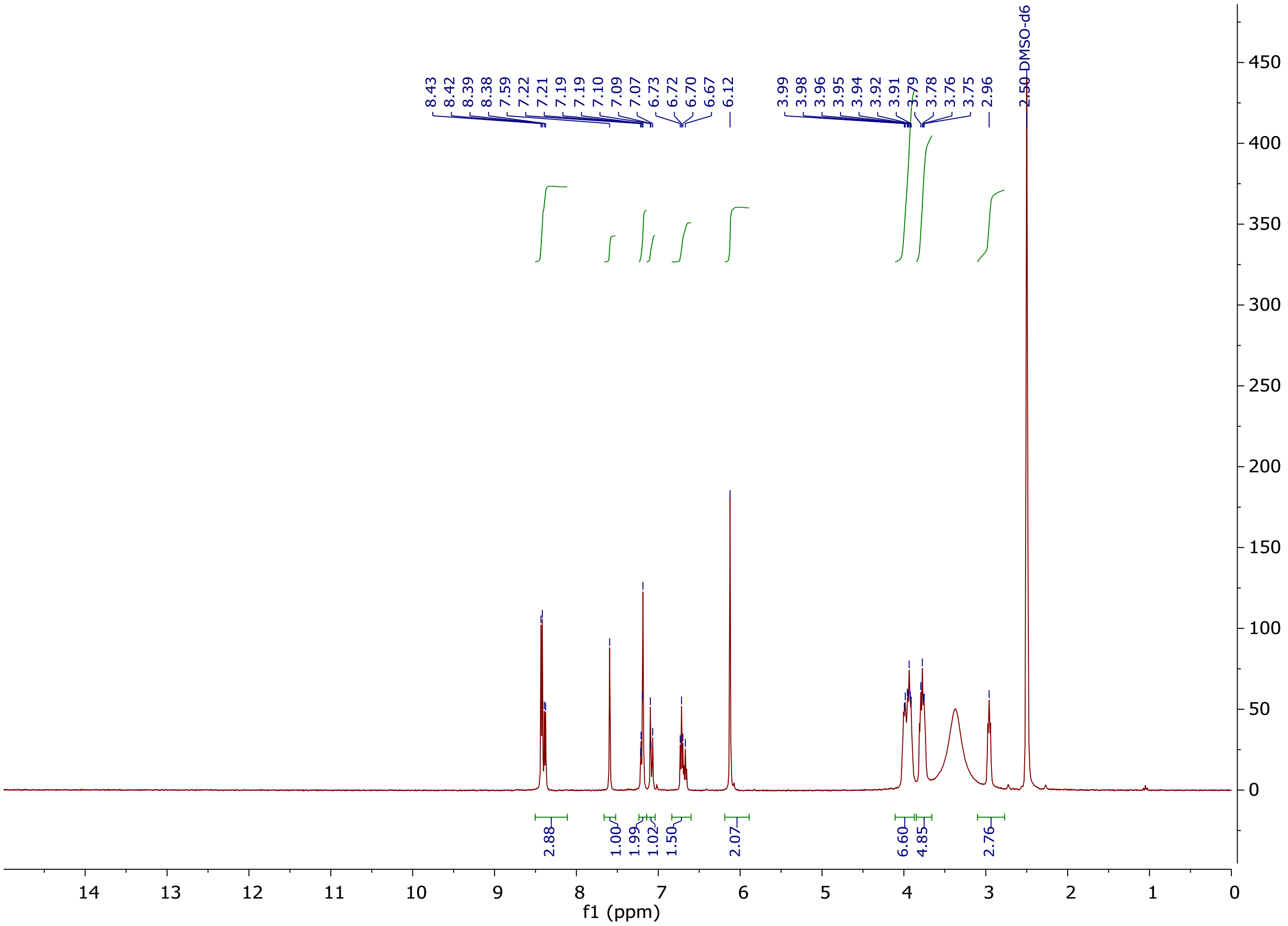

^{1}H NMR (300 MHz, DMSO-d_6) of (5*Z*)-5-benzo[1,3]dioxol-5-ylmethylene-2-phenylamino-1,3-thiazol-4(5*H*)-one (**5r**).

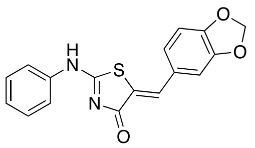

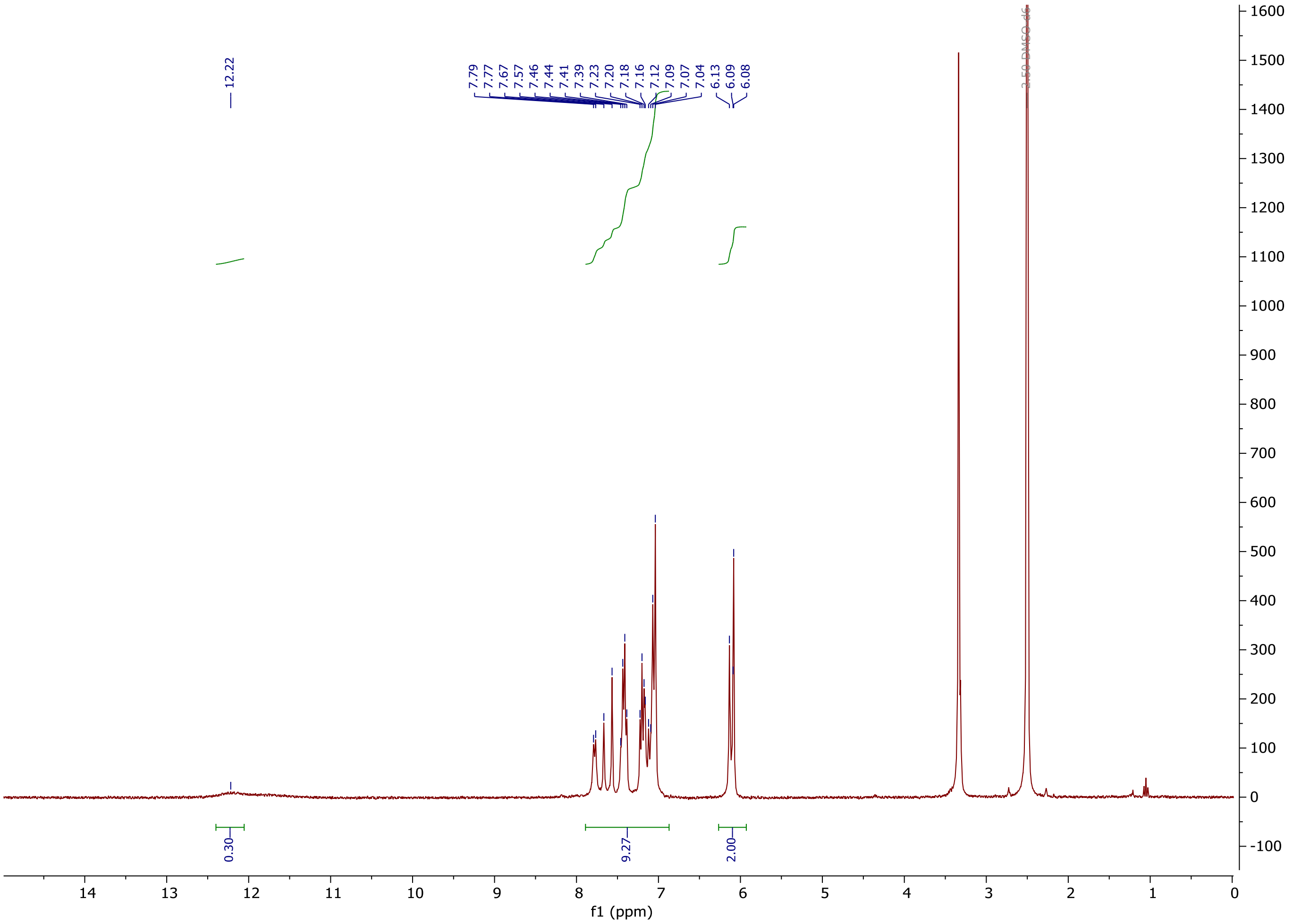

^{13}C NMR (75 MHz, DMSO-*d*$_6$) of (5*Z*)-5-benzo[1,3]dioxol-5-ylmethylene-2-phenylamino-1,3-thiazol-4(5*H*)-one (**5r**).

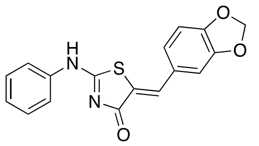

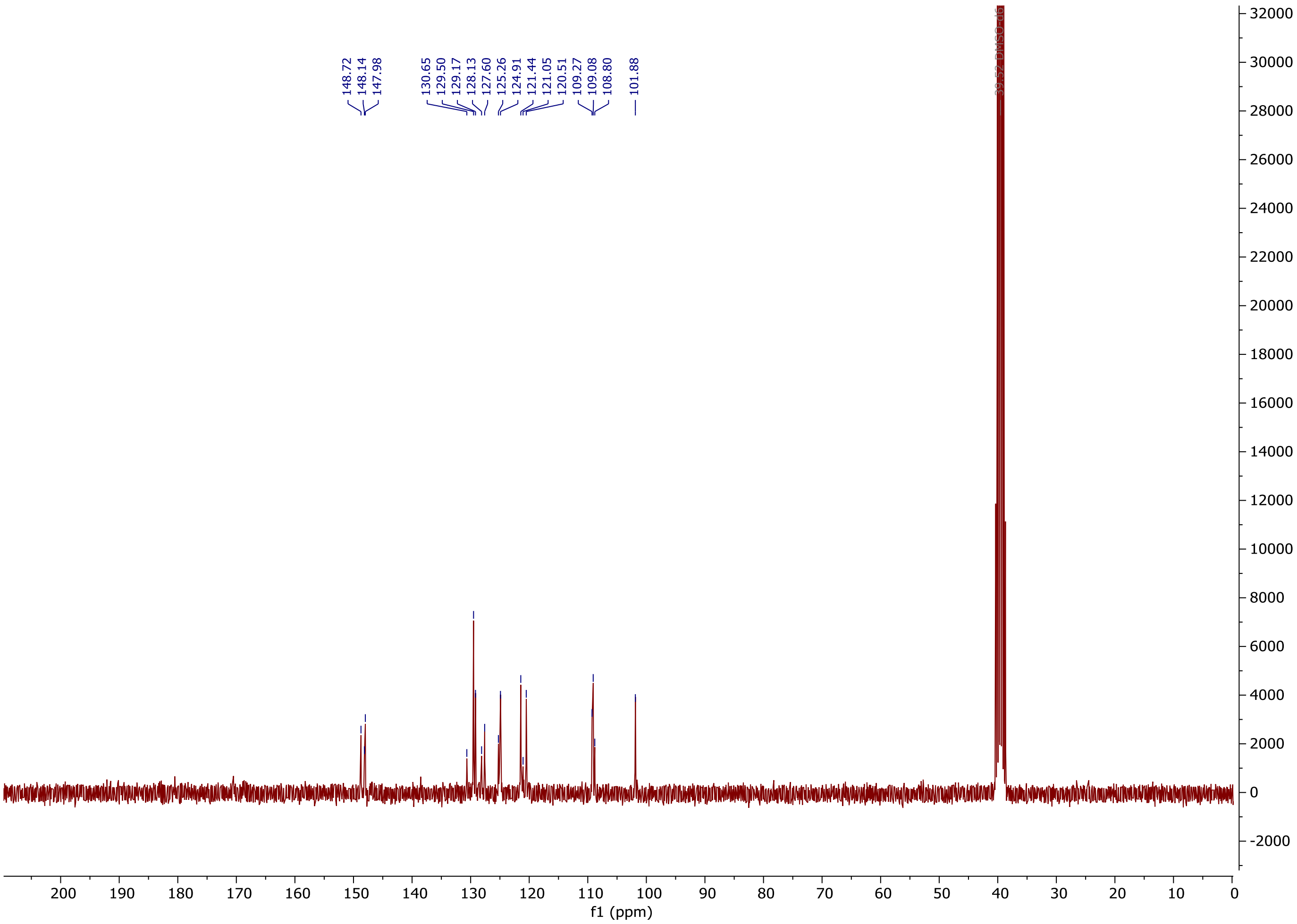

^{1}H NMR (300 MHz, DMSO-d_6) of (5*Z*)-5-benzo[1,3]dioxol-5-ylmethylene-2-(pyridin-2-yl)amino-1,3-thiazol-4(5*H*)-one (**5s**).

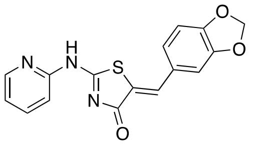

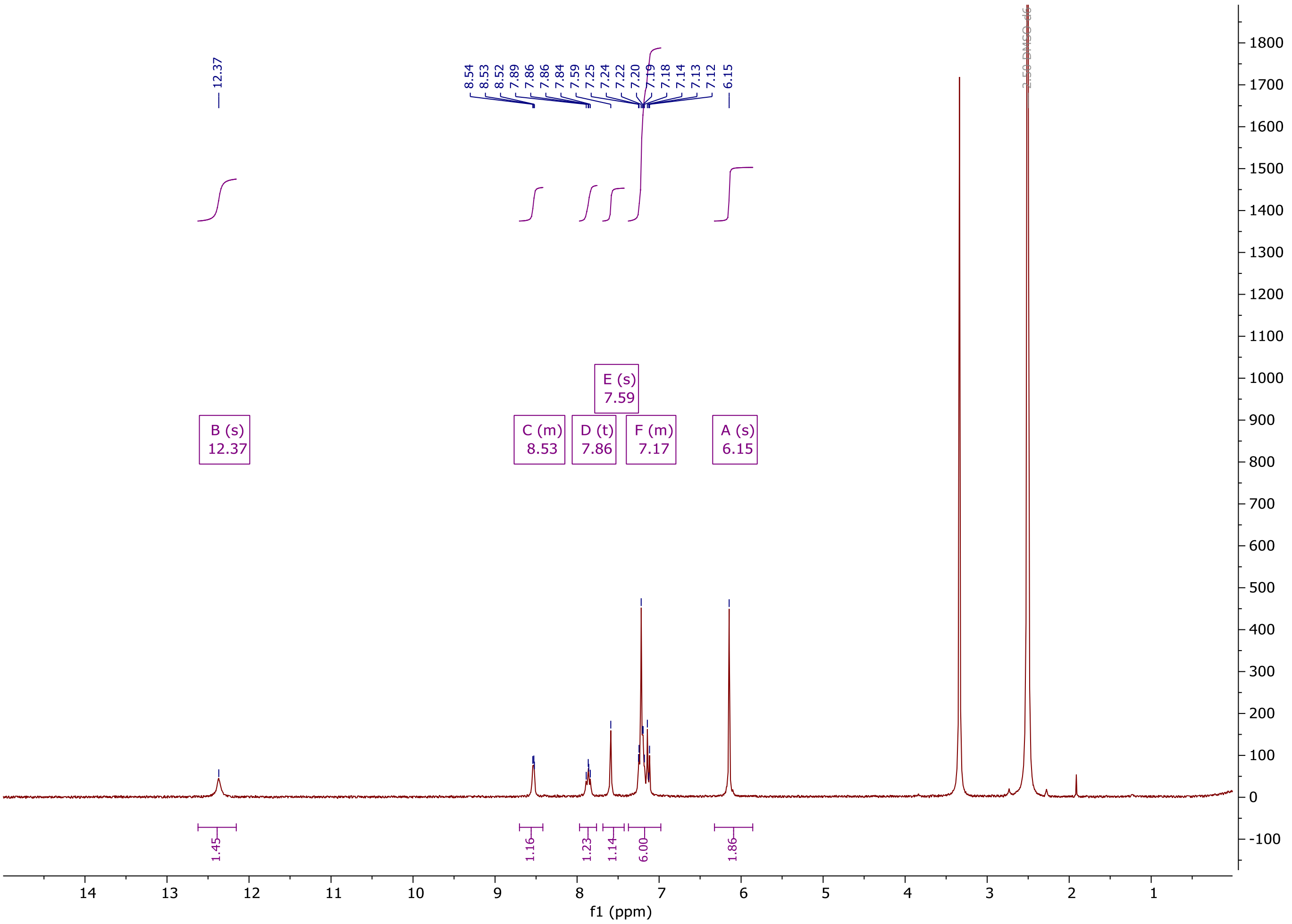

^{13}C NMR (75 MHz, DMSO-d_6) of (5*Z*)-5-benzo[1,3]dioxol-5-ylmethylene-2-(pyridin-2-yl)amino-1,3-thiazol-4(5*H*)-one (**5s**).

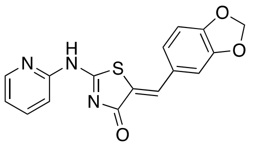

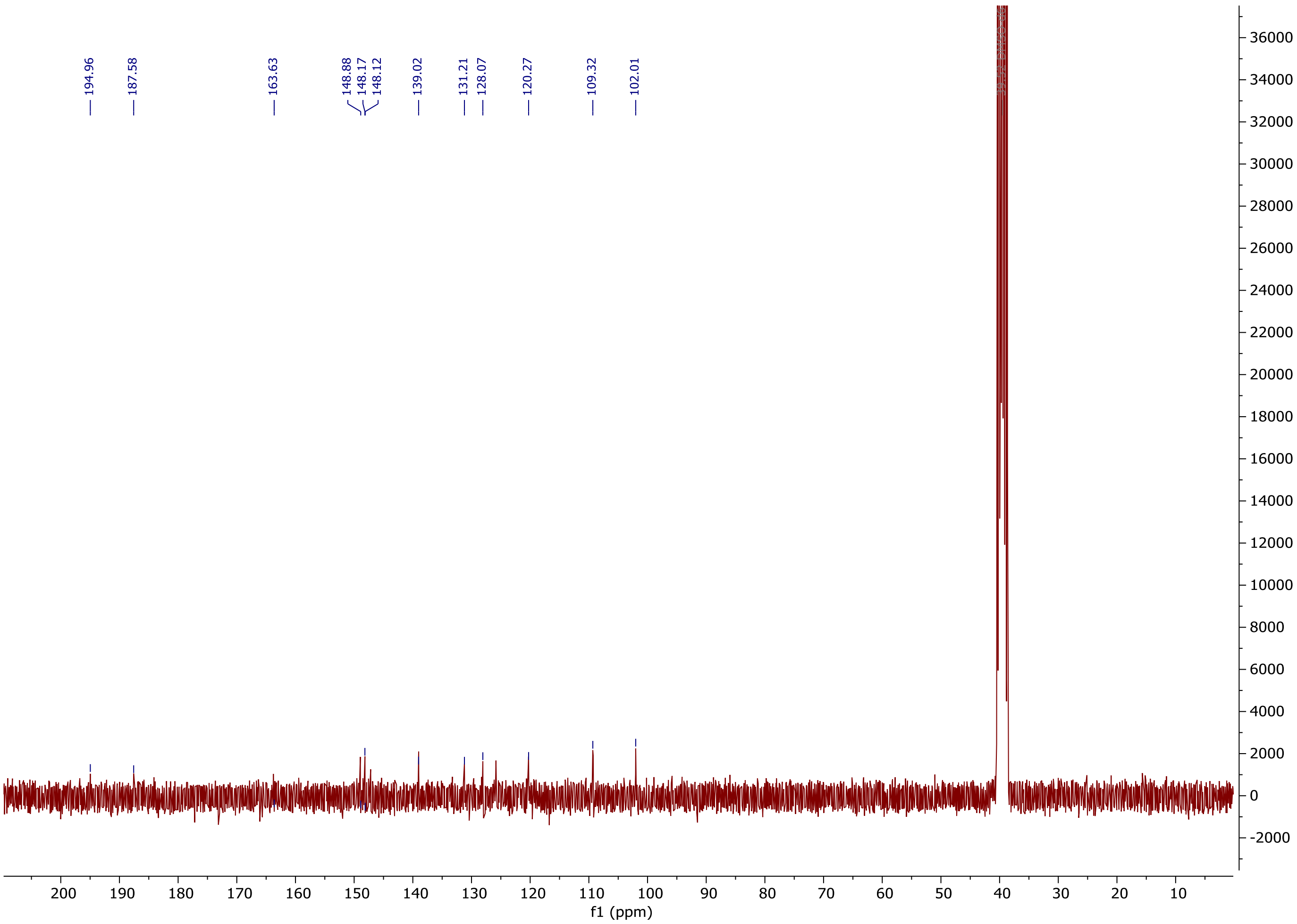

¹H NMR (300 MHz, DMSO-d_6) of (5*Z*)-5-benzo[1,3]dioxol-5-ylmethylene-2-(pyridin-4-yl)amino-1,3-thiazol-4(5*H*)-one (**5t**).

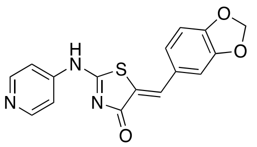

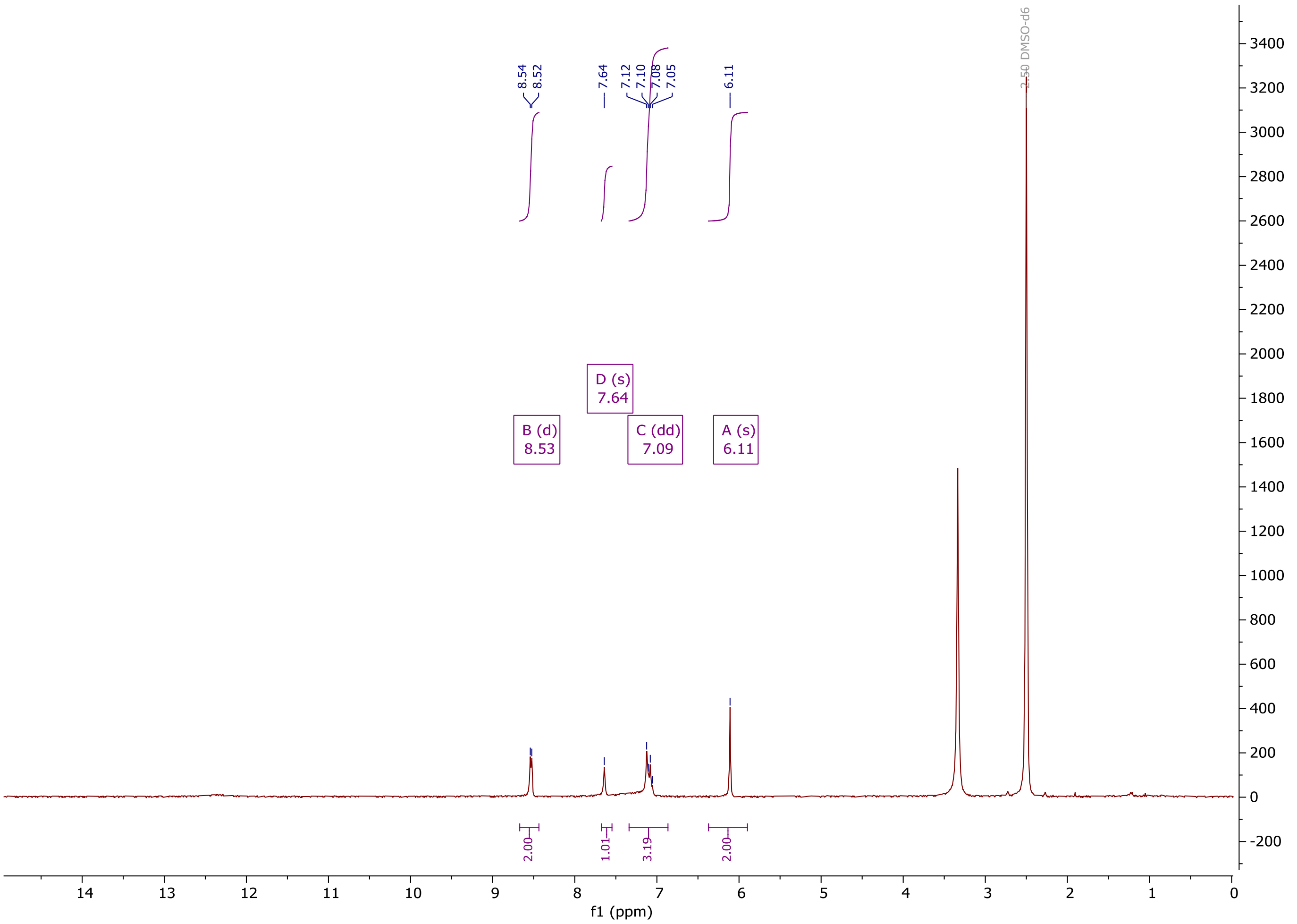

^{13}C NMR (75 MHz, DMSO-d_6) of (5*Z*)-5-benzo[1,3]dioxol-5-ylmethylene-2-(pyridin-4-yl)amino-1,3-thiazol-4(5*H*)-one (**5t**).

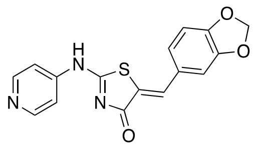

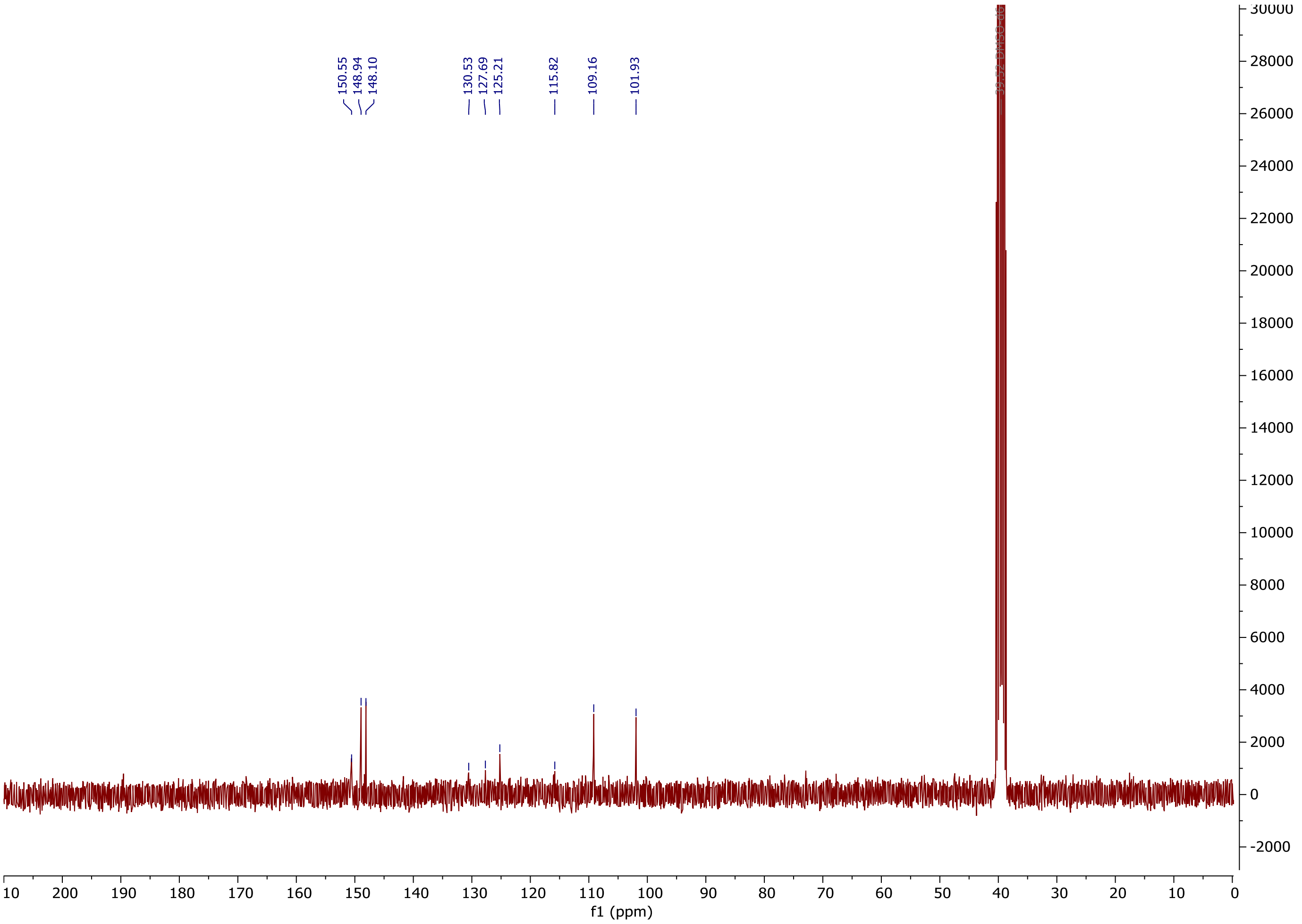

^{1}H NMR (300 MHz, DMSO-d_6) of

(5*Z*)-5-Benzo[1,3]dioxol-5-ylmethylene-2-(4-methoxy-phenylamino)-1,3-thiazol-4(5*H*)-one (**5u**).

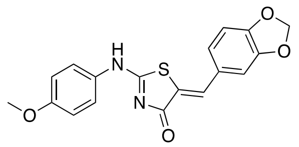

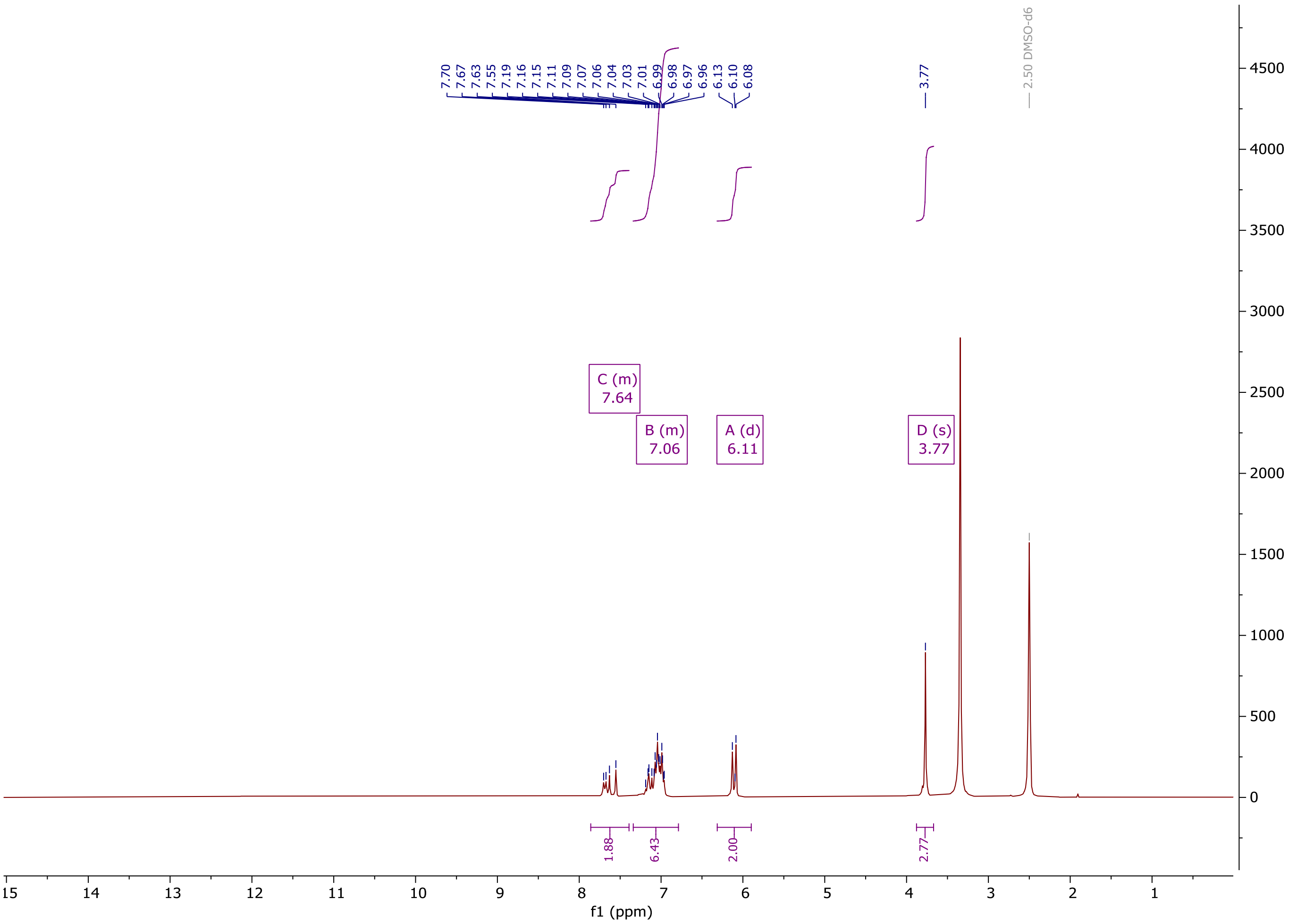

^{13}C NMR (75 MHz, DMSO-*d*$_6$) of

(5*Z*)-5-Benzo[1,3]dioxol-5-ylmethylene-2-(4-methoxy-phenylamino)-1,3-thiazol-4(5*H*)-one (**5u**).

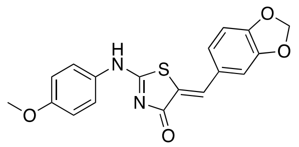

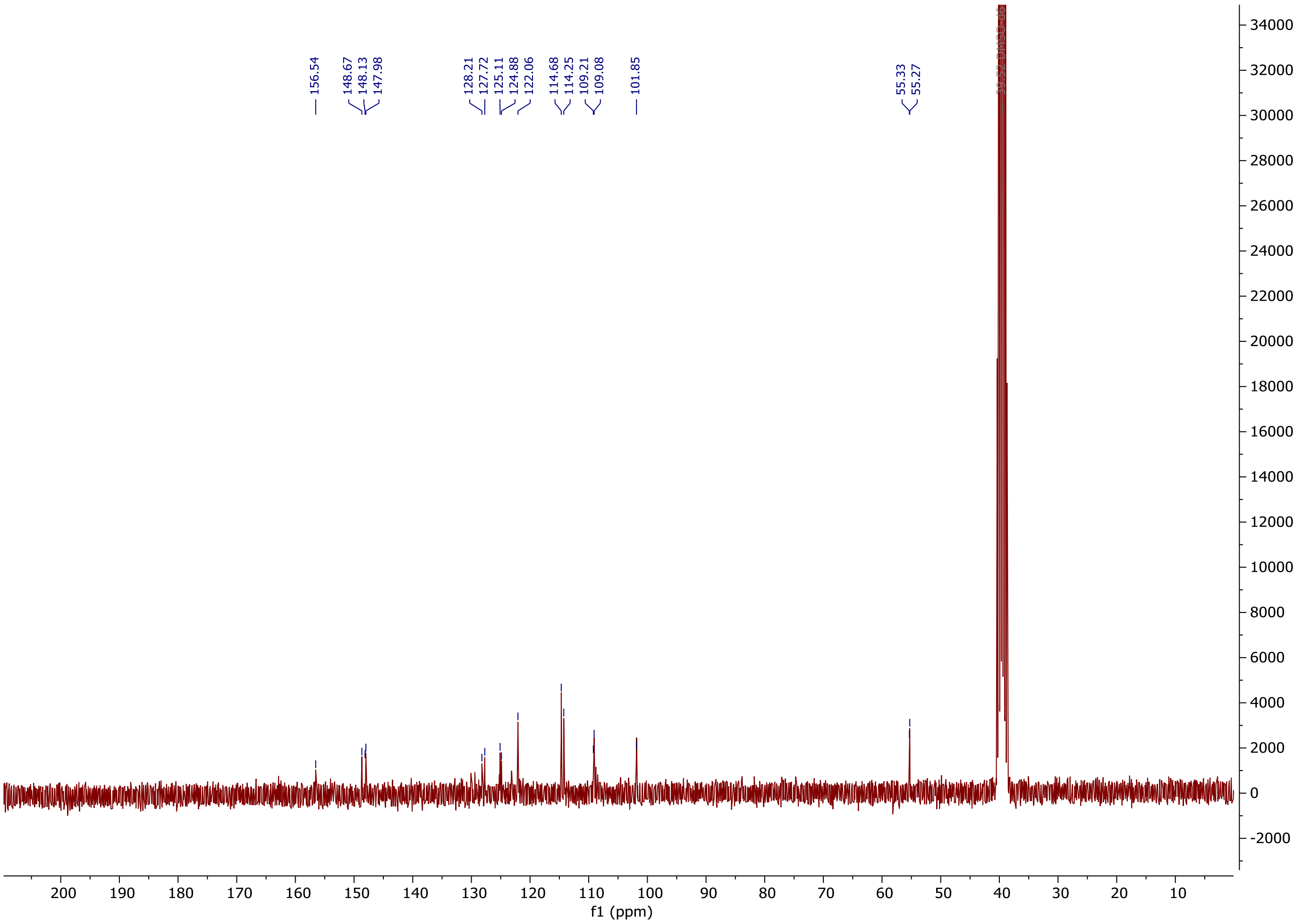

^{1}H NMR (300 MHz, DMSO-d_6) of

(5*Z*)-5-benzo[1,3]dioxol-5-ylmethylene-2-(4-chloro-phenylamino)-1,3-thiazol-4(5*H*)-one (**5v**).

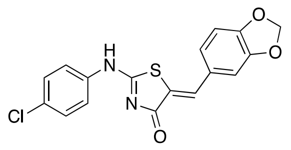

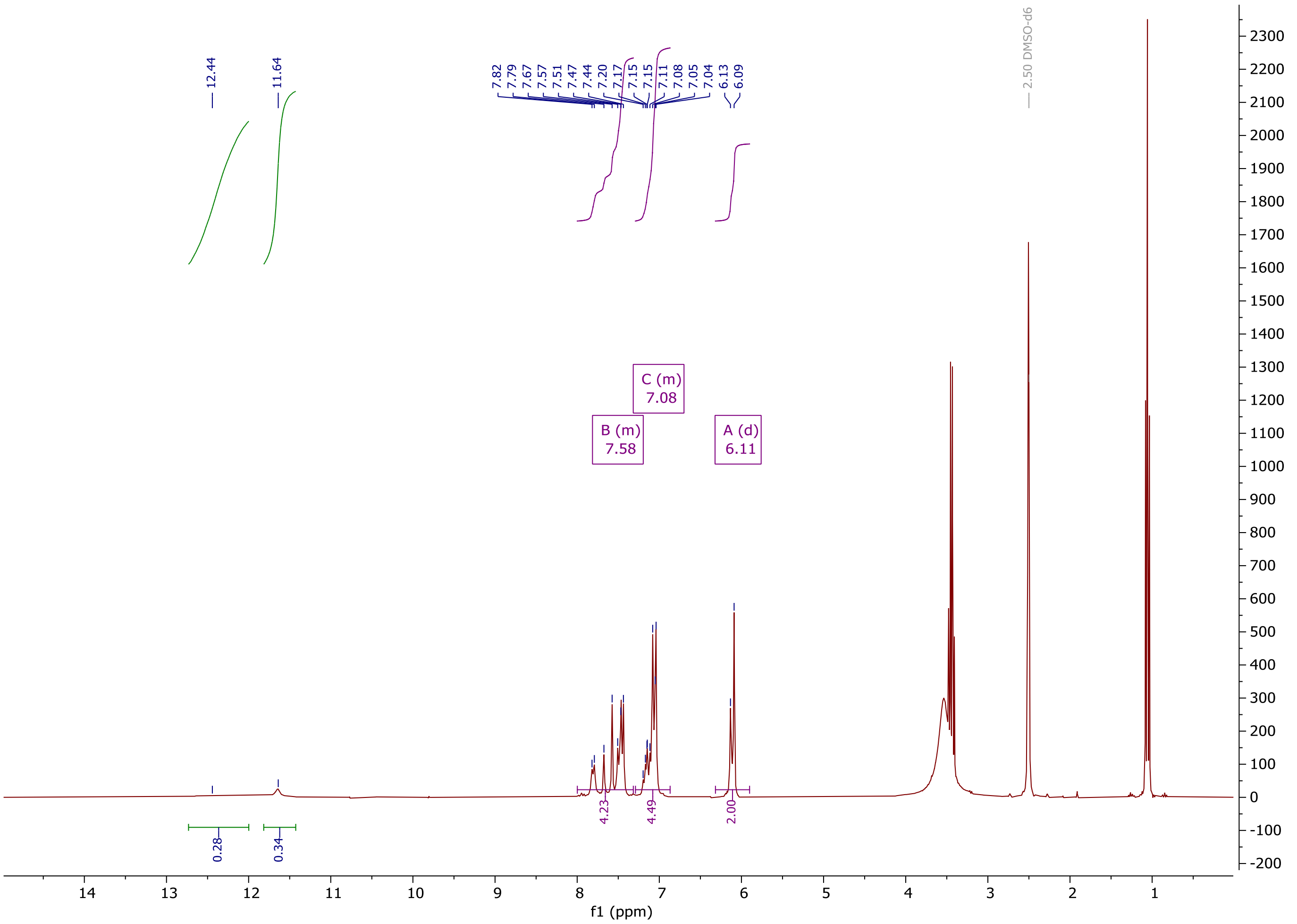

^{13}C NMR (75 MHz, DMSO-*d*$_6$) of
(5*Z*)-5-benzo[1,3]dioxol-5-ylmethylene-2-(4-chloro-phenylamino)-1,3-thiazol-4(5*H*)-one (**5v**).

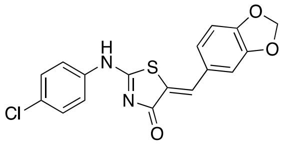

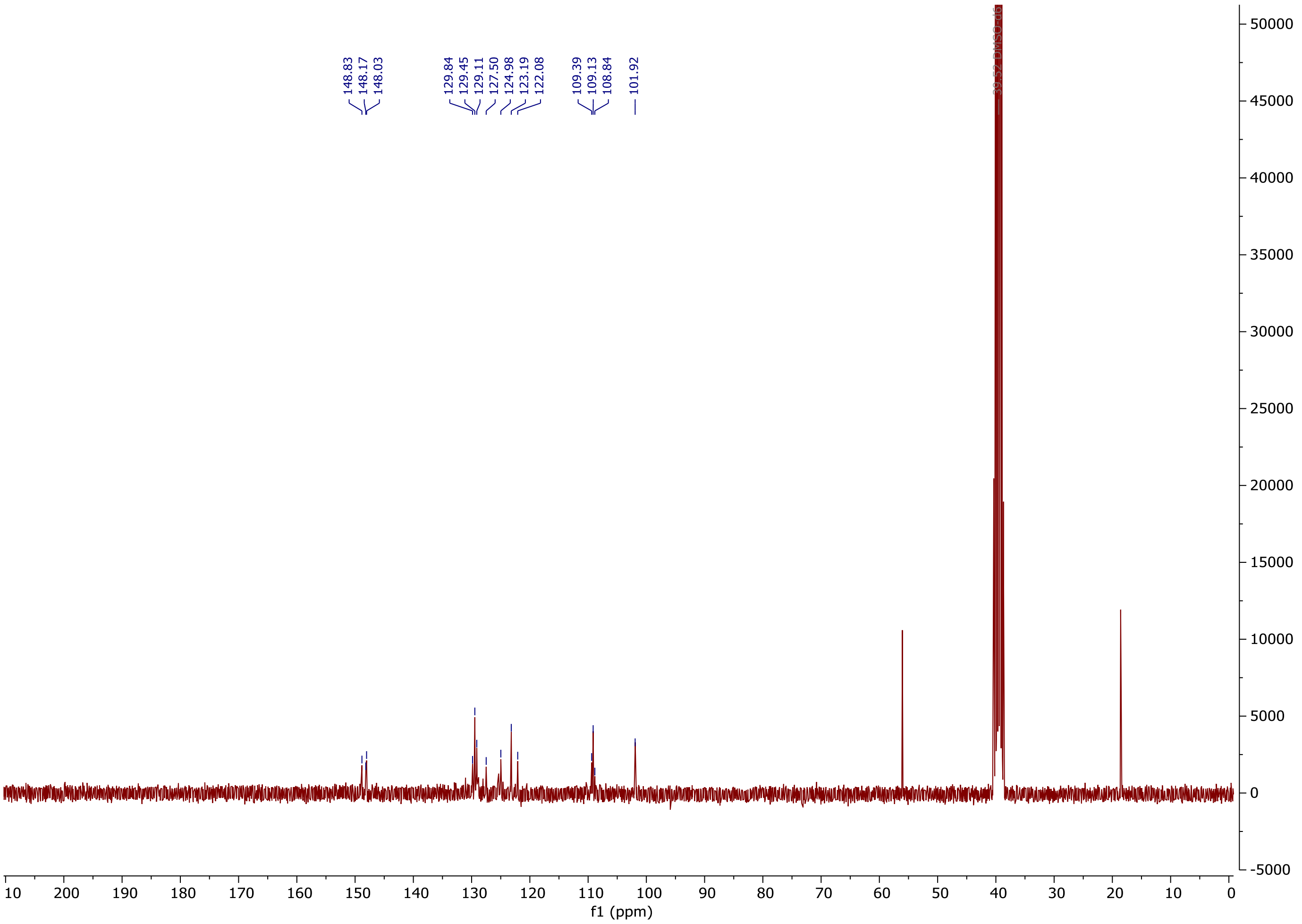

^{1}H NMR (300 MHz, DMSO-d_6) of

(5*Z*)-5-(2,3-dihydro-benzofuran-5-ylmethylene)-2-phenylamino-1,3-thiazol-4(5*H*)-one (**5w**).

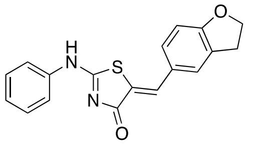

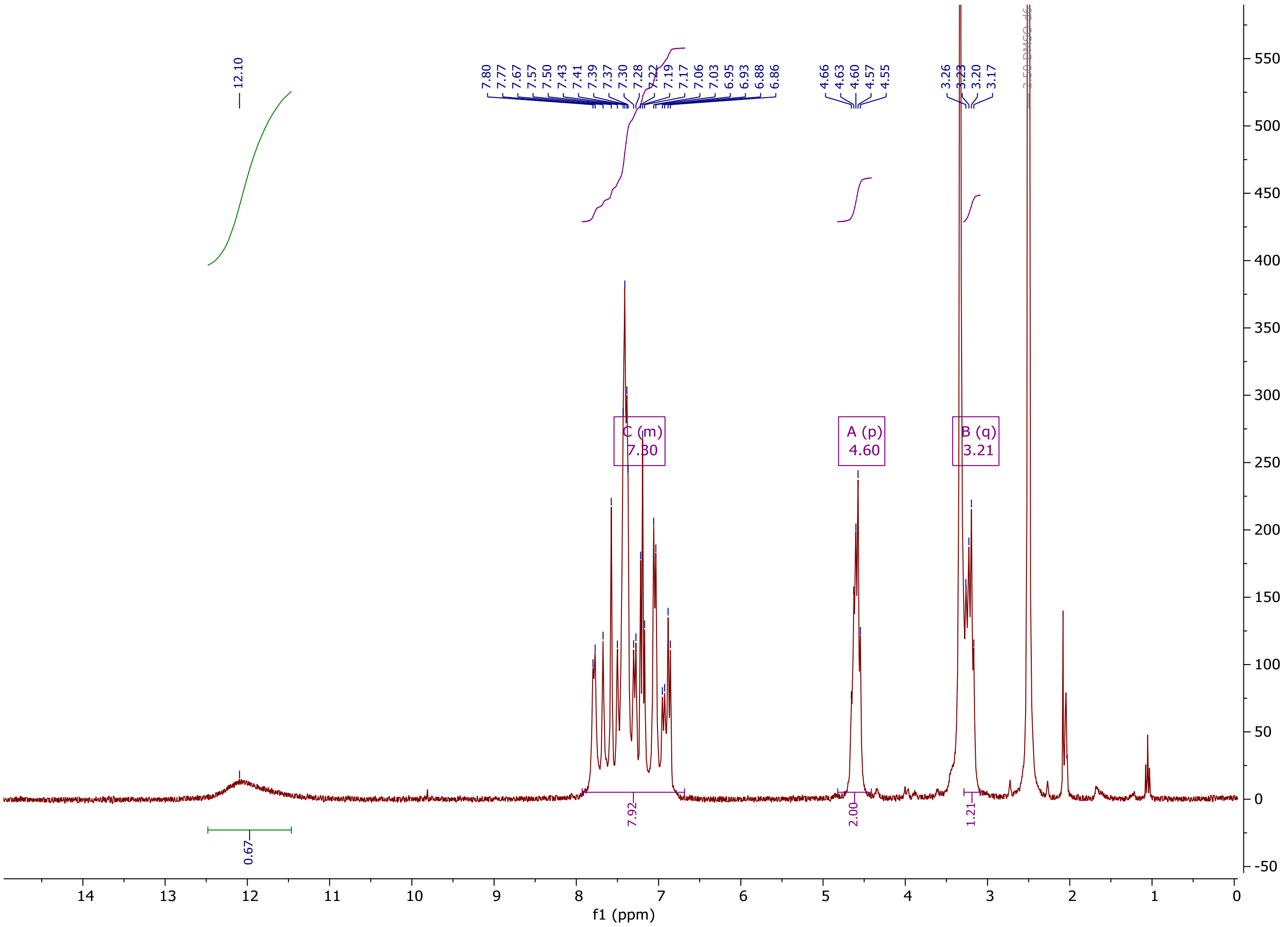

^{13}C NMR (75 MHz, DMSO-d_6) of (5*Z*)-5-(2,3-dihydro-benzofuran-5-ylmethylene)-2-phenylamino-1,3-thiazol-4(5*H*)-one (**5w**).

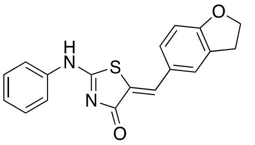

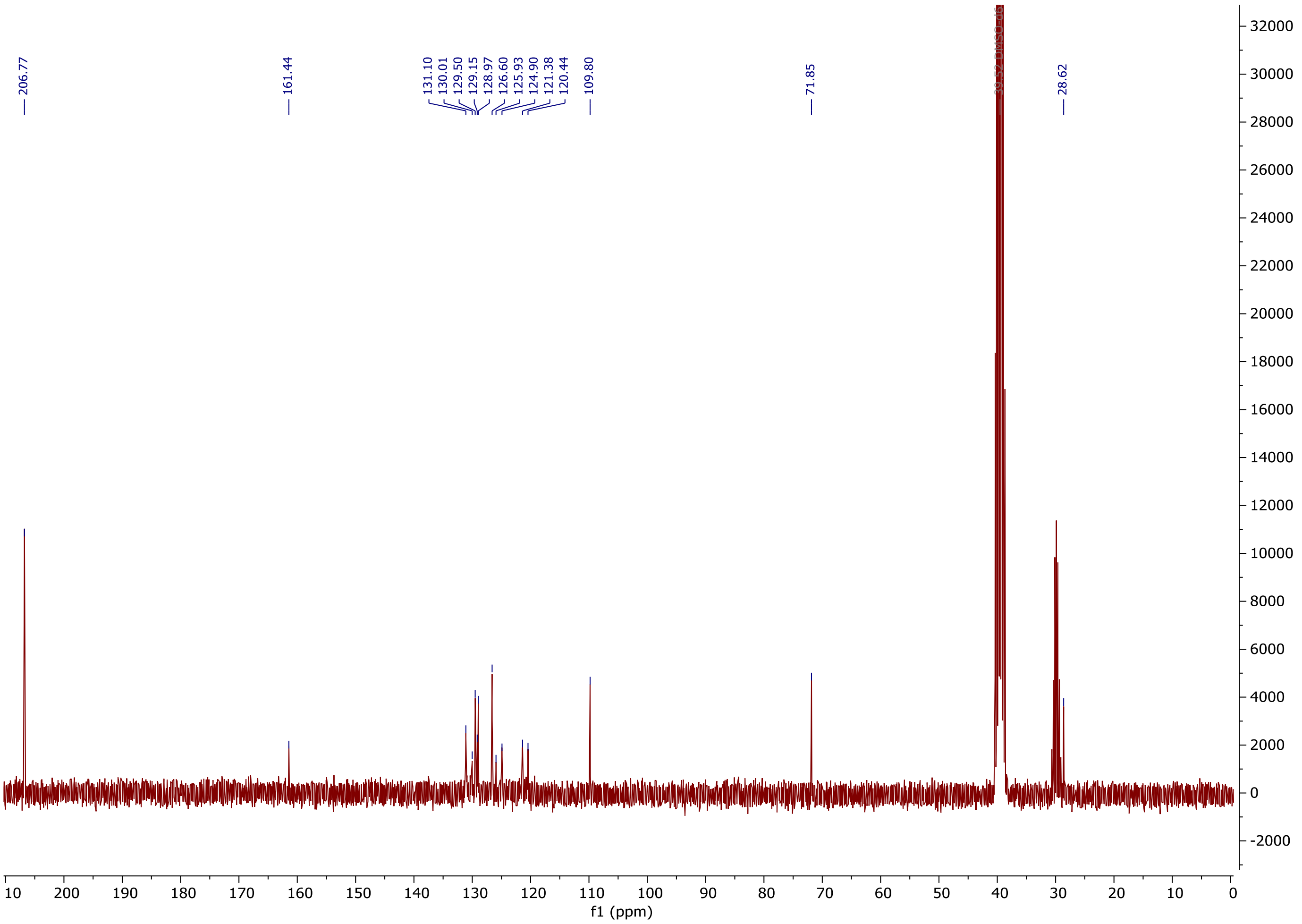

^{1}H NMR (300 MHz, DMSO-d_6) of
(5*Z*)-5-(2,3-dihydro-benzo[1,4]dioxin-6-ylmethylene)-2-phenylamino-1,3-thiazol-4(5*H*)-one (**5x**).

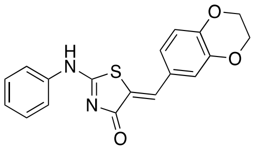

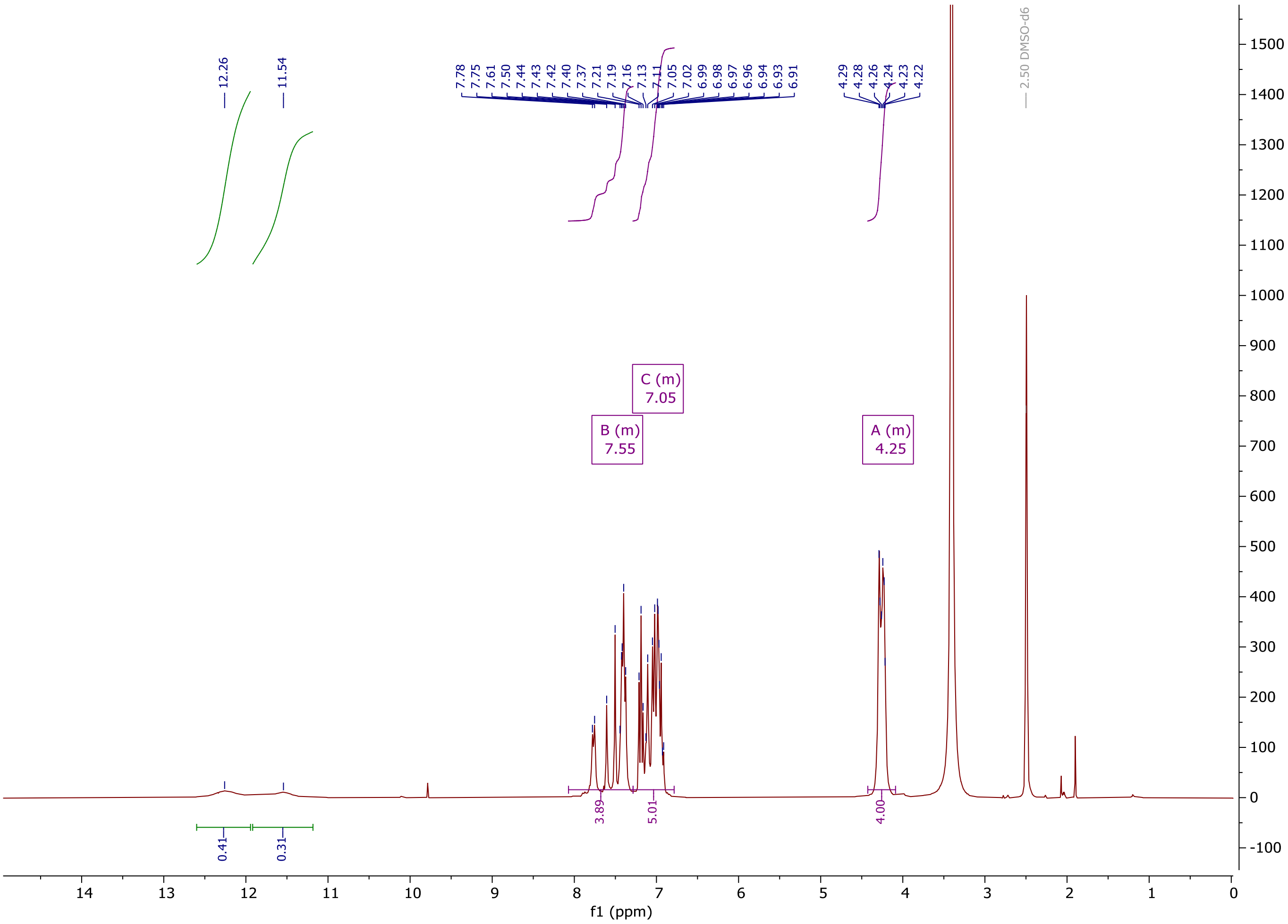

^{13}C NMR (75 MHz, DMSO-d_6) of

(5*Z*)-5-(2,3-dihydro-benzo[1,4]dioxin-6-ylmethylene)-2-phenylamino-1,3-thiazol-4(5*H*)-one (**5x**).

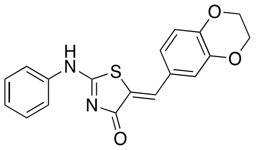

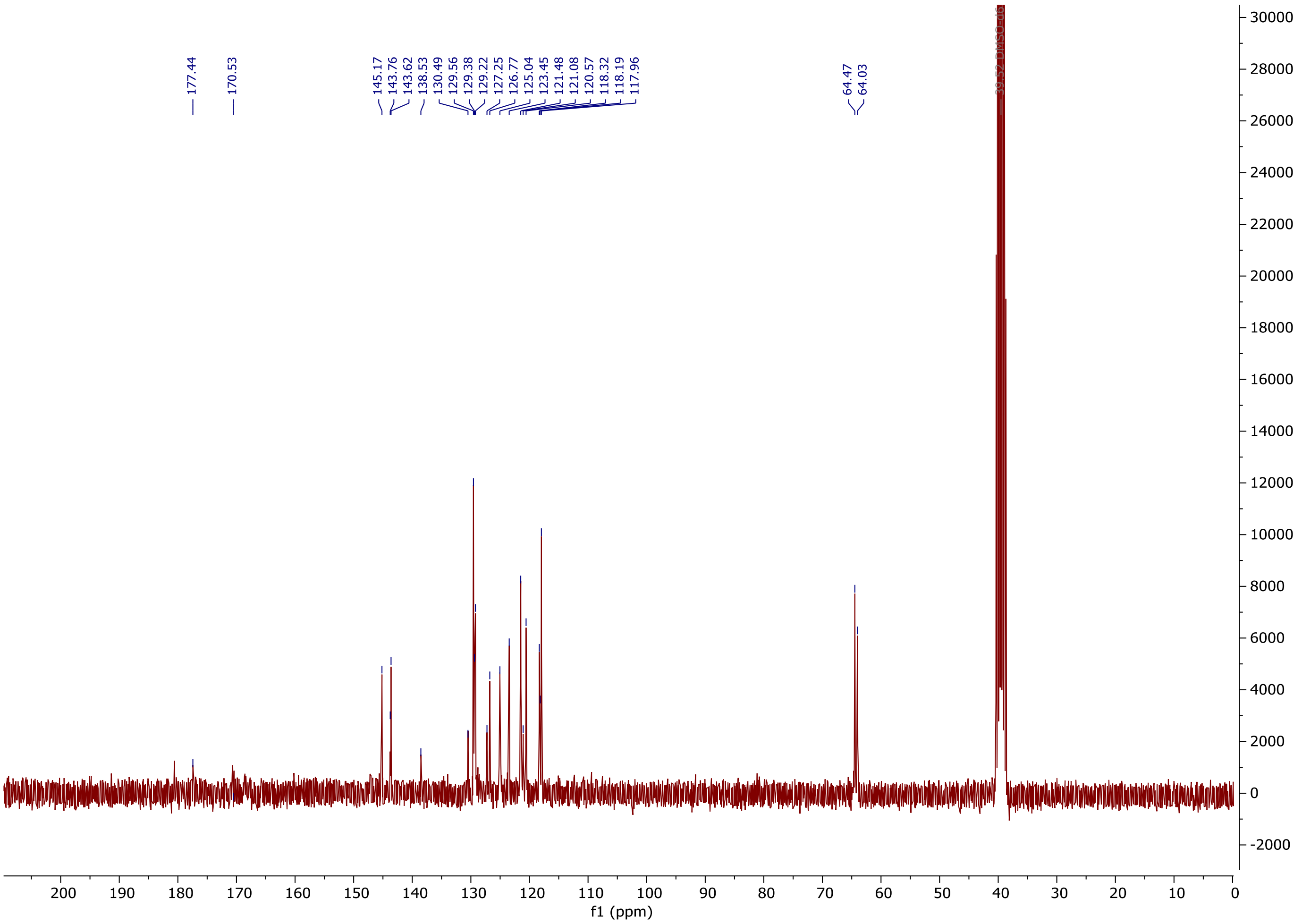

Supplement: Supplementary file 1 [file pharmaceuticals-14-01086-s001.zip › pharmaceuticals-1308289-supplementary.pdf]
